# Supplementary material for: Conserved Pseudoknots in lncRNA MEG3 Are Essential for Stimulation of the p53 Pathway
Source: Mol Cell. 2019 Sep 5;75(5):982–995.e9. doi: 10.1016/j.molcel.2019.07.025 (PMC6739425; doi:10.1016/j.molcel.2019.07.025)
Supplement: Document S1. Figures S1–S7, Tables S1, S3, and S4 [file mmc1.pdf]

Molecular Cell, Volume *75*

Supplemental Information

Conserved Pseudoknots in lncRNA MEG3 Are Essential for Stimulation of the p53 Pathway

Tina Uroda, Eleni Anastasakou, Annalisa Rossi, Jean-Marie Teulon, Jean-Luc Pellequer, Paolo Annibale, Ombeline Pessey, Alberto Inga, Isabel Chillón, and Marco Marcia

SUPPLEMENTAL INFORMATION FILE FOR:

Conserved pseudoknots in lncRNA MEG3 are essential for stimulation of the p53 pathway

Tina Uroda[1], Eleni Anastasakou[1], Annalisa Rossi[2], Jean-Marie Teulon[3], Jean-Luc Pellequer[3], Paolo Annibale[4], Ombeline Pessey[1], Alberto Inga[2], Isabel Chillón[1,*], Marco Marcia[1,*]

[*]To whom correspondence should be addressed. E-mail: ichillon@embl.fr; mmarcia@embl.fr

LEAD AUTHOR: Marco Marcia, mmarcia@embl.fr

The supplemental information file contains:

- Fig. S1 (Related to Fig. 1)
- Fig. S2 (Related to Fig. 1)
- Fig. S3 (Related to Fig. 1)
- Fig. S4 (Related to Fig. 1)
- Fig. S5 (Related to Fig. 3, 4 and 5)
- Fig. S6 (Related to Fig. 5)
- Fig. S7 (Related to Fig. 6)
- Table S1 (Related to Fig. 1, 5 and 6)
- Table S2 (Related to Fig. 2)
- Table S3 (Related to Fig. 4)
- Table S4 (Related to Fig. 4)
- Data File 1 (Related to Fig. 2)

<u>SUPPLEMENTAL FIGURES</u>

Fig. S1: *In vitro* SHAPE structure maps of v1, v3, and v9 (Related to Fig. 1). **A)** *In vitro* secondary structure of v1 color-coded based on the 1M7 and DMS reactivity values of individual nucleotides. Black boxes indicate exon junctions. Helices (H) and multi-way junctions (J) are numbered consecutively from the 5' to the 3'-end. **B-C)** *In vitro* secondary structure representations of v3 and v9, respectively, colored as in panel A (1M7 reactivity values only). Helices and junctions that are different with respect to v1 are assigned a number followed by the variant name (i.e. H1v3).

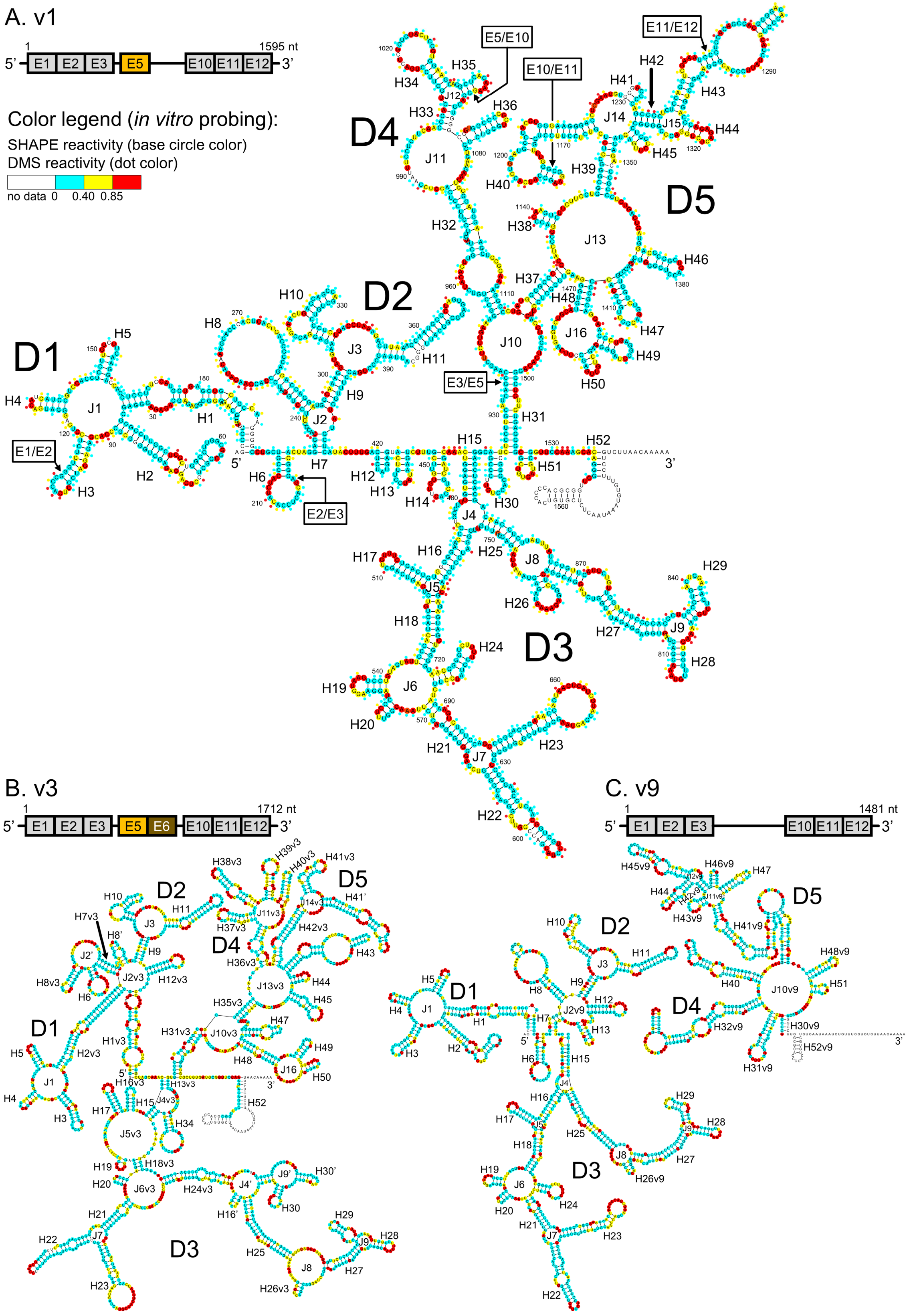
A. v1
5' E1 E2 E3 E5 E10 E11 E12 3'
1
1595 nt
Color legend (in vitro probing):
SHAPE reactivity (base circle color)
DMS reactivity (dot color)
no data 0 0.40 0.85
D1
D2
D3
D4
D5
E1/E2
E2/E3
E3/E5
E5/E10
E10/E11
E11/E12
B. v3
5' E1 E2 E3 E5 E6 E10 E11 E12 3'
1
1712 nt
C. v9
5' E1 E2 E3 E10 E11 E12 3'
1
1481 nt

Fig. S2: *In vitro* SHAPE probing of v1, v3, and v9 (Related to Fig. 1). **A-C)** *In vitro* SHAPE structures of v1, v3, and v9. From top to bottom, for each variant: exon and domain architecture; 1M7 and Shannon entropy reactivity profile (we note that Shannon entropy calculations in Superfold use 50-nt-sliding windowed medians, not single nucleotide resolution); base-pairing probabilities; and minimum free-energy structures. *In vitro* reactivity values of individual nucleotide of all three variants and corresponding SEM from n=3 experiments are reported in **Figure S3**. Spearman's correlation coefficient (CC_S) between replicas are indicated on the right. **D)** CC_S between splicing variants. **E)** CC_S between replicas of H11LpA. **F)** Average and median Shannon entropy values (S_{ave} and S_{median}, respectively) and median SHAPE reactivity (R_{median}) for each variant.

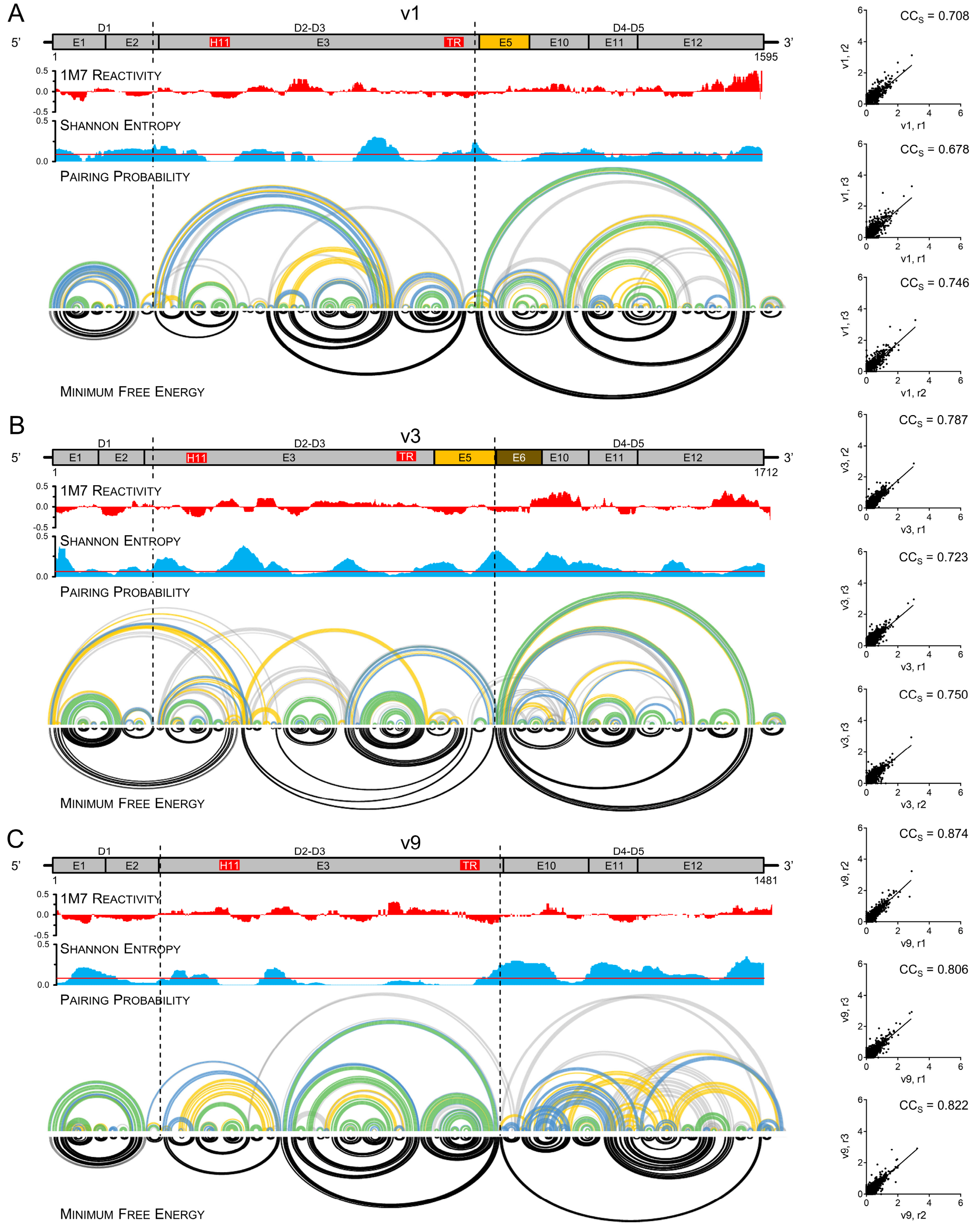

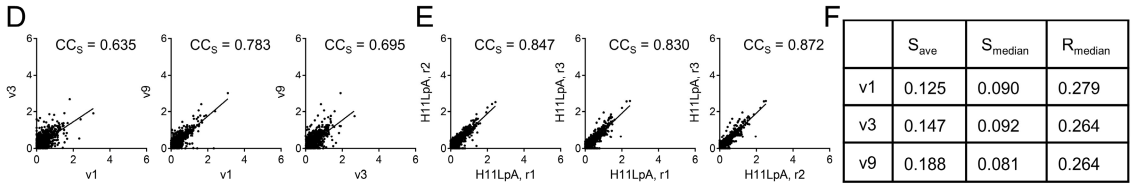

F

	S_{ave}	S_{median}	R_{median}
v1	0.125	0.090	0.279
v3	0.147	0.092	0.264
v9	0.188	0.081	0.264

Fig S3: *In vitro* SHAPE probing of v1, v3, and v9 (Related to Fig. 1). The graph reports 1M7 reactivity values of individual nucleotides. Error bars indicate SEM from n = 3 experiments. The scale of the y-axis is set to match the color scale of **Figure S1**. Nucleotides with very high reactivity values (> 1.2) are excluded for clarity of visualization. The x-axis reports nucleotide numbers for the longest variant, v3. Black boxes indicate exon junctions, gray labels and arrows indicate domains, and red boxes indicate the position of H11 and TR1-6.

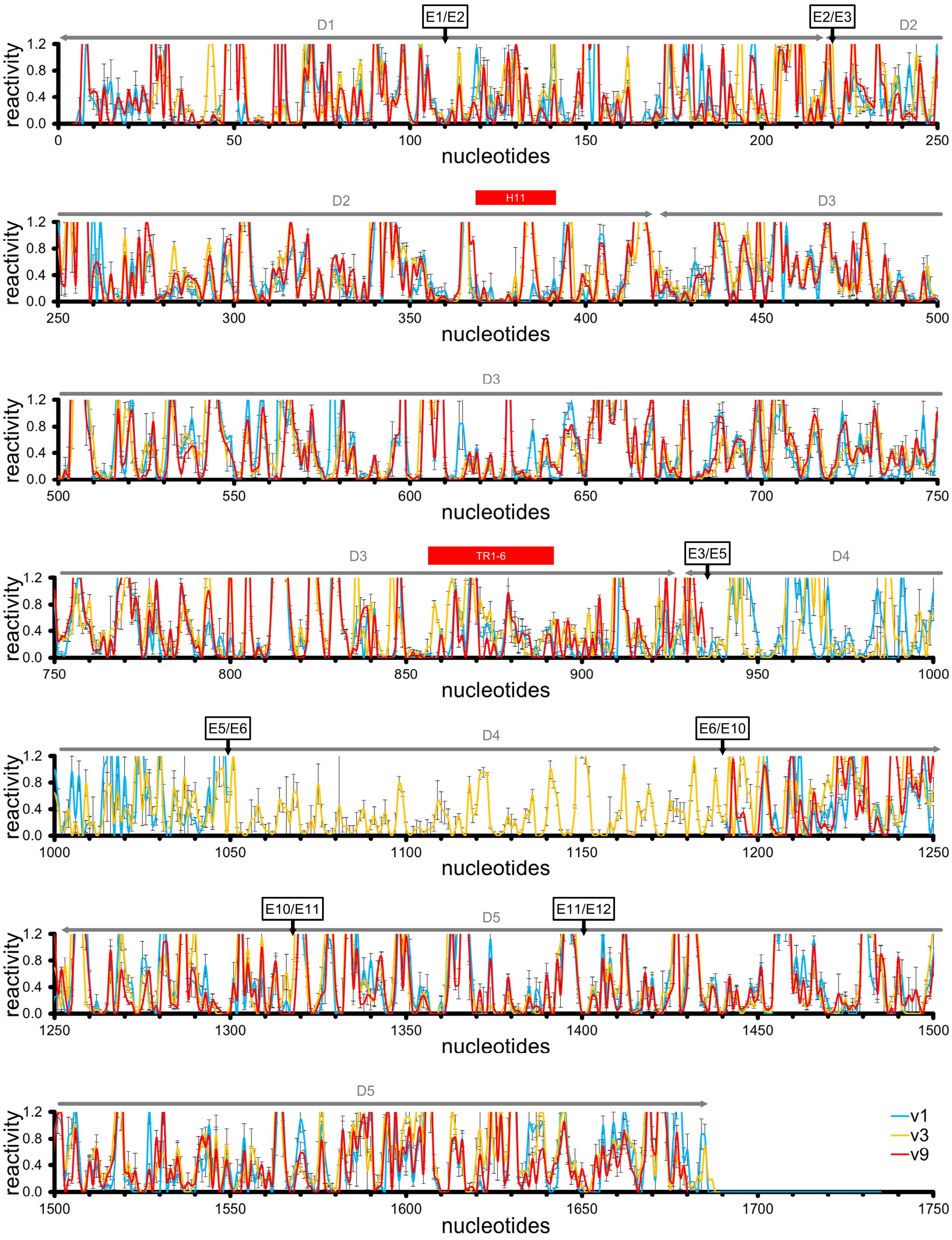
D1
E1/E2
E2/E3
D2
reactivity
nucleotides
H11
D3
TR1-6
E3/E5
D4
E5/E6
E6/E10
E10/E11
E11/E12
D5
v1
v3
v9

Fig S4: Structure ensemble of the MEG3 core (Related to Fig. 1). Ensemble of 9 secondary structure maps of the D2-D3 core produced by RNAStructure using only 1M7 reactivity values of v1.

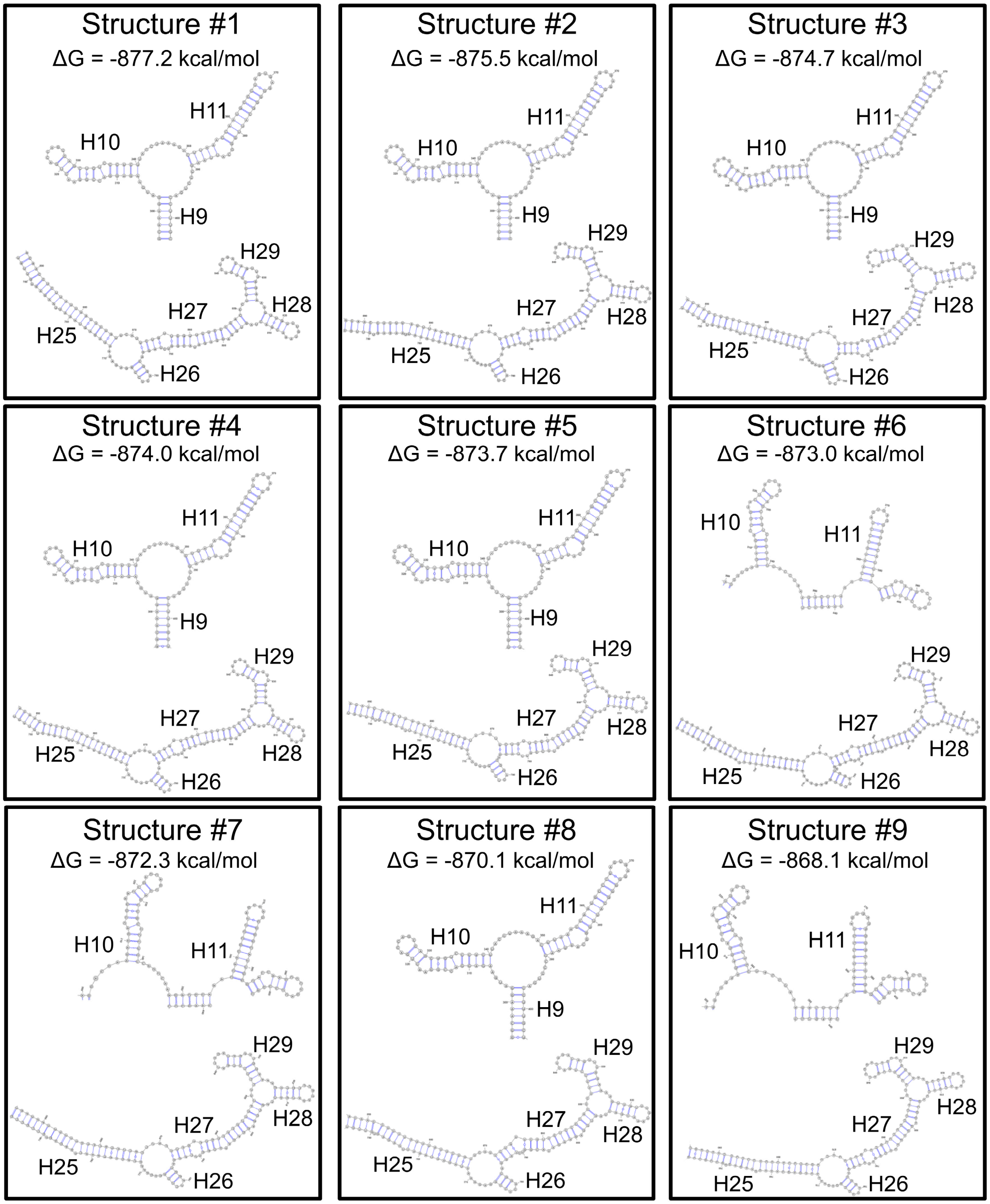

Fig. S5: Effects of MEG3 on cell cycle and transactivation of p53 target genes (Related to Fig. 3, 4 and 5). **A-I)** Distribution of GFP$^-$ cell populations for the flow cytometry assays described in **Figure 3**. **J-M)** Optimization of the luciferase assay (all data points are normalized to the signal of corresponding control vectors, set to a y-axis value of 1). DNA titration (J) and time course performed with v1 (K). At 60 h cells are not healthy anymore. Stimulation of the p53 pathway by v1 transcribed from pCI and pcDNA3 vectors (L). Time course in which Firefly luciferase values have been normalized with Renilla luciferase or beta-galactosidase signals as transfection controls (M). **N)** Quantification by qRT-PCR the abundance of endogenous MEG3 relative to actin mRNA (ACTB) in HCT116 and WI38. **O)** Quantification by qRT-PCR of the abundance of all mutants used for luciferase assays. All constructs were transfected in HCT116 cells and expressed under the same conditions used for functional assays. Abundance of mRNA of each MEG3 construct was calculated relative to actin (loading control) and neomycin (transfection control) mRNAs. Values are expressed as fold changes with respect to the abundance of v1. Construct D5 is indicated in parentheses because it is > 10000 fold less expressed than v1. Error bars indicate SEM from n = 3 (panels A-N) or n = 2 experiments (panel O).

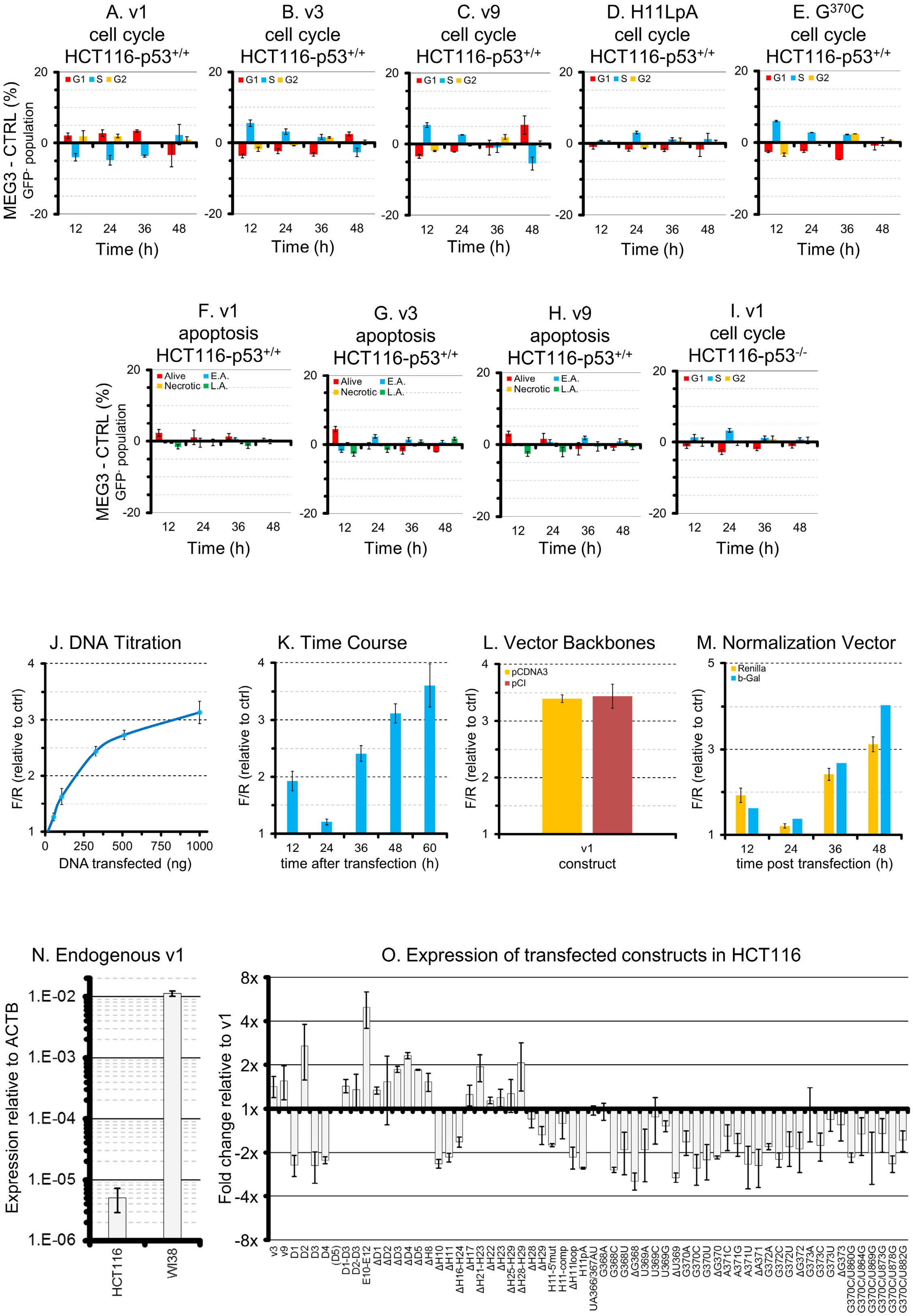
A. v1 cell cycle HCT116-p53+/+
B. v3 cell cycle HCT116-p53+/+
C. v9 cell cycle HCT116-p53+/+
D. H11LpA cell cycle HCT116-p53+/+
E. G370C cell cycle HCT116-p53+/+
MEG3 - CTRL (%) GFP- population
G1 S G2
Time (h)
F. v1 apoptosis HCT116-p53+/+
G. v3 apoptosis HCT116-p53+/+
H. v9 apoptosis HCT116-p53+/+
I. v1 cell cycle HCT116-p53-/-
Alive E.A. Necrotic L.A.
J. DNA Titration
F/R (relative to ctrl)
DNA transfected (ng)
K. Time Course
time after transfection (h)
L. Vector Backbones
pCDNA3
pCI
v1 construct
M. Normalization Vector
Renilla
b-Gal
time post transfection (h)
N. Endogenous v1
Expression relative to ACTB
HCT116
WI38
O. Expression of transfected constructs in HCT116
Fold change relative to v1

Fig. S6: HRF probing of v1 and H11LpA (Related to Fig. 5). **A)** HRF reactivity values of individual nucleotides of v1 and H11LpA. Error bars indicate SEM from n = 3 experiments. The scale of the y-axis is set to match the color scale of **Figure 5C**. Nucleotides with very high reactivity values (> 1.2) are excluded for clarity of visualization. Black boxes indicate exon junctions. **B)** $CC_{Spearman}$ between HRF replicas of v1 and H11LpA (r1-r3). **C)** Comparison of number of secondary structure motifs (top) and solvent-protected nucleotides (bottom) in v1 *vs* other indicated structured RNAs. The number of secondary structure motifs of each RNA has been normalized to the size (number of nucleotides) of MEG3 v1.

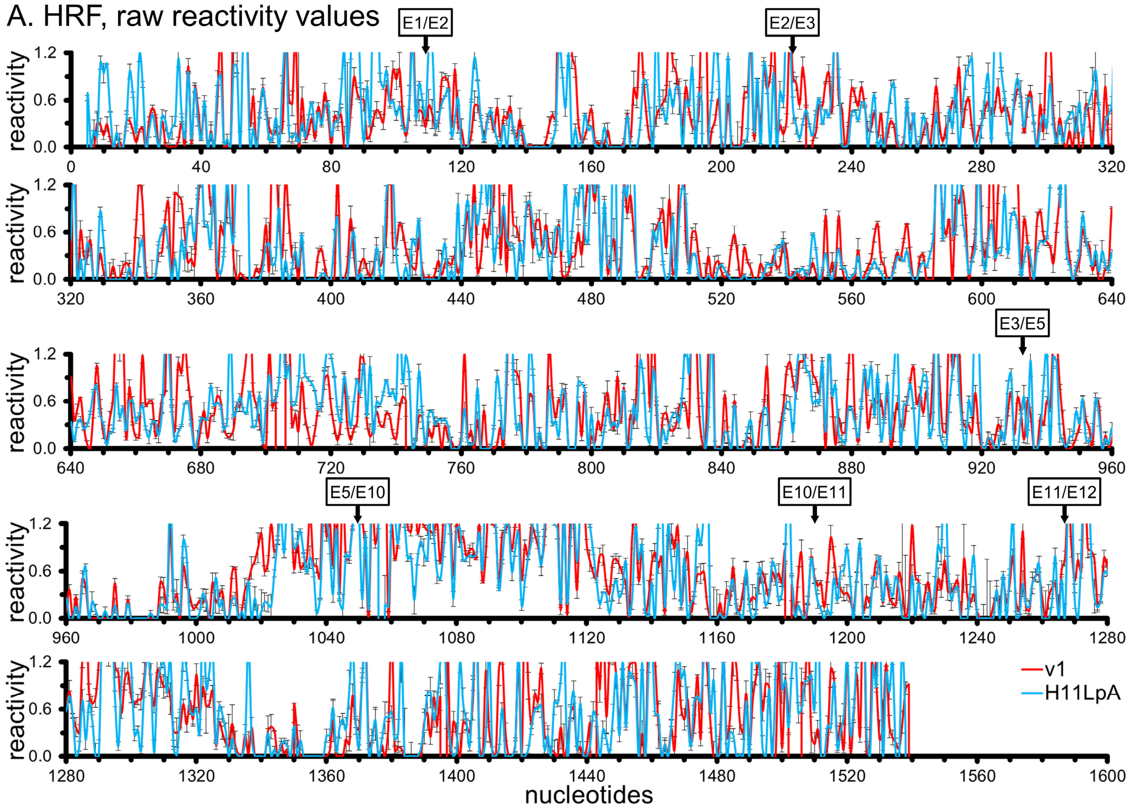

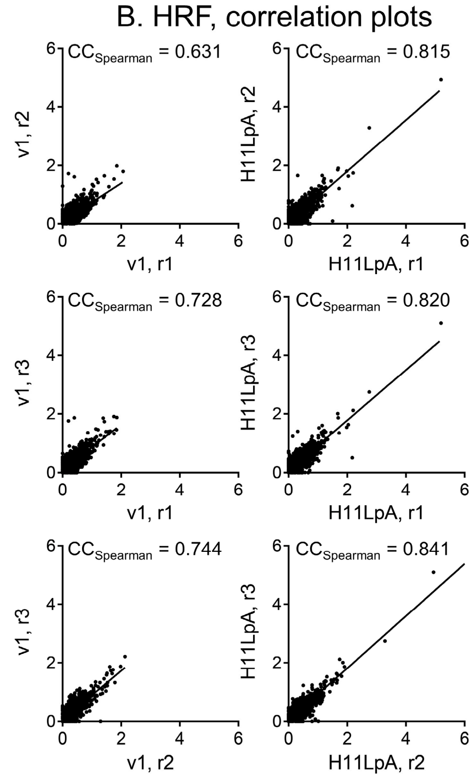

C. Comparison with other structured RNAs

	v1	HOTAIR	SRA	BRAVEHEART	COOLAIR	RepA
	this work	Somarowthu et al, 2015	Novikova et al, 2012	Xue et al, 2016	Hawkes et al, 2016	Liu et al, 2017
Size (nt)	1595	2148	873	590	658	1630
Stem loops	51	28[1]	31[1]	32[1]	29[1]	38[1]
Multiway junctions	16	13[1]	11[1]	3[1]	7[1]	11[1]
Internal loops	40	51[1]	53[1]	24[1]	39[1]	34[1]

[1]: proportional to the size of MEG3

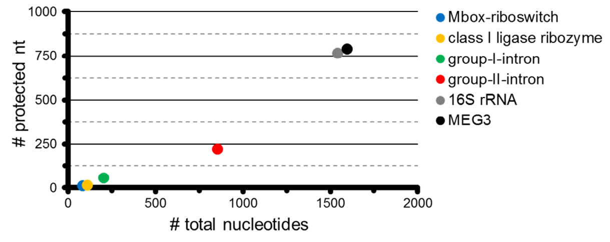

Fig S7: AFM analysis of v1 and H11LpA (Related to Fig. 6). **A)** Atomic force microscopy images of v1 and H11LpA. Top left: large view field of a sample of v1 in the compact (K^+ and Mg^{2+}) state, representative of the homogeneity of the sample on the mica support. Top right and bottom: Collection of 100 single particles of v1 in the compact (K^+ and Mg^{2+}, top right) and intermediate (K^+ only, bottom left) states, and of H11LpA in K^+ and Mg^{2+} (bottom right). The xy scale bar is indicated by a white line at the bottom of each panel. The z color scale bar for all AFM panels is indicated on the top right. **B)** SEC-SAXS of v1 in intermediate folding state (K^+ only), used for calculation of R_g and D_{max} (**Table S1**). Left: Scattering curve. Middle: Guinier plot. Right: P(r) plot. **C)** AUC titration of v1 at different magnesium concentrations, used for calculation of R_h, $C_{Mg1/2}$ and n_{Hill} (**Table S1**).

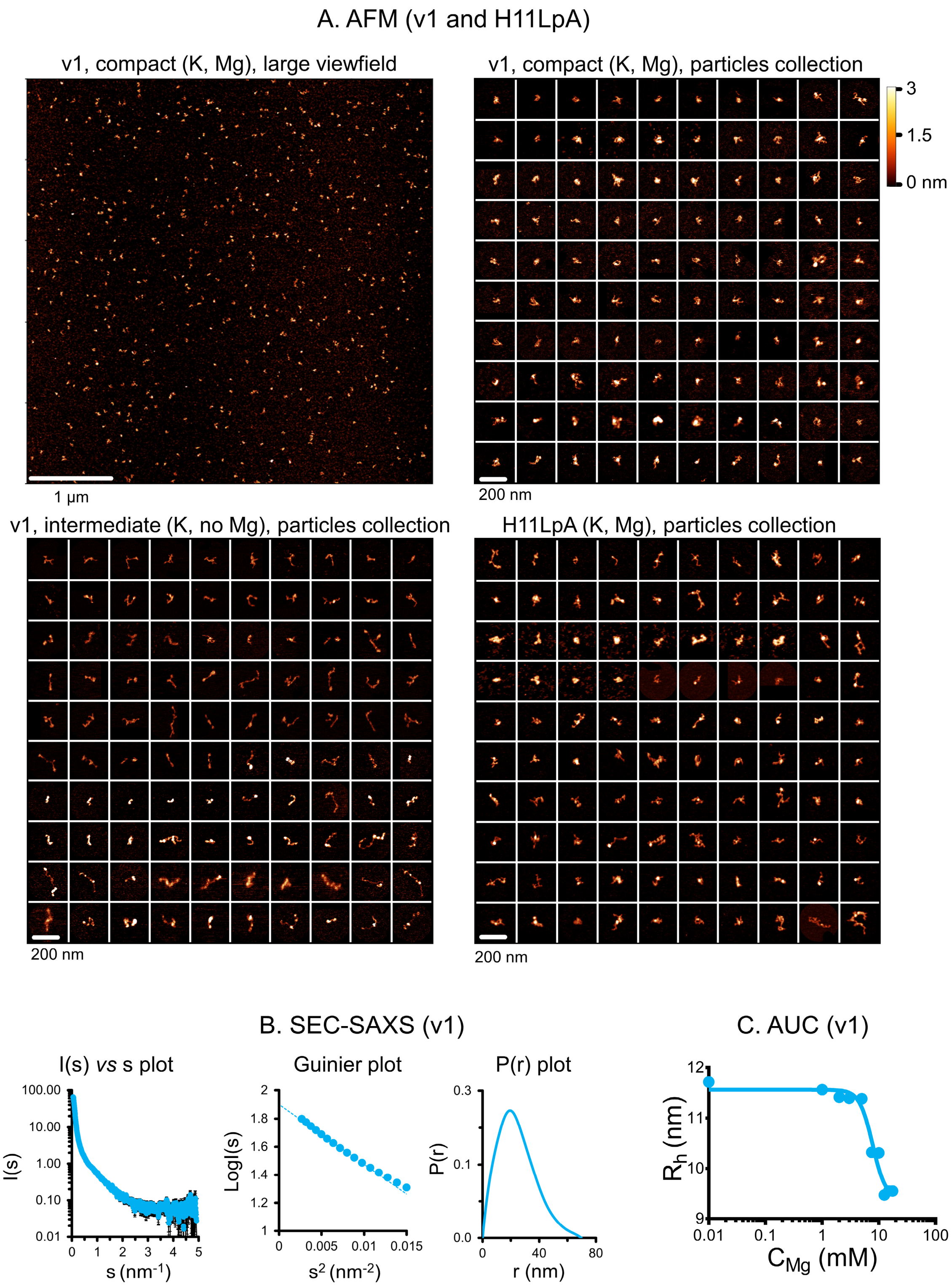

A. AFM (v1 and H11LpA)
v1, compact (K, Mg), large viewfield
1 µm
v1, compact (K, Mg), particles collection
200 nm
3
1.5
0 nm
v1, intermediate (K, no Mg), particles collection
200 nm
H11LpA (K, Mg), particles collection
200 nm
B. SEC-SAXS (v1)
I(s) vs s plot
I(s)
s (nm-1)
Guinier plot
LogI(s)
s2 (nm-2)
P(r) plot
P(r)
r (nm)
C. AUC (v1)
Rh (nm)
CMg (mM)

Supplemental Tables

Table S1: Hydrodynamic parameters of v1 (Related to Fig. 1, 5 and 6). R_g indicates the radius of gyration, R_h the hydrodynamic radius, D_{max} the maximum particle size obtained by SAXS, $C_{Mg1/2}$ the magnesium concentration at which v1 reaches half compaction, n_{Hill} the Hill coefficient of the fit to the AUC compaction curve, and D_{av} the average particle size obtained by AFM from PSD analyses.

Parameter	Technique	MEG3v1 state	Value
R_g	SEC-SAXS	intermediate (K^+ only)	18 nm
D_{max}	SEC-SAXS	intermediate (K^+ only)	70 nm
R_h	DLS	intermediate (K^+ only)	14 nm ± 7 nm
R_h	AUC	intermediate (K^+ only)	12 nm ± 0.1 nm
R_h	AUC	compact (10 mM Mg^{2+})	10 nm ± 0.1 nm
R_h	AUC	compact (50 mM Mg^{2+})	8.8 nm ± 0.3 nm
$C_{Mg1/2}$	AUC	Mg^{2+} titration (0-50 mM)	6.9 ± 0.35 mM
n_{Hill}	AUC	Mg^{2+} titration (0-50 mM)	3.9 ± 0.6
R_g / R_h		intermediate (K^+ only)	1.3 – 1.5
D_{av}	AFM	intermediate (K^+ only)	~85 nm
D_{av}	AFM	compact (10 mM Mg^{2+})	~65 nm

Table S2: Evolutionary conservation of the MEG3 exons (Related to Fig. 2, provided as a supplementary Excel file).

Table S3: Sequences of primers used in qRT-PCR (Related to Fig. 4).

Target	Primer name	Sequence (5'→3')
MEG3 D1 (Ex2)	13F_112_D1Ex2	GTCTCTCCTCAGGGATGAC
	14R_191_D1Ex2	TTGGCAGCAGCTCAGCA
MEG3 D2 (Ex3)	15F_230_D2Ex3	GAGCACGGTTTCCTGGAT
	Meg3RT22	CTGGCTGGTCAGTTCCGGTC
MEG3 D3 (Ex3)	01F_MEG3_Ex3	TCGATGAGAGCAACCTCCTA
	02R_MEG3_Ex3	TGCTGATCACCTCCTCTATG
MEG3 D4 (Ex5)	08F_951_Ex5	GGCCTGTCTACACTTGCTG
	05R_1029_D4	GGAGTAGAGCGAGTCAGGAA
MEG3 D5 (Ex10)	09F_1077_Ex10	GGCTGAAGAACTGCGGAT
	10R_1179_Ex10	AACCAGGAAGGAGACGAGAG
Neomycin	pcDNA3-F-Neo	TGGATTGCACGCAGGTTCT
	pcDNA3-R1-Neo	GGACAGGTCGGTCTTGACA
Actin	ACTB_Fwd	TTCCAGCAGATGTGGATCAG
	ACTB_Rev	GGTGTAACGCAACTAAGTCA
TP53 (p53)	03-F-cds-p53	CCTCAGCATCTTATCCGAGTG
	04-R-cds-p53	ACAGTCAGAGCCAACCTCA
BAX	BAX_Fwd	CACCAGCTCTGAGCAGATC
	BAX_Rev	GCTGCCACTCGGAAAAAG
CDKN1A (p21)	19-F-exon-p21	TGCCCAAGCTCTACCTTC
	20-R-exon-p21	GACAGTGACAGGTCCACAT
GADD45A	01F_GADD45	CCGACAATGTGACCTTCTG
	02R_GADD45	CGCACTATGTCGATGTCGTTC
GDF15	03F_GDF15	GCTACGAGGACCTGCTAAC
	04R_GDF15	CTTCTGGCGTGAGTATCCG
NOXA	NOXA_Fwd2	GGAAGTCGAGTGTGCTACTCAA
	NOXA_Rev2	CAGGTTCCTGAGCAGAAGAGTT

Table S4: Antibodies used for Western Blot analysis (Related to Fig. 4).

Antibody Specificity	Species and Isotype	Amount used	Catalog Number	Source
BAX (D2D)	Mouse IgG1	1:200	sc-20067	Santa Cruz
p21 (187)	Mouse IgG1	1:277	sc-817	Santa Cruz
p53 (DO-1)	Mouse IgG2a	1:200	sc-126	Santa Cruz
actin	Rabbit IgG	1:1000	ab1801	Abcam
mouse IgG1 Alexa Fluor 647	Goat IgG	1:1000	A-21240	Thermo
mouse IgG (H+L) Alexa Fluor 647	Goat IgG	1:1000	A-32728	Thermo
rabbit IgG Alexa Fluor 488	Goat IgG	1:1000	A-32731	Thermo

SUPPLEMENTAL FILES

Data File 1: Sequence alignments of v1 (Related to Fig. 2). The first alignment in the file, named "E3-clustal_19sequences.sto" reports the Clustal omega alignment of 19 seed sequences of E3 (~D2-D3); the second alignment in the file, named "E3-Infernal_41sequences.sto" reports the Infernal alignment of 41 sequences of E3 (~D2-D3); the third alignment in the file, named "E1E2-Clustal_33sequences.sto" reports the Clustal omega alignment of 33 sequences of E1-E2 (~D1); the fourth alignment in the file, named "E12-Clustal_40sequences.sto" reports the Clustal omega alignment of 40 sequences of E12.
