# Supplementary material for: Conserved Pseudoknots in lncRNA MEG3 Are Essential for Stimulation of the p53 Pathway
Source: Mol Cell. 2019 Sep 5;75(5):982–995.e9. doi: 10.1016/j.molcel.2019.07.025 (PMC6739425; doi:10.1016/j.molcel.2019.07.025)
Supplement: Document S2. Article plus Supplemental Information [file mmc4.pdf]

Molecular Cell

Article

Conserved Pseudoknots in lncRNA MEG3 Are Essential for Stimulation of the p53 Pathway

Graphical Abstract

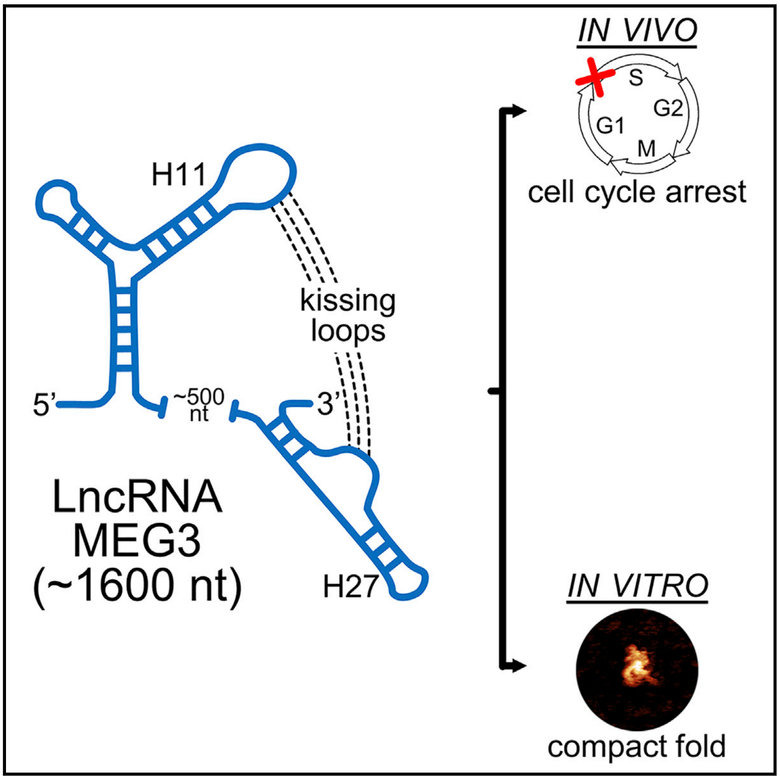

Highlights

- LncRNA MEG3 is a tumor suppressor that stimulates the p53 pathway
- The p53-stimulating core of MEG3 comprises of two conserved, structured domains
- Two distal motifs in the MEG3 core form pseudoknot interactions ("kissing loops")
- Mutations in these pseudoknots disrupt MEG3 architecture and impair its function

Authors

Tina Uroda, Eleni Anastasakou, Annalisa Rossi, ..., Alberto Inga, Isabel Chillón, Marco Marcia

Correspondence

ichillon@embl.fr (I.C.), mmarcia@embl.fr (M.M.)

In Brief

Uroda et al. identify the structural and functional core of the human lncRNA MEG3, revealing that point mutations designed to disrupt the MEG3 fold impair function. The work proves the importance of lncRNA tertiary structure motifs for selective and specific control of key cellular processes such as the p53 stress response.

Uroda et al., 2019, Molecular Cell *75*, 982–995
September 5, 2019 © 2019 The Authors. Published by Elsevier Inc.
https://doi.org/10.1016/j.molcel.2019.07.025

Molecular Cell
Article

Conserved Pseudoknots in lncRNA MEG3 Are Essential for Stimulation of the p53 Pathway

Tina Uroda,[1] Eleni Anastasakou,[1] Annalisa Rossi,[2] Jean-Marie Teulon,[3] Jean-Luc Pellequer,[3] Paolo Annibale,[4] Ombeline Pessey,[1] Alberto Inga,[2] Isabel Chillón,[1,*] and Marco Marcia[1,5,*]
[1]European Molecular Biology Laboratory (EMBL) Grenoble, 71 Avenue des Martyrs, Grenoble 38042, France
[2]Department CIBIO, University of Trento, via Sommarive 9, 38123 Povo (Trento), Italy
[3]Université Grenoble Alpes, CEA, CNRS, Institut de Biologie Structurale (IBS), 38000 Grenoble, France
[4]Max Delbruck Center, Robert-Rössle-Straße 10, 13092 Berlin, Germany
[5]Lead Contact
*Correspondence: ichillon@embl.fr (I.C.), mmarcia@embl.fr (M.M.)
https://doi.org/10.1016/j.molcel.2019.07.025

SUMMARY

Long non-coding RNAs (lncRNAs) are key regulatory molecules, but unlike with other RNAs, the direct link between their tertiary structure motifs and their function has proven elusive. Here we report structural and functional studies of human maternally expressed gene 3 (MEG3), a tumor suppressor lncRNA that modulates the p53 response. We found that, in an evolutionary conserved region of MEG3, two distal motifs interact by base complementarity to form alternative, mutually exclusive pseudoknot structures ("kissing loops"). Mutations that disrupt these interactions impair MEG3-dependent p53 stimulation *in vivo* and disrupt MEG3 folding *in vitro*. These findings provide mechanistic insights into regulation of the p53 pathway by MEG3 and reveal how conserved motifs of tertiary structure can regulate lncRNA biological function.

INTRODUCTION

Long non-coding RNA (lncRNA) structures are increasingly being recognized as important modulators of cellular processes, including chromatin remodeling, DNA repair, and translation (Mercer et al., 2009). Among the more than 32,000 human lncRNAs (Volders et al., 2013), a subgroup emerged as particularly suited for mechanistic studies based on their evolutionary conservation (Necsulea et al., 2014), specific cellular distribution (Cabili et al., 2015), tissue localization (Kaushik et al., 2013), and clinical relevance (Sauvageau et al., 2013; Wapinski and Chang, 2011). The secondary structures of a handful of such lncRNAs have been experimentally mapped, including SRA (Novikova et al., 2012b), HOTAIR (Somarowthu et al., 2015), XIST (Smola et al., 2016), RepA (Liu et al., 2017), roX (Ilik et al., 2013), BRAVEHEART (Xue et al., 2016), COOLAIR (Hawkes et al., 2016), NEAT1 (Lin et al., 2018), and parts of lincRNA-p21 (Chillón and Pyle, 2016). However, unlike with other RNAs, it has not yet been possible to systematically connect information regarding tertiary structure motifs of lncRNAs with their biological function, partly because the size and complexity of these molecules present unprecedented challenges for biophysical studies.

Human maternally expressed gene 3 (MEG3) is an alternatively spliced nuclear lncRNA abundant in the brain, placenta, and endocrine glands (Mondal et al., 2015; Zhang et al., 2003). MEG3 is expressed under the control of differentially methylated promoters from the Dlk1-MEG3 imprinted locus on chromosome 14q32, which also encodes other ncRNAs, none of which overlap with MEG3 exons (McMurray and Schmidt, 2012; Miyoshi et al., 2000). In embryonic cells, where it is not imprinted (McMurray and Schmidt, 2012), MEG3 silences genes involved in neurogenesis by regulating chromatin targeting of *Polycomb* proteins, and MEG3 expression is needed during neuronal development (Kaneko et al., 2014; Mercer et al., 2008; Mondal et al., 2015). Instead, in adult cells, where it becomes imprinted, MEG3 stimulates the p53 pathway, inducing cell cycle arrest and apoptosis (Zhou et al., 2007). In most human cancer cell lines and certain primary tumors, such as pituitary adenoma, MEG3 is downregulated via hypermethylation of the maternal allele, but its ectopic expression reduces tumor progression; thus, MEG3 acts as a tumor suppressor (Cheunsuchon et al., 2011; Zhou et al., 2012). Therefore, understanding the molecular mechanism of MEG3 is crucial to improve our knowledge of specific p53-related carcinogenic pathways.

In vitro and *in vivo* studies suggest that MEG3 interacts with p53 protein, leading to selective upregulation of p53 target genes (Zhou et al., 2007; Zhu et al., 2015). The 27 known splice variants of MEG3, which contain variable middle exons flanked by common exons at the 5′ (E1–E3) and 3′ (E10–E12) ends, vary in their ability to stimulate the p53 pathway (Zhang et al., 2010b). Changes in the MEG3 splicing pattern under stress lead to fluctuations in the p53 stress response (Zhang et al., 2010b). Interestingly, deletion mutagenesis of MEG3 impairs stimulation of the p53 pathway, suggesting that specific regions of this lncRNA are important for the p53 response (Zhang et al., 2010b; Zhou et al., 2007). However, the link between the structure of MEG3 and its functional effects on p53 remains to be defined.

To address this issue, we set out to characterize the secondary and tertiary structures of three MEG3 splice variants *in vitro*

 © 2019 The Authors. Published by Elsevier Inc.
This is an open access article under the CC BY license (http://creativecommons.org/licenses/by/4.0/).

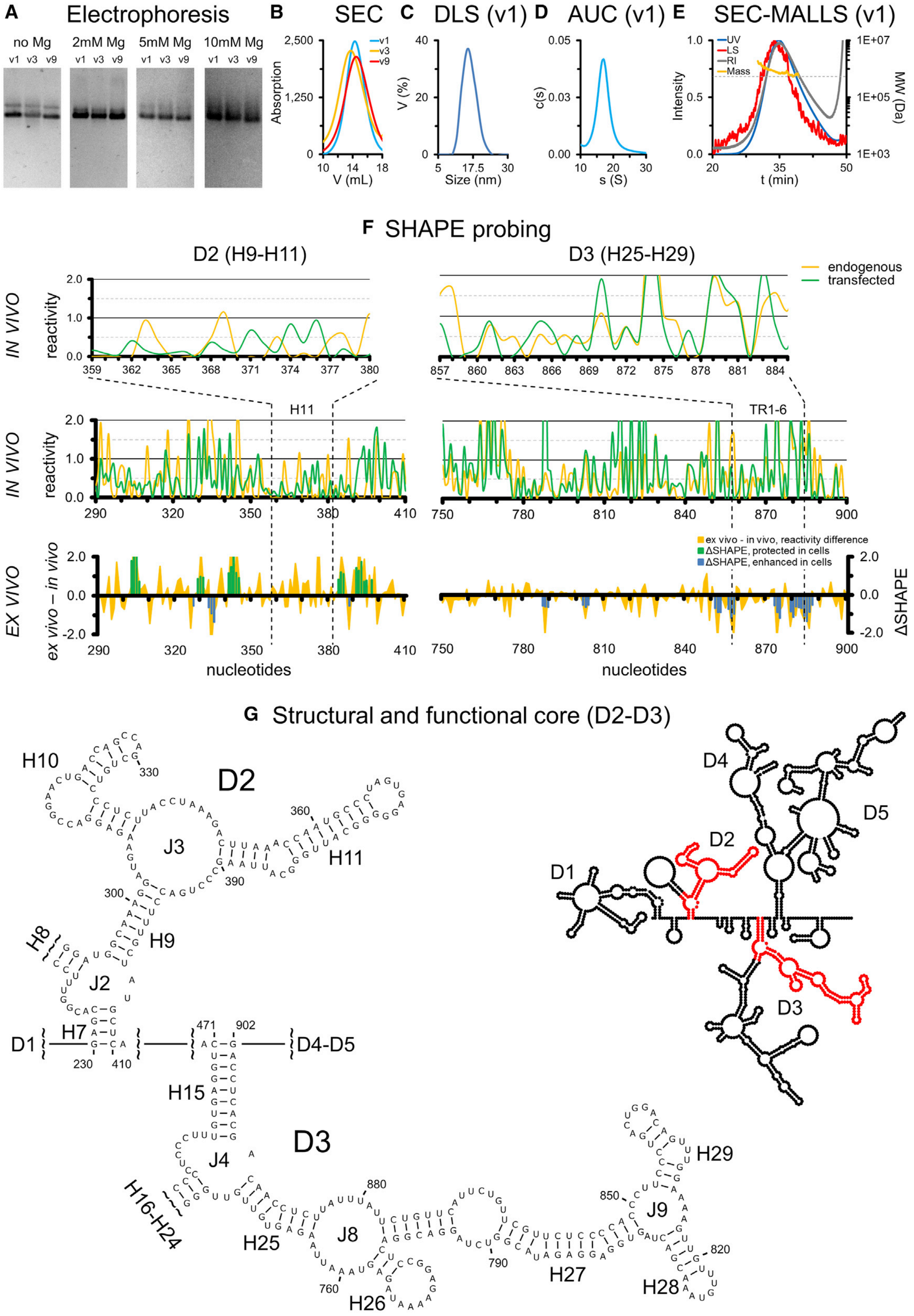

(legend on next page)

and *in vivo*. Guided by cell-based functional assays, we identified the functional core of MEG3. We found that two distal motifs complementary to each other in sequence are strictly required for stimulation of the p53 pathway. Point mutations designed to break the long-range base-pairing interactions between these motifs and to perturb the global 3D fold of MEG3 severely impair its ability to stimulate the p53 pathway. These findings underscore the importance of specific MEG3 tertiary structural elements for stimulation of the p53 pathway and provide mechanistic insights into this important tumor suppressor lncRNA.

RESULTS

Three Splicing Variants of Human MEG3 Share a Common Structural Core that Is Evolutionarily Conserved in Mammals

We expressed and purified the most abundant MEG3 splice variant (v1 or MEG3) as well as the two variants that induce the lowest (v3 or MEG3a) and the highest (v9 or MEG3e) degree of stimulation of the p53 pathway. After confirming homogeneity (Figures 1A–1E; Table S1), we mapped the secondary structures of these three variants by *in vitro* selective 2′-hydroxyl acylation analyzed by primer extension (SHAPE) using 3 reagents (1-methyl-7-nitroisatoic anhydride [1M7], 1-methyl-6-nitroisatoic anhydride [1M6], and N-methylisatoic anhydride [NMIA]; Figures 1G and S1–S3). We then used a fourth reagent (dimethyl sulfate [DMS]; Figure S1) to validate the map of v1, which we take as the reference isoform for structural description.

Variant v1, which spans 1,595 nt defined by common exons E1–E3 and E10–E12 and by varying E5, adopts a modular organization, including 5 structural domains (D1–D5) whose boundaries localize close to exon junctions, so that D2 (nt 230–410) and D3 (nt 471–902) together comprise E3 (Figures S1A, S2A, and S3). D2 and D3 are also preserved in v3, which starts 24 nt downstream of v1 and spans 1,712 nt defined by variable E6 along with E1–E3/E5/E10–E12 (Figures S1B, S2B, and S3), and in v9, which spans 1,481 nt defined by common E1–E3/E10–E12 and lacks any variable middle exon (Figures S1C, S2C, and S3). The most structurally stable portion of MEG3 (D2-D3; Figure 1G) folds reproducibly independent of the computational algorithm used to compute its secondary structure (Superfold, Siegfried et al., 2014; or RNA structure, Reuter and Mathews, 2010; Figure S4).

This motif also corresponds to the most evolutionarily conserved region of MEG3. For instance, Rfam family RF01872 covers a region in E3 (H21–H23) common to 53 putative MEG3 sequences from 40 mammals. Using the sequence of E3 and BLAT (Kent, 2002), we identified the complete E3 in 46 mammals, covering all mammalian orders except Monotremata (orders defined according to Tarver et al., 2016). MEG3 transcription has been confirmed and annotated in the NCBI for only 6 of these 46 species (human, orangutan, mouse, rat, cow, and pig). Besides E3, we also identified E1 and E2 in 33 species and E10–E12 in 19 species. We additionally identified E12 but not E10 or E11 in 21 species. We could not detect, or only partially detected E1 and E2 in Prosimians, Eulipotyphla, Xenarthra, Afrotheria, and Marsupialia, and we could not identify E10–E12 in Xenarthra and Marsupialia. Finally, we could not identify MEG3 beyond mammals (Table S2; Data S1). Because E3 is the most conserved region of MEG3, we aligned this region in Infernal, including the corresponding secondary structure information from v1 (Figure 2). The final alignment includes 41 mammalian MEG3 sequences and reveals that the most conserved region is the H11 stem-loop structure, in which 6 bp and the entire terminal loop are invariant and 3 additional bp co-vary.

In summary, our data suggest that, in human MEG3 exon E3, which comprises domains D2 and D3, represents the most conserved portion in sequence and secondary structure.

Systematic Cell-Based Assays Dissect the Functional Contribution of Each Structural Domain of MEG3

To assess the relationship between the MEG3 secondary structure and the function of this lncRNA as a tumor suppressor, we performed cell cycle (Lu et al., 2013) and luciferase reporter assays (Zhang et al., 2010a) in HCT116, a human cell line that expresses wild-type p53 and negligible levels of endogenous MEG3 (Figure S5N). Cell cycle assays showed that MEG3 induces arrest specifically at the G1/S but not the G2/M checkpoint and does not induce apoptosis in HCT116 (Figures 3A–3C, 3F–3H, S5A–S5C, and S5F–S5H). Cell cycle arrest by MEG3 is exclusively p53-dependent because we did not observe any MEG3 effect in HCT116-p53$^{-/-}$ cells (Figures 3I and S5I). Furthermore, luciferase reporter assays showed that MEG3 stimulates expression of p53 target genes. The MEG3 effect is dose-dependent and exclusively p53-dependent because luciferase production in HCT116-p53$^{-/-}$ cells is minimal and identical in cells transfected with MEG3 or a control vector (Figures 3J and S5J–S5M). Importantly, the intensity of the MEG3 effect depends on the p53 response element (p53RE) in the promoter of the luciferase reporter gene (Figure 3K). We identified two reporters on which all three MEG3 variants are active: one possessing an optimized p53RE used in previous MEG3 studies (p53Luc; Zhang et al., 2010a) and another possessing the p53RE of the endogenous MDM2 gene (pGL-MDM2; Menendez et al., 2010).

Using the luciferase assay and the p53Luc reporter, we systematically probed the structure of v1 (Figures 4A–4D). We found that none of the v1 domains in isolation can activate p53 and that

Figure 1. The MEG3 D2-D3 Structural Core

(A and B) Native agarose gel electrophoresis (A) and size exclusion chromatography (SEC; B) of v1, v3, and v9.

(C–E) Dynamic light scattering (DLS; C), analytical ultracentrifugation (AUC; D), and SEC coupled to multi-angle laser light scattering (MALLS; E) profiles of v1.

(F) SHAPE reactivity values of individual nucleotides in D2 and D3 (H11 and TRs motifs are delimited by the dotted vertical lines). Bottom: difference between *ex vivo* minus *in vivo* reactivity values and deltaSHAPE values (endogenous MEG3 datasets). Center: *in vivo* 1M7 reactivity values in endogenous MEG3 and transfected v1. Top: magnification of *in vivo* 1M7 reactivity values for H11 and the TRs. Structure maps and complete data from *in vitro* probing are reported in Figures S1–S4.

(G) Structure of selected motifs in the D2-D3 core (D indicates domains, H helices, and J junctions). Inset: schematic of the complete v1 structure (from Figure S1), with the core shown in red.

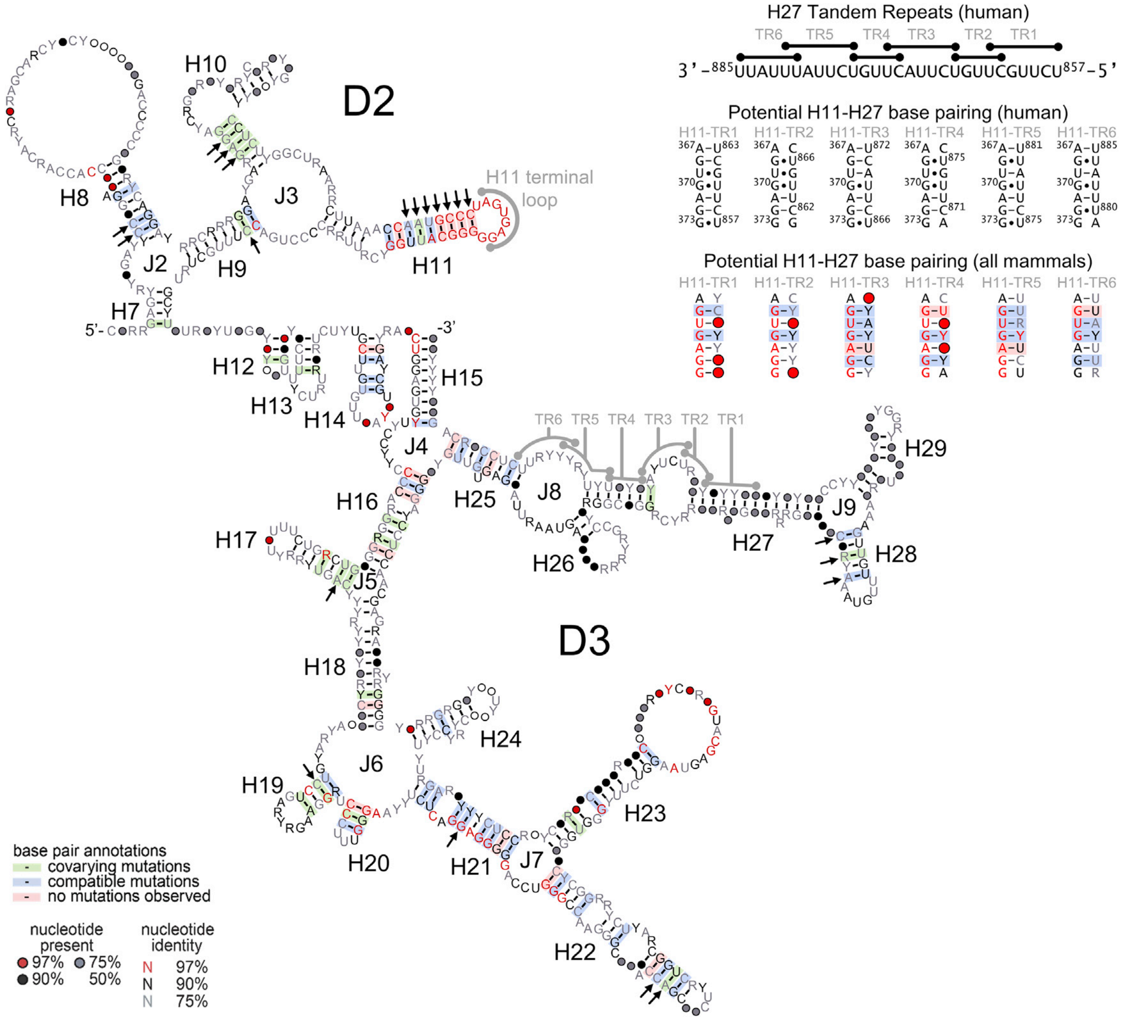

Figure 2. Evolutionary Conservation in the MEG3 Core

R2R plot of 41 D2-D3 (E3) sequences aligned in Infernal (color legend at the bottom left; Weinberg and Breaker, 2011). Arrows indicate covariant base pairs of potential statistical significance (see STAR Methods for details). Human sequences of the H27 TRs, corresponding base-pairing to the H11 terminal loop, and potential covariation of the base-pairing interaction are shown at the top right.

D1/D4/D5 are dispensable for stimulation of the p53 pathway. In contrast, deletion of either D2 or D3 (constructs ΔD2 and ΔD3) abolishes stimulation of the p53 pathway. Curiously, deletion of D4 or D5 enhances v1 activity. The same stimulation/inhibition pattern can be observed for individual exons. Deleting E5 (as in v9), E10, E11, or E12 stimulates the p53 pathway, but neither E1 or E2 nor E10–E12 in isolation can stimulate the p53 pathway. The only exon that can stimulate the p53 pathway in isolation is E3, which covers D2-D3. Interestingly, D2, which is inactive in isolation, can partially stimulate the p53 pathway when co-transfected with ΔD2, which is also inactive *per se*, suggesting that D2 and D3 functionally cooperate when supplied either in *cis* or in *trans* (Figure 4A).

Considering that D2-D3 is also the most conserved portion of MEG3, we systematically focused on this region for detailed functional probing. We found that deleting H8 (D2) or H17, H28, and H21–H23 (D3) does not have a significant effect on activity, but deletion of H11 (D2) or H25–H29 (D3) dramatically reduces stimulation of the p53 pathway (Figure 4B). Remarkably, within H11, disrupting the helical stem abolishes p53 stimulation, which can be recovered to near wild-type levels by compensatory mutations, suggesting that the structure of this element is essential for function (Figures 4C and 4D). Furthermore, deleting the H11 terminal loop (nt 366–373 in v1) or mutating all of its residues to adenosines (mutant H11LpA) impairs stimulation of the p53 pathway. Most strikingly, nearly all point mutations at positions 368–372 in the H11 terminal loop (GUGAG motif) also abolish stimulation of the p53 pathway (Figure 4C).

We thus focused on the H11LpA and $G^{370}C$ mutants to analyze the effects of H11 mutations on p53 regulation. First, we found that H11LpA and $G^{370}C$ reduce luciferase expression not only from the p53Luc but also from the pGL-MDM2 promoter (~35% residual activity with respect to v1), and, most importantly, both mutants failed to arrest the cell cycle (Figures 3D and 3E). Second, we

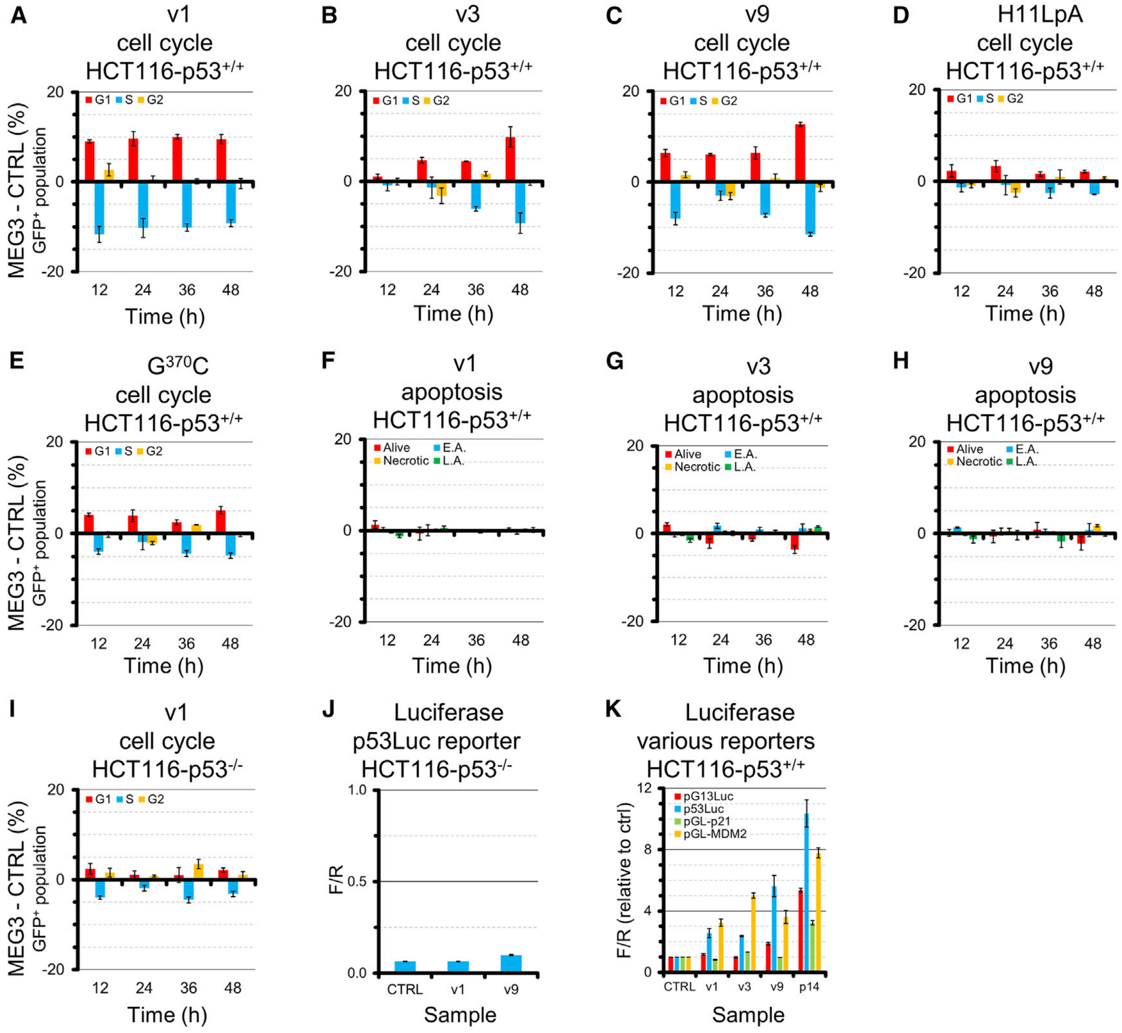

Figure 3. Selective Stimulation of the p53 Pathway by MEG3 Variants

(A–I) Cell cycle and apoptosis analysis of v1 (A), v3 (B), v9 (C), H11LpA (D), and $G^{370}C$ (E) in HCT116-$p53^{+/+}$, apoptosis analysis of v1 (F), v3 (G), v9 (H) in HCT116-$p53^{+/+}$, and cell cycle analysis of v1 in isogenic $p53^{-/-}$ cell lines (I). E.A., early apoptotic; L.A., late apoptotic.

(J) Luciferase assay performed in HCT116-$p53^{-/-}$ cells (absolute ratio of firefly luciferase versus *Renilla* luciferase chemiluminescence).

(K) Stimulation of the p53 pathway by v1, v3, v9, and $p14^{ARF}$ on 4 reporter vectors possessing different p53REs (pG13Luc, p53Luc, pGL-p21, and pGL-MDM2). Data were normalized to the signal of corresponding empty vectors. For this experiment, 500 ng of MEG3 vectors and 50 ng of $p14^{ARF}$ vectors were used for transfection in 12-well plates.

Error bars indicate SEM of 3 experiments.

established, by qRT-PCR and western blot, that v1 induces p53 expression, as reported previously (Zhou et al., 2007), but the H11LpA and $G^{370}C$ mutants do not (Figure 4E). Finally, by qRT-PCR and western blot, we revealed that v1 induces expression of endogenous p53 target genes, in line with previous reports (Zhou et al., 2007), but the H11LpA and $G^{370}C$ mutants do not, as expected from our luciferase reporter data (Figure 4F).

In summary, the p53-stimulating core of MEG3 is formed by D2 and D3 and specifically involves the two structural motifs H11 (D2) and H25–H29 (D3). Both the structure of the highly conserved H11 stem and individual nucleotides in the invariant H11 terminal loop are essential for stimulation of the p53 pathway and for cell cycle regulation.

The Functional and Evolutionarily Invariant H11 Stem-Loop Structure Is Not a Protein-Binding Site

Because the effects of MEG3 on p53 and its target genes have been proposed to depend on a direct MEG3-p53 interaction (Zhou et al., 2007; Zhu et al., 2015), we performed RNA immunoprecipitation (RIP) and pull-down experiments to compare v1 with the H11LpA mutant and examine whether the functional importance of H11 is related to protein binding, as reported previously for other lncRNA structural motifs (Ilik et al., 2013; Lu et al., 2016; Somarowthu et al., 2015; Xue et al., 2016). By RIP, we found that p53 interacts to a similar extent with both v1 and H11LpA and comparably to lncRNA DINO (Schmitt et al., 2016; Figure 4G). Analogously, by pull-down using biotinylated RNA, we could

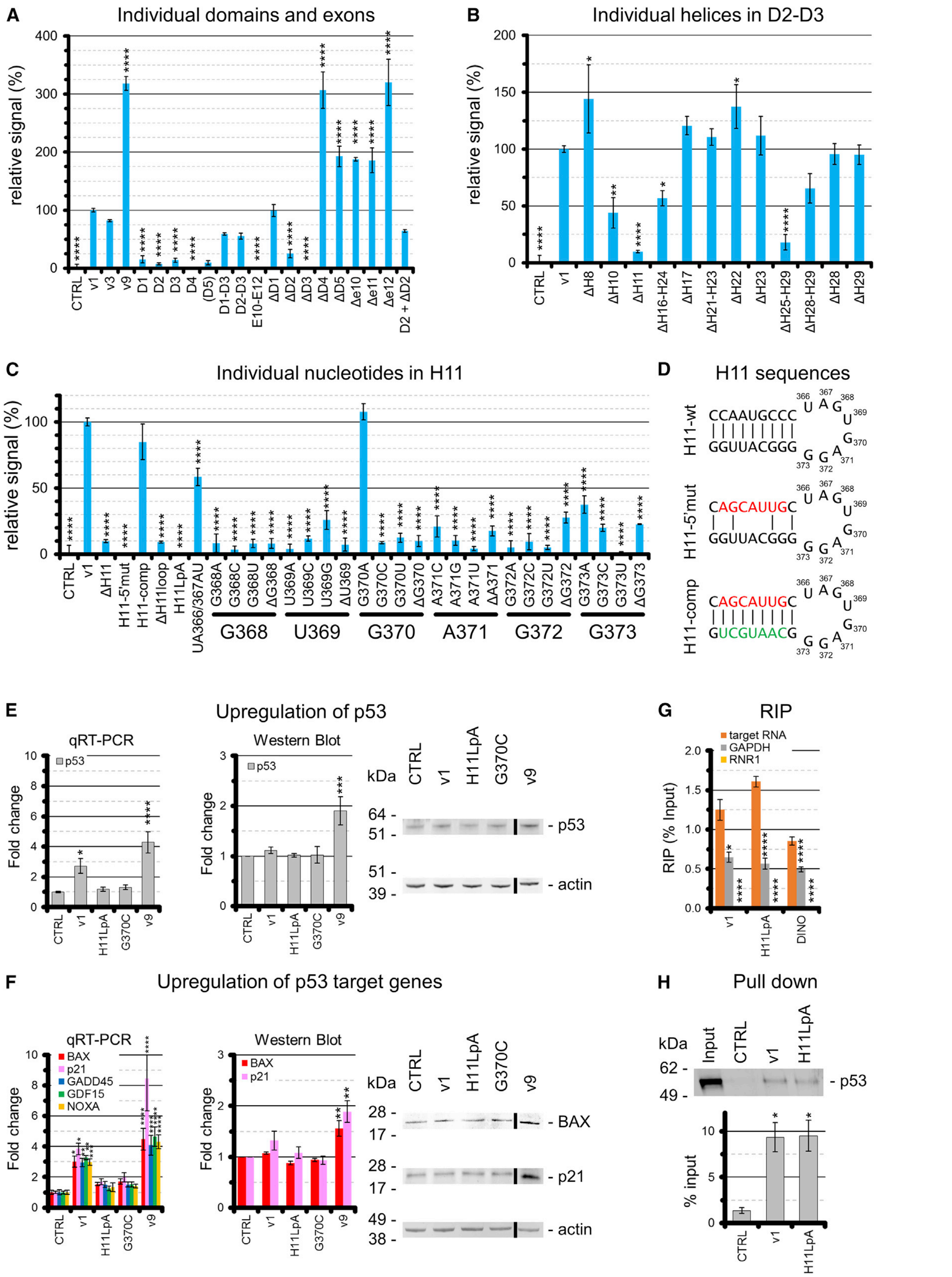

(legend on next page)

detect similar levels of p53 bound to v1 and to H11LpA (Figure 4H). Thus, we conclude that H11 is not involved in p53 binding.

We subsequently analyzed whether H11 is involved in other intermolecular interactions in the cell. To this end, we performed *in vivo/ex vivo* chemical probing analysis. These techniques both compare the chemical reactivity of MEG3 folded in the cellular environment, either in the presence of protein partners (*in vivo*) or after gentle extraction and protein removal under experimental conditions that are not expected to denature the RNA secondary structure (*ex vivo*) (Smola et al., 2015a; Figure 1F). To overcome the challenge posed for cellular probing by the complex alternative splicing pattern of MEG3, in our study we used WI38 fibroblasts to probe endogenous MEG3 (expression levels are reported in Figure S5N; expressed variants are v1 [68%] and v9 [26%]; Zhang et al., 2010b) and HCT116 cells to probe transfected v1 (expression levels of endogenous MEG3 in this cell line are negligible; Figure S5N). *In vivo* SHAPE reactivity values of individual nucleotides in the MEG3 core (D2-D3) in endogenous and transfected samples are similar (Figure 1F), suggesting that this region folds reproducibly in different cell lines. This observation reinforces the notion that D2-D3 is an important structural element of MEG3 and corroborates the use of the transfected system as a proxy to mimic near-physiological conditions for our functional assays. Surprisingly, within the H11 motif, we could not detect any significant reactivity difference between *ex vivo* and *in vivo* conditions (deltaSHAPE), suggesting that nucleotides in this region are unlikely to be involved in protein binding (Figure 1F).

In summary, our data reveal that non-functional H11 mutants preserve affinity for p53. More broadly, our *in vivo/ex vivo* SHAPE probing establishes that the H11 stem-loop structure is not a protein binding site, despite its high degree of sequence and structural conservation.

H11 Forms Conserved and Functional Pseudoknot Structures ("Kissing Loops") with H27

Seeking a molecular explanation for the functional importance of H11, we noticed that its terminal loop (residues 368–372, GUGAG motif) is chemically unreactive *in vitro* in all of our SHAPE maps despite being single stranded (Figures S1 and S3). However, by performing SHAPE on the H11LpA mutant, we noticed that this motif becomes highly reactive, whereas the reactivity of all other structural regions except H25–H29 remains nearly unchanged (Figure 5A). Considering these differences in *in vitro* SHAPE reactivity and the fact that, in the cell, D2 can stimulate the p53 pathway in *trans* with ΔD2 (Figure 4A), we hypothesized that MEG3 may adopt a higher-order structure in which H11 is constrained by intramolecular long-range tertiary interactions.

To address this hypothesis, we first analyzed the folding behavior of v1 and H11LpA in solution by analytical ultracentrifugation (AUC). Strikingly, these two constructs, which differ by only 6 of 1,595 nt, display the same hydration radius (R_h) at Mg^{2+} concentrations below the $C_{Mg1/2}$, but above the $C_{Mg1/2}$, the mutant compacts up to 8% less than the wild type (Figure 5B).

Having established by AUC that MEG3 folding *in vitro* is specific, we performed hydroxyl radical footprinting (HRF). We found that the D2-D3 core of v1 displays a solvent accessibility pattern typical of highly structured RNAs, with solvent-protected regions flanked by highly solvent-exposed residues (Figures 5C, S6A, and S6B). The number of solvent-protected nucleotides in MEG3 is proportional to that of other highly structured large RNAs (Figure S6C). These observations suggest that MEG3 folds in a specific conformation *in vitro*. Moreover, HRF analysis of H11LpA revealed that both the GUGAG motif and the H25–H29 motif, which display increased SHAPE reactivity (see above and Figure 5A), also become significantly more solvent-exposed than in v1 (Figure 5D).

Curiously, nt 857–885 in H25–H29 (3′ side of H27) comprise a conspicuous series of 6 tandem repeats (TR1–TR6) with sequences complementary to the GUGAG motif in H11, suggesting that H11 and H27 could base pair with each other to form long-range pseudoknot structures (kissing loops; Figure 2). Importantly, the potential to form H11–H27 pseudoknots is conserved in evolution because all mammalian sequences where we could identify MEG3 possess at least three such tandem repeats (Figure 2; Table S2). To test whether the putative H11–H27 pseudoknots actually form *in vivo* and are functionally relevant, we used the functionally impaired $G^{370}C$ mutant and introduced a

Figure 4. Functional Importance of MEG3 Structural Motifs

(A–C) p53-dependent luciferase assays using the p53Luc reporter on MEG3 variants, individual exons, and domains (A); on D2-D3 mutants (B); and on selected H11 mutants (C). Construct D5 is indicated in parenthesis because it is more than 10,000-fold less expressed than v1. Expression levels of all other constructs are reported in Figure S5O. Error bars indicate SEM of at least 3 experiments. Asterisks indicate a significant difference in relative luciferase signal with respect to v1 based on one-way ANOVA statistical tests in GraphPad (*p ≤ 0.05, **p ≤ 0.01, ***p ≤ 0.001, and ****p ≤ 0.0001).

(D) Sequences used to disrupt the H11 stem (H11-5′mut, red nucleotides) and corresponding compensatory mutations (H11-comp, green nucleotides).

(E) qRT-PCR and western blot analysis of p53 upregulation by v1, v9, H11LpA, and $G^{370}C$.

(F) qRT-PCR analysis measuring upregulation of p53 target genes (BAX, p21, GADD45A, GDF15, and NOXA) by v1, v9, H11LpA, and $G^{370}C$. Representative western blots for BAX and p21 are reported on the right (the endogenous signal for GADD45A, GDF15, and NOXA is too low in our system for accurate quantification).

(E and F) Error bars indicate SEM of 2 biological replicas, each performed in technical triplicates, and the black line indicates that the right and left parts of the images were manually joined because they were separated from each other in the raw image of the blot. Asterisks indicate significant variation with respect to control (CTRL) based on one-way ANOVA statistical tests in GraphPad (*p ≤ 0.05, **p ≤ 0.01, ***p ≤ 0.001, and ****p ≤ 0.0001).

(G) RNA immunoprecipitation using the DO1 anti-p53 antibody for cells transfected with the indicated constructs. Control samples using unspecific immunoglobulin G (IgG) produced negligible amplification and are not plotted. Values are reported as percent input. Error bars indicate SEM of 4 biological replicas, each performed in technical duplicates. Asterisks indicate significant variations in the amounts of immunoprecipitated control RNAs (GAPDH and RNR1) with respect to the target RNA (v1, H11LpA, or DINO) based on unpaired parametric t tests in GraphPad (*p ≤ 0.05, **p ≤ 0.01, ***p ≤ 0.001, and ****p ≤ 0.0001).

(H) Pull-down of p53 (detected by western blot) using *in vitro*-transcribed and biotinylated v1 and H11LpA and non-biotinylated v1 (CTRL). Values are reported as percent of input. Error bars indicate SEM of 3 biological replicas. Asterisks indicate significant variation of p53 pulled down by v1 and H11LpA with respect to CTRL based on one-way ANOVA statistical tests in GraphPad (*p ≤ 0.05, **p ≤ 0.01, ***p ≤ 0.001, and ****p ≤ 0.0001).

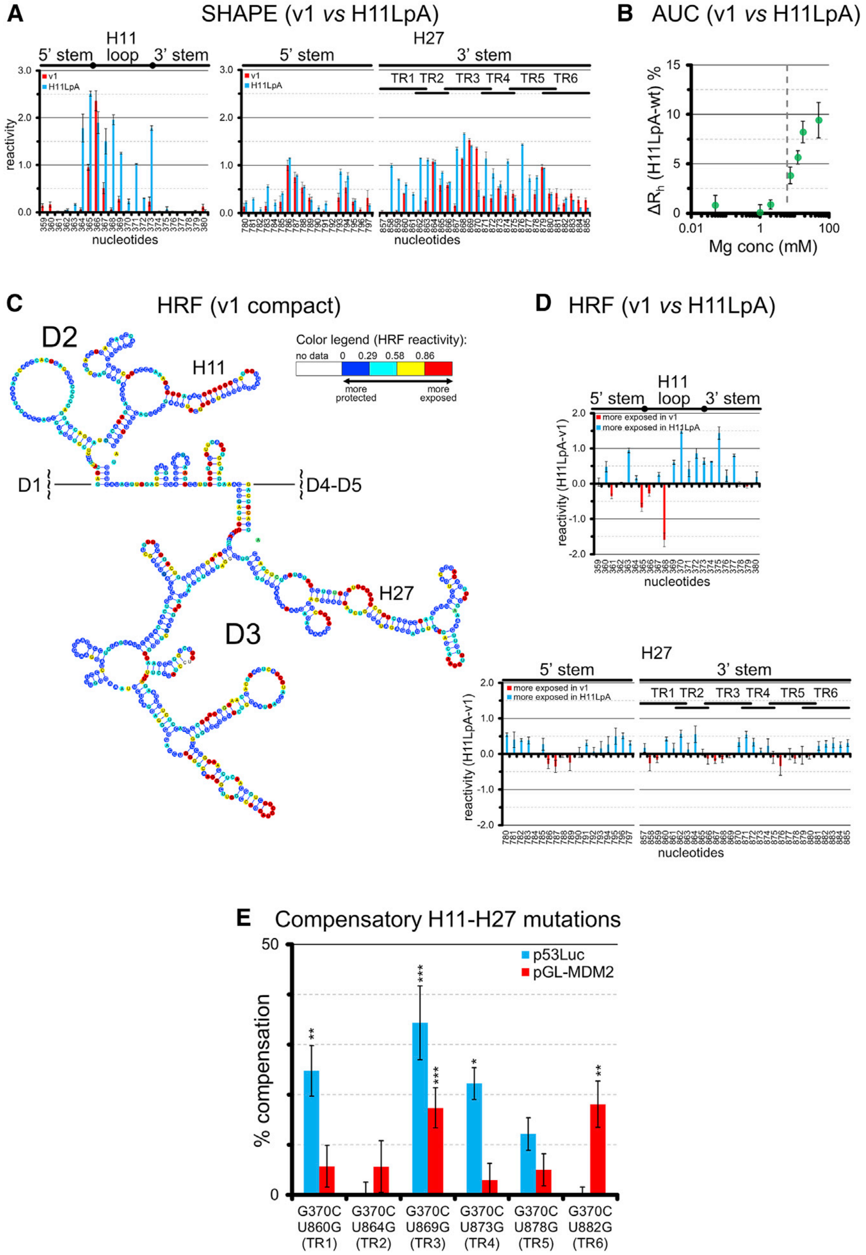

Figure 5. Structural and Functional Interconnections between H11 and H27

(A) SHAPE (1M7) reactivity values of individual nucleotides in H11 and H27 from compact v1 (from Figures S1–S3) and H11LpA.

(B) Difference in hydration radius (ΔR_h) between v1 and H11LpA at increasing Mg^{2+} concentrations, as measured by AUC (the vertical dotted line indicates the $C_{Mg1/2}$ of v1 from Table S1).

(C) Secondary structure map of compact v1 color-coded according to the HRF reactivity values of individual nucleotides. HRF analysis and normalization procedures are described in the STAR Methods.

(D) HRF reactivity values of individual nucleotides in H11 and H27 from compact v1 (from C) and H11LpA in K^+ and Mg^{2+}. Reactivity values of H11LpA were normalized to the reactivity values of v1 following the scaling procedure of QuSHAPE, as described previously (Karabiber et al., 2013). Raw reactivity plots and correlations between replicas are reported in Figure S6.

(E) p53-dependent luciferase assay (p53Luc and pGL-MDM2 reporters) on $G^{370}C$ and compensatory double mutants.

Error bars in (A) and (C)–(E) indicate SEM of 3 experiments. Asterisks indicate significant variation in relative luciferase signal with respect to the $G^{370}C$ single mutant (Figure 4C) based on one-way ANOVA statistical tests in GraphPad (*p $\leq$ 0.05, **p $\leq$ 0.01, ***p $\leq$ 0.001, and ****p $\leq$ 0.0001).

compensatory point mutation within each H27 TR. We tested all six resulting double mutants by luciferase assay using both the p53Luc and the pGL-MDM2 reporter. Strikingly, all double mutants except $G^{370}C/U^{864}G$ (H11/TR2) partially rescued activity on at least one reporter. Interestingly, the extent of rescue depends on the TR concerned: $G^{370}C/U^{869}G$ (H11/TR3) was the most efficient compensatory mutant on both p53Luc and the pGL-MDM2 reporters, $G^{370}C/U^{873}G$ (H11/TR4) rescued activity only on p53Luc but not on pGL-MDM2, and $G^{370}C/U^{882}G$ (H11/TR6) rescued activity only on pGL-MDM2 but not on p53Luc (Figure 5E).

Taken together, our data show that H11 and H27 are structurally connected *in vitro* because mutations on H11 affect the secondary and tertiary structures of H27 (Figures 5A and 5D). Moreover, *in vivo*, H11 and H27 are both required for stimulation of the p53 pathway (Figure 4B) and form functionally important base-pairing interactions because inactive H11 mutants can be rescued by compensatory H27 mutations (Figure 5E).

The Functional H11–H27 Pseudoknots Are Required for Compaction of MEG3 *In Vitro*

Having established that MEG3 compacts in a specific manner *in vitro* and that it forms a functionally important pseudoknot *in vitro* and *in vivo*, we attempted to visualize this lncRNA by single-particle 3D imaging. For our study, we used atomic force microscopy (AFM), a technique used previously to image other multi-domain structured RNAs and to monitor RNA conformational changes (García-Sacristán et al., 2015; Giro et al., 2004; Hansma et al., 1996; Lyubchenko et al., 2011; Schön, 2016; Yu et al., 2015).

We visualized v1 in three different folding states (Figures 6 and S7A; Table S1). In the presence of formamide, which denatures RNA, v1 forms elongated unstructured filaments, as expected. In the presence of K^+ ions, which induce RNA to form secondary but not tertiary structure motifs, v1 particles become shorter and taller, forming globular domains connected by flexible linkers.

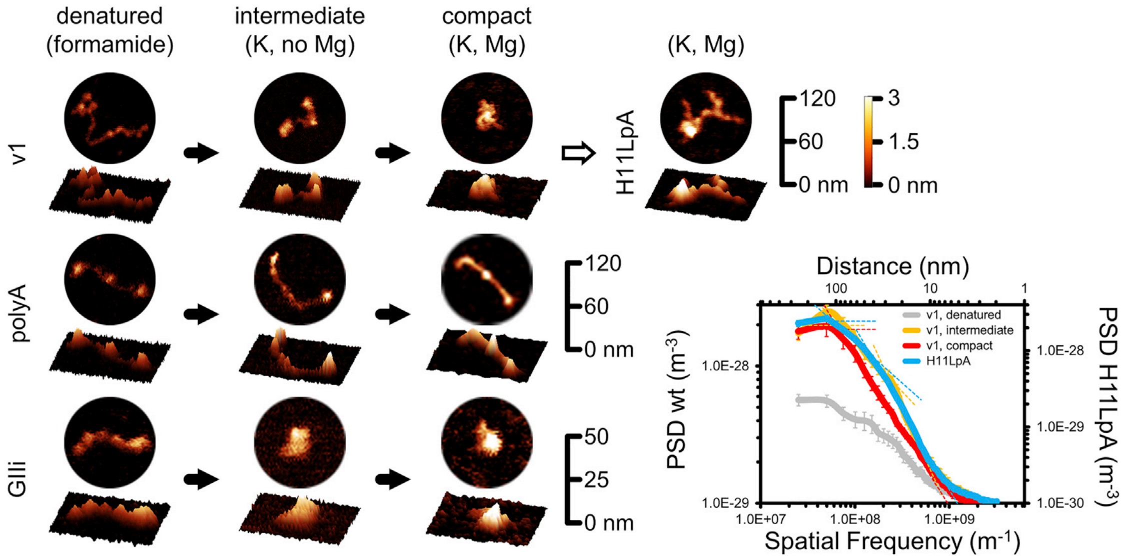

Figure 6. *In Vitro* Single-Particle Analysis of v1

Shown are representative AFM particles of v1, poly(A) RNA, and group II intron (GIIi) in formamide (denatured state), K^+ (intermediate state), and K^+ and Mg^{2+} (compact state). A representative particle of H11LpA in K^+ and Mg^{2+} is reported at the top right. Each particle is displayed in 2D and 3D representations. The xy scale bars are on the right for each molecule, and the z color scale bar is common to all samples. The bottom right panel shows PSD plots from images acquired at 1,024 × 1,024 $pixel^2$ with a pixel size of 0.98 nm/pixel for v1 in the denatured, intermediate, and compact states and of H11LpA in K^+ and Mg^{2+}. We obtained similar PSD plots for images acquired at 512 × 512 $pixel^2$ with a pixel size of 1.96 nm/pixel. Intercepts between linear fits to autoaffine regions in the spectra (dashed lines) indicate characteristic spatial frequencies. Error bars indicate SEM. We imaged ~100–110 particles in total per condition (see STAR Methods for details). The AFM processing pipeline and raw scan are reported in Figure S7.

Power spectral density (PSD) analysis, which provides an overview of the spatial features present in images (Calò et al., 2009; Higuchi, 1988), reveals two characteristic peaks. One peak likely corresponds to the globular domains of v1 (average size of 30 nm) and the second peak to the entire v1 (~85 nm), in line with dimensions measured in solution by small-angle X-ray scattering (SAXS; maximum particle size [D_{max}], ~70 nm; Figure S7B; Table S1). Finally, in the presence of K^+ and Mg^{2+}, which induce RNA tertiary folding (Su et al., 2005; Wadley et al., 2007; Woodson, 2005, 2010), v1 particles become even shorter and taller than with K^+ and lose their multi-domain organization to adopt an overall compact shape. Correspondingly, the PSD spectrum becomes steeper and loses the 30-nm shoulder. The intercept with the low-frequency plateau is now at ~65 nm, suggesting a degree of compaction (~25%) similar to that observed in solution by AUC (~20%; Figure S7C; Table S1). The folding behavior observed for v1 differs from that of low-complexity RNAs, such as poly(A) RNA homopolymers, and is similar to that of highly structured RNAs, like the *Oceanobacillus iheyensis* group II intron (Figure 6). Furthermore, we also imaged the H11LpA mutant in K^+ and Mg^{2+}. This mutant displays a dramatic defect in compaction. PSD analysis shows emergence of an inflection point at ~40 nm in the mutant, more closely resembling the intermediate than the compact state of v1 (Figures 6 and S7A).

In summary, our AFM analysis, supported by our highly correlated biochemical, biophysical, and functional characterization *in vitro* and *in vivo*, reveals that MEG3 folding is dictated by the formation of evolutionarily conserved and functionally important pseudoknots between H11 and H27.

DISCUSSION

Alternative Splicing Dictates Structural Organization in lncRNA MEG3

In this work, we characterized the secondary and tertiary structures of MEG3, a human lncRNA that functions as a tumor suppressor by stimulating the p53 pathway.

Our secondary structure maps of three MEG3 splice variants show that MEG3 forms highly structured domains, displaying Shannon entropy and SHAPE reactivity values comparable with highly structured RNAs (Mathews, 2004; Figures 1G, S1–S4, and S6C). The boundaries between structural domains define exon E3 as an independent module comprising domains D2 and D3 (Figures S1–S4). Correspondence between exon junctions and domain boundaries have been observed previously for another lncRNA, BRAVEHEART (Xue et al., 2016), but not for HOTAIR (Somarowthu et al., 2015) or XIST (Smola et al., 2016). For MEG3, which possesses at least 27 splice variants exhibiting different levels of p53 stimulation, such correspondence is remarkable and provides support for the previously proposed correlation between MEG3 exonic organization, structural architecture, and ability to stimulate the p53 pathway (Zhang et al., 2010b). D2 and D3 likely adopt a similar secondary structure across human MEG3 splicing variants (Figure S1) because they have similar chemical reactivity in different cell lines (endogenous and transfected; Figure 1F) and a distinct solvent protection pattern *in vitro* (Figure 5C). Importantly, we could identify D2-D3 (E3) in early-diverging mammals such as Marsupialia (i.e., Tasmanian devil; Data S1; Table S2),

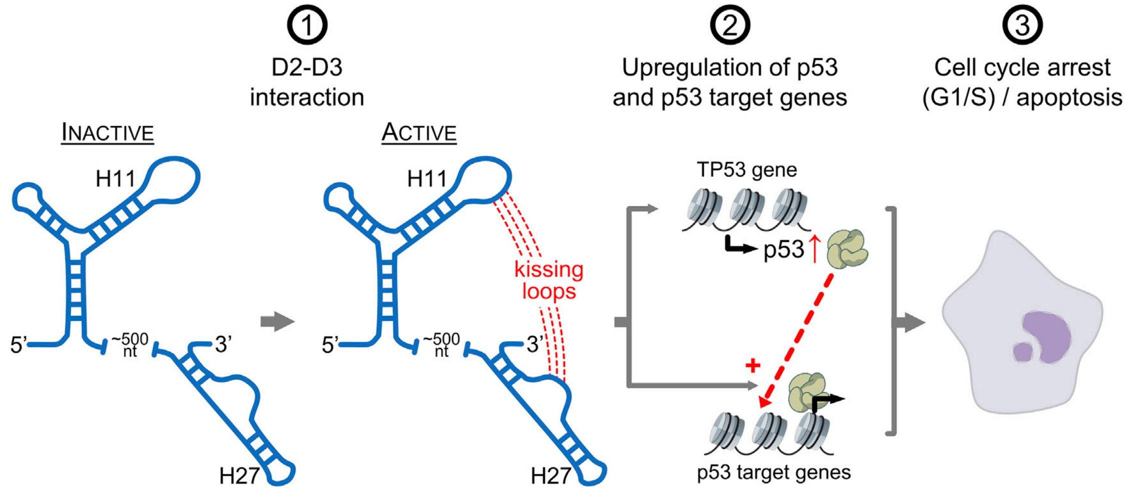

Figure 7. Model for MEG3-Dependent Stimulation of the p53 Pathway
A long-range interaction (kissing loops) between the H11 and H27 motifs is necessary to activate lncRNA MEG3. Active MEG3 upregulates p53 and p53 target genes. The resulting effect of such stimulation of the p53 pathway is cell cycle arrest at the G1/S checkpoint and/or apoptosis, depending on the cell type (a sketch of an apoptotic cell is shown on the right).

suggesting that this lncRNA originated at least 200 million years ago, subsequent to the gene duplication event involving the p53/p63/p73 ancestor that gave rise to p53 ~400 million years ago (Belyi et al., 2010). It is possible that, in early-diverging mammals (Marsupialia, Afrotheria, and Xenarthra) that seem to lack E1, E2, and E10–E12, the MEG3 sequence is too divergent to be identified with current algorithms. Alternatively, in those organisms, MEG3 may have actually been composed of E3 only, and other exons may have been acquired later in evolution to confer further specificity and/or additional functional roles to MEG3.

Conserved Nucleotides and Structured Motifs in the MEG3 Core Are Essential for Stimulation of the p53 Pathway

Our work also dissects the functional contribution of each structural domain of MEG3 to an exquisite and unprecedented level of detail. Although previous studies have identified specific functional motifs in lncRNAs, such as the asymmetric G-rich internal loop (AGIL) of BRAVEHEART that mediates cardiac specification in mice (Xue et al., 2016) or repeat stem-loop structures of roX that mediate dosage compensation in *Drosophila* (Ilik et al., 2013), the complexity of phenotypic assays has so far prevented systematic functional probing of lncRNA secondary structures. In our work, we unearthed not only the functional importance of macroscopic structural motifs (i.e., entire domains or stem-loop structures) but also of individual nucleotides in H11 (D2) and H27 (D3), revealing that point mutations in the 1,595-nt-long MEG3 can dramatically alter the ability of this lncRNA to stimulate the p53 pathway and regulate the cell cycle (Figures 4B and 4C).

Our comparative qRT-PCR, western blot, luciferase, and flow cytometry data on three human MEG3 splicing variants (v1, v3, and v9) and their structural mutants, in line with previous literature reports (Zhang et al., 2003, 2010b; Zhou et al., 2007, 2012; Zhu et al., 2015), suggest that p53 stimulation by MEG3 occurs via at least two different mechanisms (Figure 7): (1) stimulation of p53 expression (Figure 4E) and (2) upregulation of p53 target genes (Figure 4F). Although this latter effect is necessarily partly induced by the increased levels of p53, MEG3 seems to also participate directly in the upregulation of p53 target genes because splice variants and mutants display selectivity on different p53REs (Figures 3K and 5E) and because, in our system, MEG3 arrests the cell cycle but does not induce apoptosis (Figures 3A–3C and 3F–3H, respectively). As proposed previously (Zhou et al., 2007; Zhu et al., 2015), and in analogy with other lncRNAs like DINO (Schmitt et al., 2016), these direct effects of MEG3 on p53 target gene expression may be due to direct binding of MEG3 to p53 protein (Figures 4G and 4H), but p53-RNA interactions must be interpreted with caution because they are likely promiscuous (Riley and Maher, 2007).

Key MEG3 Functional Motifs Form Intramolecular Long-Range Interactions, Not Protein-Binding Hubs

Even more remarkably, our functional, evolutionary, and biochemical data show that H11 and H27 are structurally connected via functionally important long-range tertiary interactions. Previous studies had revealed the potential for lncRNAs to form long-range tertiary interactions, such as duplexes in the A-repeats of human XIST (Lu et al., 2016) or long-range *in vitro* cross-links in mouse RepA (Liu et al., 2017). However, the formation and physiological relevance of long-range tertiary interactions in lncRNAs had not yet been functionally validated. In our work, compensatory mutagenesis coupled to cellular assays revealed that the intramolecular pseudoknots (kissing loops) between H11 and the H27 TRs, which are located ~500 nt apart, are essential for function (Figures 2 and 5E). TR3 seems to be the preferred interaction partner of H11 because it is single-stranded in all three MEG3 variants (Figure S1). Moreover, $U^{869}G$ in TR3 is the most efficient compensatory mutation in rescuing activity of the $G^{370}C$ mutant (Figure 5E). However, all

MEG3 sequences that we identified in mammals possess at least 3 of the 6 TRs that characterize human MEG3, and covariation analyses reveal that the interaction of these TRs with the H11 terminal loop is conserved in evolution (Figure 2; Table S2). Additionally, compensatory mutagenesis on TR1, TR4, TR5, and TR6 in human MEG3 can also partly rescue activity of the $G^{370}C$ mutant, at least on certain target genes (Figure 5E). These findings suggest that H11 likely interacts with different H27 TRs, forming multiple alternative and mutually exclusive conformations. Such redundancy is reminiscent of the redundancy observed in protein repeats (Andrade et al., 2001) and may be functionally important. While in proteins, internal repetition confers the advantage of creating larger binding surface areas for cofactors (Andrade et al., 2001), for MEG3, the alternative interactions between H11 and H27 may generate slightly different structure motifs to fine-tune p53 stimulation on different target genes.

Independent of how the different H11–H27 pseudoknots exactly modulate the MEG3 structure, our cellular and biochemical data establish that these motifs behave very differently from other typical lncRNA functional motifs studied to date. For instance, HOTAIR domains 1 and 4 interact with PRC2 and LSD1, respectively (Somarowthu et al., 2015), the AGIL motif in lncRNA BRAVEHEART interacts with protein CNBP (Xue et al., 2016), the roX tandem repeats interact with MLE and MSL2 (Ilik et al., 2013), and the XIST repeat A duplexes interact with SPEN (Lu et al., 2016). In contrast, the MEG3 H11–H27 kissing loops form a *bona fide* intramolecular interaction, not a protein-binding site (Figures 1F and 4G and 4H).

MEG3 Mutants that Lose Function *In Vivo* Display Folding Defects *In Vitro*

Despite H11–H27 likely not being direct protein-binding sites, MEG3 does work in association with proteins (Liu et al., 2015; Sherpa et al., 2018; Zhu et al., 2015), and its structure is inevitably modulated by protein binding in the cell. Our *in vivo*/*ex vivo* SHAPE data reveal the specific regions where protein-binding sites localize in the MEG3 structure. These regions involve functionally important motifs, such as H16–H24 (D3) and the variable D4 and D5. Although identification of the exact protein partners of MEG3 goes beyond the scopes of this work, our data constitute important premises for systematic interactome studies, such as hybridization capture assays used previously for other lncRNAs (Chu and Chang, 2016; Simon, 2013).

In our work, we limited our analysis to the characterization of the MEG3 core because our identification of the H11–H27 long-range pairing directed us to analyze this intramolecular interaction systematically using an integrative *in vitro*/*in vivo* approach that combines compensatory mutagenesis with evolutionary, structural, and functional assays. Proving the functional importance of the H11–H27 pseudoknot confirms what had so far remained a speculative molecular mechanism assigned to lncRNAs: tertiary structure motifs can guide lncRNA function (Diederichs, 2014; Novikova et al., 2012a). Moreover, establishing the functional relevance of the H11–H27 long-range tertiary interaction *in vivo* opened up the way for exploring the *in vitro* tertiary structure of MEG3 using in-solution and single-particle imaging techniques that have never been employed before for lncRNAs. Remarkably, minimal mutations in functionally relevant MEG3 motifs induce pronounced differences in the hydrodynamic properties of this lncRNA (ΔR_h, ~8% between v1 and H11LpA; Figure 5B), and such folding defects also emerge by chemical probing and AFM (Figures 5A, 5D, and 6). Although it remains to be established whether the MEG3 structure is equally compact *in vivo* as *in vitro*, our data show that the functionally important H11–H27 pseudoknots guide MEG3 folding *in vitro*. Such observation makes it tempting to speculate that MEG3 folding serves to spatially organize the MEG3 functional domains in the cell for correct orientation of its partner proteins and for proper modulation of gene expression.

Although the precise cascade of events that lead to MEG3-dependent stimulation of the p53 response remains to be elucidated, our data surprisingly show that MEG3 must specifically preserve the structural interaction between H11 and H27 for p53 stimulation (Figure 7). Because MEG3-dependent p53 stimulation contributes to the prevention of tumors, in which p53 is mostly expressed in its active, wild-type form (Cheunsuchon et al., 2011; Ellison et al., 1995; Levy et al., 1994; Nagashima et al., 1999; Suliman et al., 2001; Zhou et al., 2012) (i.e., pituitary adenoma; prevalence rate of ~15%; Ezzat et al., 2004; or meningioma; ~30% of all primary brain and central nervous system tumors; Wiemels et al., 2010), stabilizing the H11–H27 structure may become a powerful therapeutic approach to potentiate the p53 response and bypass the need of invasive intracranial surgery. Screening for structure-disrupting mutations in the MEG3 gene, particularly in the two key functional motifs H11 and H27, may also serve as a useful biomarker for identifying patients with increased cancer susceptibility. More generally, the fact that structure-function relationships for MEG3 can be dissected with high precision, even by point mutations, raises the prospect of gaining considerable mechanistic insights into the function and 3D architecture of many other lncRNAs through analogous studies.

STAR★METHODS

Detailed methods are provided in the online version of this paper and include the following:

- KEY RESOURCES TABLE
- LEAD CONTACT AND MATERIALS AVAILABILITY
- EXPERIMENTAL MODEL AND SUBJECT DETAILS
  - Mammalian cell lines
- METHODS DETAILS
  - Cloning and mutagenesis
  - *In vitro* transcription and purification
  - Native gel electrophoresis
  - Analytical ultracentrifugation (AUC)
  - Dynamic light scattering (DLS)
  - Size-exclusion chromatography coupled to multi-angle laser light scattering (SEC-MALLS)
  - Size-exclusion chromatography coupled to small-angle X-ray scattering (SEC-SAXS)
  - *In vitro* secondary structure probing (*in vitro* SHAPE)
  - *In vivo* secondary structure probing (*in vivo* SHAPE)
  - *Ex vivo* secondary structure probing (*ex vivo* SHAPE)

- ○ Hydroxyl radical footprinting (HRF)
- ○ Atomic force microscopy (AFM)
- ○ Quantitative real-time PCR (qRT-PCR)
- ○ Western blot
- ○ Luciferase assay
- ○ Cell cycle and apoptosis assays by flow cytometry
- ○ RNA immunoprecipitation (RIP)
- ○ Pull-down assays
- ○ Sequence and structural alignments
- QUANTIFICATION AND STATISTICAL ANALYSIS
- DATA AND CODE AVAILABILITY

SUPPLEMENTAL INFORMATION

Supplemental Information can be found online at https://doi.org/10.1016/j.molcel.2019.07.025.

ACKNOWLEDGMENTS

We thank A. Aubert for support with using the Eukaryotic Expression Facility at EMBL Grenoble; Dr. Y. Zhou (Massachusetts General Hospital, Boston) for sharing the pCMS-d2EGFP-MEG3, pCI-control, pCI-MEG3, p53-Luc, pCMVbeta, and pCI-p14^{ARF} vectors; Prof. H. Chang (Stanford University) for sharing the pcDNA-human DINO vector; the beamline scientists at the BM29-BioSAXS beamline for their support during SAXS data collection; Prof. A. Pyle and R. Tavares (Yale University) for sharing RScape scripts before publication; Dr. M. Paulsen (EMBL Heidelberg), Dr. M. Peyzet (IAB Grenoble), and Dr. J.-P. Kleman (IBS Grenoble) for support with flow cytometry; A. Leroy and Dr. C. Ebel (IBS Grenoble) for support with AUC; S. Acajjaoui and Dr. M. Soler Lopez (ESRF Grenoble) for support with DLS; Dr. C. Mas (IBSG Grenoble) and Prof. M. Jamin (UGA Grenoble) for support with SEC-MALLS; and the EMBL Gene Core Facility (EMBL Heidelberg) for performing high-throughput sequencing related to *in vivo* SHAPE. We also thank Dr. C. Petosa (IBS Grenoble), Dr. A. Verdel (IAB Grenoble), Dr. A. Bateman (EMBL-EBI Hinxton), Dr. J.A. Márquez (EMBL Grenoble), Dr. M. Hentze (EMBL Heidelberg), Prof. G. Capranico (Bologna University), Dr. M. Huarte (Centro de Investigación Médica Aplicada, University of Navarra), and all members of the Marcia lab for helpful discussions. Work in the Marcia lab is partly funded by the Agence Nationale de la Recherche (ANR-15-CE11-0003-01), the Agence Nationale de Recherche sur le Sida et les Hépatites Virales (ANRS, ECTZ18552), and ITMO Cancer (18CN047-00). The Marcia lab uses the platforms of the Grenoble Instruct Center (ISBG UMS 3518 CNRS-CEA-UJF-EMBL) with support from FRISBI (ANR-10-INSB-05-02) and GRAL (ANR-10-LABX-49-01) within the Grenoble Partnership for Structural Biology (PSB). IBS acknowledges integration into the Interdisciplinary Research Institute of Grenoble (IRIG, CEA). This work acknowledges the AFM platform at the IBS.

AUTHOR CONTRIBUTIONS

T.U. produced v1 and v9; performed and analyzed *in vitro* and *in vivo* SHAPE and DMS probing, electrophoresis, AUC, MALLS, DLS, SAXS, and AFM data; performed luciferase assays on the mutants and related cloning; and wrote an original draft of the manuscript. E.A. produced v3, performed and analyzed *in vitro* SHAPE of v3 and related cloning, and performed and analyzed HRF data of v1. A.R. performed RIP. J.-M.T. and J.-L.P. performed AFM imaging and provided assistance, know-how, reagents, and instrumentation for AFM. P.A. contributed to analysis of the AFM data. O.P. performed western blot analysis on p53 and p53 target genes. A.I. provided constructs and know-how for luciferase reporter assays and supervised the RIP analysis. I.C. designed the research, supervised the *in vitro* probing analysis, optimized *in vivo* probing and luciferase assays, and performed flow cytometry, qRT-PCR, and pull-down experiments and related cloning. M.M. designed and supervised the research, obtained funding and resources, analyzed data, performed the evolutionary analysis, and wrote the manuscript. All authors reviewed and edited the manuscript and approved the final draft.

DECLARATION OF INTERESTS

The authors declare no competing interests.

Received: January 30, 2019
Revised: June 11, 2019
Accepted: July 15, 2019
Published: August 20, 2019

REFERENCES

Andrade, M.A., Perez-Iratxeta, C., and Ponting, C.P. (2001). Protein repeats: structures, functions, and evolution. J. Struct. Biol. *134*, 117–131.

Belyi, V.A., Ak, P., Markert, E., Wang, H., Hu, W., Puzio-Kuter, A., and Levine, A.J. (2010). The origins and evolution of the p53 family of genes. Cold Spring Harb. Perspect. Biol. *2*, a001198.

Busan, S., and Weeks, K.M. (2018). Accurate detection of chemical modifications in RNA by mutational profiling (MaP) with ShapeMapper 2. RNA *24*, 143–148.

Cabili, M.N., Dunagin, M.C., McClanahan, P.D., Biaesch, A., Padovan-Merhar, O., Regev, A., Rinn, J.L., and Raj, A. (2015). Localization and abundance analysis of human lncRNAs at single-cell and single-molecule resolution. Genome Biol. *16*, 20.

Calò, A., Stoliar, P., Bystrenova, E., Valle, F., and Biscarini, F. (2009). Measurement of DNA morphological parameters at highly entangled regime on surfaces. J. Phys. Chem. B *113*, 4987–4990.

Chen, S.W., and Pellequer, J.L. (2011). DeStripe: frequency-based algorithm for removing stripe noises from AFM images. BMC Struct. Biol. *11*, 7.

Chen, Y., and Pollack, L. (2016). SAXS studies of RNA: structures, dynamics, and interactions with partners. Wiley Interdiscip. Rev. RNA *7*, 512–526.

Cheunsuchon, P., Zhou, Y., Zhang, X., Lee, H., Chen, W., Nakayama, Y., Rice, K.A., Tessa Hedley-Whyte, E., Swearingen, B., and Klibanski, A. (2011). Silencing of the imprinted DLK1-MEG3 locus in human clinically nonfunctioning pituitary adenomas. Am. J. Pathol. *179*, 2120–2130.

Chillón, I., and Pyle, A.M. (2016). Inverted repeat *Alu* elements in the human lincRNA-p21 adopt a conserved secondary structure that regulates RNA function. Nucleic Acids Res. *44*, 9462–9471.

Chillón, I., Marcia, M., Legiewicz, M., Liu, F., Somarowthu, S., and Pyle, A.M. (2015). Native purification and analysis of long RNAs. Methods Enzymol. *558*, 3–37.

Chu, C., and Chang, H.Y. (2016). Understanding RNA-Chromatin Interactions Using Chromatin Isolation by RNA Purification (ChIRP). Methods Mol. Biol. *1480*, 115–123.

Darty, K., Denise, A., and Ponty, Y. (2009). VARNA: Interactive drawing and editing of the RNA secondary structure. Bioinformatics *25*, 1974–1975.

Delagenière, S., Brenchereau, P., Launer, L., Ashton, A.W., Leal, R., Veyrier, S., Gabadinho, J., Gordon, E.J., Jones, S.D., Levik, K.E., et al. (2011). ISPyB: an information management system for synchrotron macromolecular crystallography. Bioinformatics *27*, 3186–3192.

Diederichs, S. (2014). The four dimensions of noncoding RNA conservation. Trends Genet. *30*, 121–123.

Ellison, D.W., Lunec, J., Gallagher, P.J., Steart, P.V., Jaros, E., and Gatter, K.C. (1995). Accumulation of wild-type p53 in meningiomas. Neuropathol. Appl. Neurobiol. *21*, 136–142.

Ezzat, S., Asa, S.L., Couldwell, W.T., Barr, C.E., Dodge, W.E., Vance, M.L., and McCutcheon, I.E. (2004). The prevalence of pituitary adenomas: a systematic review. Cancer *101*, 613–619.

Folta-Stogniew, E. (2006). Oligomeric states of proteins determined by size-exclusion chromatography coupled with light scattering, absorbance, and refractive index detectors. Methods Mol. Biol. *328*, 97–112.

García-Sacristán, A., Moreno, M., Ariza-Mateos, A., López-Camacho, E., Jáudenes, R.M., Vázquez, L., Gómez, J., Martín-Gago, J.A., and Briones, C. (2015). A magnesium-induced RNA conformational switch at the internal

ribosome entry site of hepatitis C virus genome visualized by atomic force microscopy. Nucleic Acids Res. *43*, 565–580.

Giro, A., Bergia, A., Zuccheri, G., Bink, H.H., Pleij, C.W., and Samorì, B. (2004). Single molecule studies of RNA secondary structure: AFM of TYMV viral RNA. Microsc. Res. Tech. *65*, 235–245.

Hansma, H.G., Revenko, I., Kim, K., and Laney, D.E. (1996). Atomic force microscopy of long and short double-stranded, single-stranded and triple-stranded nucleic acids. Nucleic Acids Res. *24*, 713–720.

Hawkes, E.J., Hennelly, S.P., Novikova, I.V., Irwin, J.A., Dean, C., and Sanbonmatsu, K.Y. (2016). COOLAIR antisense RNAs form evolutionarily conserved elaborate secondary structures. Cell Rep. *16*, 3087–3096.

Higuchi, T. (1988). Approach to an irregular time-series on the basis of the fractal theory. Physica D *31*, 277–283.

Ilik, I.A., Quinn, J.J., Georgiev, P., Tavares-Cadete, F., Maticzka, D., Toscano, S., Wan, Y., Spitale, R.C., Luscombe, N., Backofen, R., et al. (2013). Tandem stem-loops in roX RNAs act together to mediate X chromosome dosage compensation in Drosophila. Mol. Cell *51*, 156–173.

Jacques, D.A., and Trewhella, J. (2010). Small-angle scattering for structural biology–expanding the frontier while avoiding the pitfalls. Protein Sci. *19*, 642–657.

Kaneko, S., Bonasio, R., Saldaña-Meyer, R., Yoshida, T., Son, J., Nishino, K., Umezawa, A., and Reinberg, D. (2014). Interactions between JARID2 and noncoding RNAs regulate PRC2 recruitment to chromatin. Mol. Cell *53*, 290–300.

Karabiber, F., McGinnis, J.L., Favorov, O.V., and Weeks, K.M. (2013). QuShape: rapid, accurate, and best-practices quantification of nucleic acid probing information, resolved by capillary electrophoresis. RNA *19*, 63–73.

Kaushik, K., Leonard, V.E., Kv, S., Lalwani, M.K., Jalali, S., Patowary, A., Joshi, A., Scaria, V., and Sivasubbu, S. (2013). Dynamic expression of long non-coding RNAs (lncRNAs) in adult zebrafish. PLoS ONE *8*, e83616.

Keene, J.D., Komisarow, J.M., and Friedersdorf, M.B. (2006). RIP-Chip: the isolation and identification of mRNAs, microRNAs and protein components of ribonucleoprotein complexes from cell extracts. Nat. Protoc. *1*, 302–307.

Kent, W.J. (2002). BLAT–the BLAST-like alignment tool. Genome Res. *12*, 656–664.

Levy, A., Hall, L., Yeudall, W.A., and Lightman, S.L. (1994). p53 gene mutations in pituitary adenomas: rare events. Clin. Endocrinol. (Oxf.) *41*, 809–814.

Li, M.Z., and Elledge, S.J. (2012). SLIC: a method for sequence- and ligation-independent cloning. Methods Mol. Biol. *852*, 51–59.

Li, W., Cowley, A., Uludag, M., Gur, T., McWilliam, H., Squizzato, S., Park, Y.M., Buso, N., and Lopez, R. (2015). The EMBL-EBI bioinformatics web and programmatic tools framework. Nucleic Acids Res. *43* (W1), W580-4.

Lin, Y., Schmidt, B.F., Bruchez, M.P., and McManus, C.J. (2018). Structural analyses of NEAT1 lncRNAs suggest long-range RNA interactions that may contribute to paraspeckle architecture. Nucleic Acids Res. *46*, 3742–3752.

Liu, S., Zhu, J., Jiang, T., Zhong, Y., Tie, Y., Wu, Y., Zheng, X., Jin, Y., and Fu, H. (2015). Identification of lncRNA MEG3 Binding Protein Using MS2-Tagged RNA Affinity Purification and Mass Spectrometry. Appl. Biochem. Biotechnol. *176*, 1834–1845.

Liu, F., Somarowthu, S., and Pyle, A.M. (2017). Visualizing the secondary and tertiary architectural domains of lncRNA RepA. Nat. Chem. Biol. *13*, 282–289.

Lu, K.H., Li, W., Liu, X.H., Sun, M., Zhang, M.L., Wu, W.Q., Xie, W.P., and Hou, Y.Y. (2013). Long non-coding RNA MEG3 inhibits NSCLC cells proliferation and induces apoptosis by affecting p53 expression. BMC Cancer *13*, 461.

Lu, Z., Zhang, Q.C., Lee, B., Flynn, R.A., Smith, M.A., Robinson, J.T., Davidovich, C., Gooding, A.R., Goodrich, K.J., Mattick, J.S., et al. (2016). RNA Duplex Map in Living Cells Reveals Higher-Order Transcriptome Structure. Cell *165*, 1267–1279.

Lyubchenko, Y.L., Shlyakhtenko, L.S., and Ando, T. (2011). Imaging of nucleic acids with atomic force microscopy. Methods *54*, 274–283.

Marcia, M., and Pyle, A.M. (2012). Visualizing group II intron catalysis through the stages of splicing. Cell *151*, 497–507.

Marín-Béjar, O., and Huarte, M. (2015). RNA pulldown protocol for in vitro detection and identification of RNA-associated proteins. Methods Mol. Biol. *1206*, 87–95.

Mathews, D.H. (2004). Using an RNA secondary structure partition function to determine confidence in base pairs predicted by free energy minimization. RNA *10*, 1178–1190.

McMurray, E.N., and Schmidt, J.V. (2012). Identification of imprinting regulators at the Meg3 differentially methylated region. Genomics *100*, 184–194.

Menendez, D., Inga, A., and Resnick, M.A. (2010). Estrogen receptor acting in cis enhances WT and mutant p53 transactivation at canonical and noncanonical p53 target sequences. Proc. Natl. Acad. Sci. USA *107*, 1500–1505.

Mercer, T.R., Dinger, M.E., Sunkin, S.M., Mehler, M.F., and Mattick, J.S. (2008). Specific expression of long noncoding RNAs in the mouse brain. Proc. Natl. Acad. Sci. USA *105*, 716–721.

Mercer, T.R., Dinger, M.E., and Mattick, J.S. (2009). Long non-coding RNAs: insights into functions. Nat. Rev. Genet. *10*, 155–159.

Miyoshi, N., Wagatsuma, H., Wakana, S., Shiroishi, T., Nomura, M., Aisaka, K., Kohda, T., Surani, M.A., Kaneko-Ishino, T., and Ishino, F. (2000). Identification of an imprinted gene, Meg3/Gtl2 and its human homologue MEG3, first mapped on mouse distal chromosome 12 and human chromosome 14q. Genes Cells *5*, 211–220.

Mondal, T., Subhash, S., Vaid, R., Enroth, S., Uday, S., Reinius, B., Mitra, S., Mohammed, A., James, A.R., Hoberg, E., et al. (2015). MEG3 long noncoding RNA regulates the TGF-β pathway genes through formation of RNA-DNA triplex structures. Nat. Commun. *6*, 7743.

Nagashima, G., Aoyagi, M., Yamamoto, M., Yamamoto, S., Wakimoto, H., Ohno, K., Yamamoto, K., and Hirakawa, K. (1999). P53 overexpression and proliferative potential in malignant meningiomas. Acta Neurochir. (Wien) *141*, 53–61, discussion 60–61.

Nawrocki, E.P., and Eddy, S.R. (2013). Infernal 1.1: 100-fold faster RNA homology searches. Bioinformatics *29*, 2933–2935.

Necas, D., and Klapetek, P. (2012). Gwyddion: an open-source software for SPM data analysis. Cent. Eur. J. Phys. *10*, 181–188.

Necsulea, A., Soumillon, M., Warnefors, M., Liechti, A., Daish, T., Zeller, U., Baker, J.C., Grützner, F., and Kaessmann, H. (2014). The evolution of lncRNA repertoires and expression patterns in tetrapods. Nature *505*, 635–640.

Novikova, I.V., Hennelly, S.P., and Sanbonmatsu, K.Y. (2012a). Sizing up long non-coding RNAs: do lncRNAs have secondary and tertiary structure? Bioarchitecture *2*, 189–199.

Novikova, I.V., Hennelly, S.P., and Sanbonmatsu, K.Y. (2012b). Structural architecture of the human long non-coding RNA, steroid receptor RNA activator. Nucleic Acids Res. *40*, 5034–5051.

Patel, T.R., Chojnowski, G., Astha, Koul, A., McKenna, S.A., and Bujnicki, J.M. (2017). Structural studies of RNA-protein complexes: A hybrid approach involving hydrodynamics, scattering, and computational methods. Methods *118-119*, 146–162.

Petoukhov, M.V., Franke, D., Shkumatov, A.V., Tria, G., Kikhney, A.G., Gajda, M., Gorba, C., Mertens, H.D., Konarev, P.V., and Svergun, D.I. (2012). New developments in the *ATSAS* program package for small-angle scattering data analysis. J. Appl. Cryst. *45*, 342–350.

Pfaffl, M.W. (2001). A new mathematical model for relative quantification in real-time RT-PCR. Nucleic Acids Res. *29*, e45.

Reuter, J.S., and Mathews, D.H. (2010). RNAstructure: software for RNA secondary structure prediction and analysis. BMC Bioinformatics *11*, 129.

Rice, G.M., Leonard, C.W., and Weeks, K.M. (2014). RNA secondary structure modeling at consistent high accuracy using differential SHAPE. RNA *20*, 846–854.

Riley, K.J., and Maher, L.J., 3rd (2007). p53 RNA interactions: new clues in an old mystery. RNA *13*, 1825–1833.

Rivas, E., Clements, J., and Eddy, S.R. (2017). A statistical test for conserved RNA structure shows lack of evidence for structure in lncRNAs. Nat. Methods *14*, 45–48.

Sauvageau, M., Goff, L.A., Lodato, S., Bonev, B., Groff, A.F., Gerhardinger, C., Sanchez-Gomez, D.B., Hacisuleyman, E., Li, E., Spence, M., et al. (2013). Multiple knockout mouse models reveal lincRNAs are required for life and brain development. eLife *2*, e01749.

Schmitt, A.M., Garcia, J.T., Hung, T., Flynn, R.A., Shen, Y., Qu, K., Payumo, A.Y., Peres-da-Silva, A., Broz, D.K., Baum, R., et al. (2016). An inducible long noncoding RNA amplifies DNA damage signaling. Nat. Genet. *48*, 1370–1376.

Schön, P. (2016). Imaging and force probing RNA by atomic force microscopy. Methods *103*, 25–33.

Schuck, P. (2000). Size-distribution analysis of macromolecules by sedimentation velocity ultracentrifugation and lamm equation modeling. Biophys. J. *78*, 1606–1619.

Sherpa, C., Rausch, J.W., and Le Grice, S.F.J. (2018). Structural characterization of maternally expressed gene 3 RNA reveals conserved motifs and potential sites of interaction with polycomb repressive complex 2. Nucleic Acids Res. *46*, 10432–10447.

Siegfried, N.A., Busan, S., Rice, G.M., Nelson, J.A., and Weeks, K.M. (2014). RNA motif discovery by SHAPE and mutational profiling (SHAPE-MaP). Nat. Methods *11*, 959–965.

Simon, M.D. (2013). Capture hybridization analysis of RNA targets (CHART). Curr. Protoc. Mol. Biol. *Chapter 21*, Unit 21.25.

Smola, M.J., Calabrese, J.M., and Weeks, K.M. (2015a). Detection of RNA-protein interactions in living cells with SHAPE. Biochemistry *54*, 6867–6875.

Smola, M.J., Rice, G.M., Busan, S., Siegfried, N.A., and Weeks, K.M. (2015b). Selective 2′-hydroxyl acylation analyzed by primer extension and mutational profiling (SHAPE-MaP) for direct, versatile and accurate RNA structure analysis. Nat. Protoc. *10*, 1643–1669.

Smola, M.J., Christy, T.W., Inoue, K., Nicholson, C.O., Friedersdorf, M., Keene, J.D., Lee, D.M., Calabrese, J.M., and Weeks, K.M. (2016). SHAPE reveals transcript-wide interactions, complex structural domains, and protein interactions across the Xist lncRNA in living cells. Proc. Natl. Acad. Sci. USA *113*, 10322–10327.

Somarowthu, S., Legiewicz, M., Chillón, I., Marcia, M., Liu, F., and Pyle, A.M. (2015). HOTAIR forms an intricate and modular secondary structure. Mol. Cell *58*, 353–361.

Su, L.J., Waldsich, C., and Pyle, A.M. (2005). An obligate intermediate along the slow folding pathway of a group II intron ribozyme. Nucleic Acids Res. *33*, 6674–6687.

Suliman, M., Royds, J., Cullen, D., Timperley, W., Powell, T., Battersby, R., and Jones, T.H. (2001). Mdm2 and the p53 pathway in human pituitary adenomas. Clin. Endocrinol. (Oxf.) *54*, 317–325.

Swisher, J., Duarte, C.M., Su, L.J., and Pyle, A.M. (2001). Visualizing the solvent-inaccessible core of a group II intron ribozyme. EMBO J. *20*, 2051–2061.

Tarver, J.E., Dos Reis, M., Mirarab, S., Moran, R.J., Parker, S., O'Reilly, J.E., King, B.L., O'Connell, M.J., Asher, R.J., Warnow, T., et al. (2016). The interrelationships of placental mammals and the limits of phylogenetic inference. Genome Biol. Evol. *8*, 330–344.

Tavares, R.C.A., Pyle, A.M., and Somarowthu, S. (2019). Phylogenetic Analysis with Improved Parameters Reveals Conservation in lncRNA Structures. J. Mol. Biol. *431*, 1592–1603.

Volders, P.J., Helsens, K., Wang, X., Menten, B., Martens, L., Gevaert, K., Vandesompele, J., and Mestdagh, P. (2013). LNCipedia: a database for annotated human lncRNA transcript sequences and structures. Nucleic Acids Res. *41*, D246–D251.

Wadley, L.M., Keating, K.S., Duarte, C.M., and Pyle, A.M. (2007). Evaluating and learning from RNA pseudotorsional space: quantitative validation of a reduced representation for RNA structure. J. Mol. Biol. *372*, 942–957.

Wapinski, O., and Chang, H.Y. (2011). Long noncoding RNAs and human disease. Trends Cell Biol. *21*, 354–361.

Weinberg, Z., and Breaker, R.R. (2011). R2R–software to speed the depiction of aesthetic consensus RNA secondary structures. BMC Bioinformatics *12*, 3.

Wiemels, J., Wrensch, M., and Claus, E.B. (2010). Epidemiology and etiology of meningioma. J. Neurooncol. *99*, 307–314.

Wilkinson, K.A., Merino, E.J., and Weeks, K.M. (2006). Selective 2′-hydroxyl acylation analyzed by primer extension (SHAPE): quantitative RNA structure analysis at single nucleotide resolution. Nat. Protoc. *1*, 1610–1616.

Woodson, S.A. (2005). Metal ions and RNA folding: a highly charged topic with a dynamic future. Curr. Opin. Chem. Biol. *9*, 104–109.

Woodson, S.A. (2010). Compact intermediates in RNA folding. Annu. Rev. Biophys. *39*, 61–77.

Xue, Z., Hennelly, S., Doyle, B., Gulati, A.A., Novikova, I.V., Sanbonmatsu, K.Y., and Boyer, L.A. (2016). A G-Rich motif in the lncRNA Braveheart interacts with a zinc-finger transcription factor to specify the cardiovascular lineage. Mol. Cell *64*, 37–50.

Yu, J., Liu, Z., Jiang, W., Wang, G., and Mao, C. (2015). De novo design of an RNA tile that self-assembles into a homo-octameric nanoprism. Nat. Commun. *6*, 5724.

Zhang, X., Zhou, Y., Mehta, K.R., Danila, D.C., Scolavino, S., Johnson, S.R., and Klibanski, A. (2003). A pituitary-derived MEG3 isoform functions as a growth suppressor in tumor cells. J. Clin. Endocrinol. Metab. *88*, 5119–5126.

Zhang, X., Gejman, R., Mahta, A., Zhong, Y., Rice, K.A., Zhou, Y., Cheunsuchon, P., Louis, D.N., and Klibanski, A. (2010a). Maternally expressed gene 3, an imprinted noncoding RNA gene, is associated with meningioma pathogenesis and progression. Cancer Res. *70*, 2350–2358.

Zhang, X., Rice, K., Wang, Y., Chen, W., Zhong, Y., Nakayama, Y., Zhou, Y., and Klibanski, A. (2010b). Maternally expressed gene 3 (MEG3) noncoding ribonucleic acid: isoform structure, expression, and functions. Endocrinology *151*, 939–947.

Zhou, Y., Zhong, Y., Wang, Y., Zhang, X., Batista, D.L., Gejman, R., Ansell, P.J., Zhao, J., Weng, C., and Klibanski, A. (2007). Activation of p53 by MEG3 non-coding RNA. J. Biol. Chem. *282*, 24731–24742.

Zhou, Y., Zhang, X., and Klibanski, A. (2012). MEG3 noncoding RNA: a tumor suppressor. J. Mol. Endocrinol. *48*, R45–R53.

Zhou, K.I., Liu, N., and Pan, T. (2017). Identification of N^6-methyladenosine reader proteins. Methods *126*, 105–111.

Zhu, J., Liu, S., Ye, F., Shen, Y., Tie, Y., Zhu, J., Wei, L., Jin, Y., Fu, H., Wu, Y., and Zheng, X. (2015). Long noncoding RNA MEG3 interacts with p53 protein and regulates partial p53 target genes in hepatoma cells. PLoS ONE *10*, e0139790.

STAR★METHODS

KEY RESOURCES TABLE

REAGENT or RESOURCE	SOURCE	IDENTIFIER
Antibodies		
anti BAX (D2D) Mouse IgG1 antibody	Santa Cruz Biotechnology, Inc. (Texas, USA)	Cat#sc-20067; RRID: AB_626726
anti p21 (187) Mouse IgG1 antibody	Santa Cruz Biotechnology, Inc. (Texas, USA)	Cat#sc-817; RRID: AB_628072
anti p53 (DO-1) Mouse IgG2a antibody	Santa Cruz Biotechnology, Inc. (Texas, USA)	Cat#sc-126; RRID: AB_628082
anti actin Rabbit IgG antibody	Abcam (UK)	Cat#ab1801; RRID: AB_302617
anti mouse IgG1 Alexa Fluor 647 (goat IgG)	Thermo Fisher Scientific (Massachusetts, USA)	Cat#A-21240; RRID: AB141658
anti mouse IgG (H+L) Alexa Fluor 647 (goat IgG)	Thermo Fisher Scientific (Massachusetts, USA)	Cat#A-32728; RRID: AB_2633277
anti rabbit IgG Alexa Fluor 488 (goat IgG)	Thermo Fisher Scientific (Massachusetts, USA)	Cat#A-32731; RRID: AB_2633280
Bacterial and Virus Strains		
E. coli Mach1 competent cells	Thermo Fisher Scientific (Massachusetts, USA)	Cat#C862003
Chemicals, Peptides, and Recombinant Proteins		
XbaI restriction enzyme	New England Biolabs (Massachusetts, USA)	Cat#R0145S
SacI restriction enzyme	New England Biolabs (Massachusetts, USA)	Cat#R3156S
NotI restriction enzyme	New England Biolabs (Massachusetts, USA)	Cat#R3189S
KpnI restriction enzyme	New England Biolabs (Massachusetts, USA)	Cat#R3142S
Turbo DNase	Thermo Fisher Scientific (Massachusetts, USA)	Cat#AM2238
Proteinase K	Thermo Fisher Scientific (Massachusetts, USA)	Cat#17916
1-methyl-7-nitroisatoic anhydride (1M7)	in house synthesis at the EMBL Chemical Biology Facility (Heidelberg, Germany)	not available
1-methyl-6-nitroisatoic anhydride (1M6)	Sigma Aldrich (France)	Cat#S888079-250MG
N-methylisatoic anhydride (NMIA)	Sigma Aldrich (France)	Cat#129887-100G
dimethyl sulfate (DMS)	Sigma Aldrich (France)	Cat#D186309
5(6)-FAM, SE	Tebu-bio (France)	Cat#AS-81006
6-JOE, SE	Tebu-bio (France)	Cat#AS-81011
McCoy's 5a medium modified	Thermo Fisher Scientific (Massachusetts, USA)	Cat#26600080
Minimum Essential Medium Eagle	Sigma Aldrich (France)	Cat#51416C-1000ML
RNeasy Mini Kit	QIAGEN (France)	Cat#74104
Zymogen RNA clean and concentrator kit	Zymo Research (California, USA)	Cat#R1019
RNA 6000 Nano chips	Agilent (California, USA)	Cat#5067-1511
SuperScript II reverse transcriptase	Thermo Fisher Scientific (Massachusetts, USA)	Cat#18064014
PCR clean up kit QIAquick	QIAGEN (France)	Cat#28104

(*Continued on next page*)

Continued

REAGENT or RESOURCE	SOURCE	IDENTIFIER
RNase A	Sigma Aldrich (France)	Cat#R6513-10MG
Q5 hot start DNA Polymerase	New England Biolabs (Massachusetts, USA)	Cat#M0494S
Nextera XT DNA library prep kit	Illumina (California, USA)	Cat#FC-131-1024
Nextera® XT Index Kit	Illumina (California, USA)	Cat#FC-131-1001
AMPure XP beads	Beckman Coulter (France)	Cat#A63881
PolyA RNA	GE Healthcare (France)	Cat#27-4110-01
4-12% NuPAGE® Bis-Tris Gels	Thermo Fisher Scientific (Massachusetts, USA)	Cat#NP0322BOX
12% NuPAGE® Bis-Tris Gels	Thermo Fisher Scientific (Massachusetts, USA)	Cat#NP0342BOX
UltraCruz® Blocking Reagent	Santa Cruz Biotechnology, Inc. (Texas, USA)	Cat#SC-516214
Lipofectamine 2000	Thermo Fisher Scientific (Massachusetts, USA)	Cat#11668030
Click-iT Plus EdU Flow Cytometry Assay Kit	Thermo Fisher Scientific (Massachusetts, USA)	Cat#C10634
LIVE/DEAD Fixable Dead Cell Stain	Thermo Fisher Scientific (Massachusetts, USA)	Cat#L10119
Brilliant Violet 421 annexin V	Biolegend (California, USA)	Cat#640923
Propidium Iodide	Biolegend (California, USA)	Cat#421301
RevertAid First Strand cDNA Synthesis Kit	Thermo Fisher Scientific (Massachusetts, USA)	Cat#K1622
qPCRBIO SyGreen Mix	PCR Biosystems (UK)	Cat#PB20.16-01
Nutlin-3	Cayman Chemical (Michigan, USA)	Cat#10004372
Deposited Data		
Ex vivo SHAPE	BioProject	PRJNA552583
In vivo SHAPE	BioProject	PRJNA552583
Experimental Models: Cell Lines		
HCT116 p53+/+ and HCT116 p53−/− isogenic cell lines	Horizon Discovery (UK)	Cat#HD104-001
WI38 fibroblast	ECACC (UK)	90020107
Oligonucleotides		
reported in Table S3	This Paper	N/A
Recombinant DNA		
p53-Luc vector	Dr. Yunli Zhou, Massachusetts General Hospital (USA)	Zhou et al., 2007
pcDNA-humanDINO	Dr. Howard Chang, Stanford University (USA)	Schmitt et al., 2016
pRL Renilla Luciferase Control Reporter Vector	Promega (France)	Cat#E1910
pCI-control	Dr. Yunli Zhou, Massachusetts General Hospital (USA)	Zhou et al., 2007
pCMS-d2EGFP-MEG3	Dr. Yunli Zhou, Massachusetts General Hospital (USA)	Zhou et al., 2007
pCI-MEG3	Dr. Yunli Zhou, Massachusetts General Hospital (USA)	Zhou et al., 2007
pCMVbeta	Dr. Yunli Zhou, Massachusetts General Hospital (USA)	Zhou et al., 2007
pCI-p14ARF	Dr. Yunli Zhou, Massachusetts General Hospital (USA)	Zhou et al., 2007

(*Continued on next page*)

Continued		
REAGENT or RESOURCE	SOURCE	IDENTIFIER
Software and Algorithms		
Unicorn	GE Healthcare (Illinois, USA)	version 5.20
ASTRA	Wyatt Technology (California, USA)	version 6.1
Zetasizer	Malvern Instruments (UK)	version 7.11
ISpyB	Delagenière et al., 2011	version 5.4.5
NanoScope	Bruker (Massachusetts, USA)	version 9.2
ClarioSTAR	BMG Labtech (France)	version 5.21 R4
MxPro	Stratagene (California, USA)	version 4.0.1.0
DIVA	BD Biosciences (France)	version 6.3.1
BsXCube	https://github.com/maaeli/BsxCuBE	version spec 6.03.11 (https://certif.com/spec.html)
QuBIT	Thermo Fisher Scientific (Massachusetts, USA)	version 3.0 Fluorometer APP 1.02 + MCU 0.21
BioAnalyzer	Agilent Technologies (California, USA)	version 2100 Expert B.02.08 SI648 (SR3)
Nanodrop 2000/2000C	Thermo Fisher Scientific (Massachusetts, USA)	version 1.6.198
Sedfit	Schuck, 2000	version 14.6e
GUSSI	The University of Texas Southwestern Medical Center	version 1.08
Prism	GraphPad Software Inc (California, USA)	version 6.05
ATSAS	Petoukhov et al., 2012	version 2.7.2-5
QuSHAPE	Karabiber et al., 2013	version 1.0
RNAStructure	Mathews, 2004	version 5.8.1
SuperFold	Siegfried et al., 2014	version 1.0
VaRNA	Darty et al., 2009	version 3-93
ShapeMapper	Busan and Weeks, 2018	version 2.1.3
deltaSHAPE	Smola et al., 2015a	version 0.91
Gwyddion	Necas and Klapetek, 2012	version 2.51
DeStripe	Chen and Pellequer, 2011	N/A
IgorPro	WaveMetrics (Oregon, USA)	N/A
CloneManager Professional Suite	Sci Ed Central (USA)	version 6.00
Primer Design	Sci Ed Central (USA)	version 4.20
OligoAnalyzer	Integrated DNA Technologies (Iowa, USA)	version 3.1
CS Express 6 Flow Research Edition	De Novo Software (California, USA)	version v6.05.0028
BLAT	Kent, 2002	https://genome.ucsc.edu/cgi-bin/hgBlat?command=start
Clustal Omega	Li et al., 2015	https://www.ebi.ac.uk/Tools/msa/clustalo/
Infernal	Nawrocki and Eddy, 2013	version 1.1.2
R2R	Weinberg and Breaker, 2011	version 1.0.5
RScape	Rivas et al., 2017; Tavares et al., 2019	version 1.2.3
MARS Data Analysis Software	BMG Labtech (France)	version 3.20 R2
Microsoft Office	Microsoft (California, USA)	version 2013

LEAD CONTACT AND MATERIALS AVAILABILITY

Further information and requests for resources and reagents should be directed to and will be fulfilled by the Lead Contact, Marco Marcia (mmarcia@embl.fr).

EXPERIMENTAL MODEL AND SUBJECT DETAILS

Mammalian cell lines

HCT116 p53$^{+/+}$ and p53$^{-/-}$ (Horizon Discovery) cell lines (both male, adult) were grown in McCoy's 5a medium modified (Life Technologies) supplemented with fetal bovine serum to a final concentration of 10%. WI38 (ECACC 90020107) fibroblast cell line (female, 3 month gestation fetus) was grown in Minimum Essential Medium Eagle (Sigma) supplemented with fetal bovine serum to a final concentration of 10% and 2 mM L-Glutamine.

METHODS DETAILS

Cloning and mutagenesis

A plasmid containing the sequence of human v1 was obtained by gene synthesis [GeneArt (Life Technologies)]. From this synthetic vector, the sequence of MEG3 was amplified by PCR and inserted by sequence- and ligation-independent cloning (SLIC) (Li and Elledge, 2012) into the scaffold of a modified pBluescript vector immediately downstream of a T7 promoter sequence and immediately upstream of an *Xba*I restriction site. The resulting vector was named pTU1. All pBluescript based vectors were used for *in vitro* transcription. Plasmid pTU2 containing v9 was created by deleting the sequence corresponding to E5 (nucleotides 936-1049) from pTU1 by quick change mutagenesis. Plasmid pTU1a containing v3 was created in two steps. First, the first 24 nt of v1 were deleted by quick change from pTU1. Second, E6 was inserted using four self-annealing primers and a DNA oligonucleotide by overlapping PCR and SLIC. Plasmids pTU3-pTU7 contain 5 different domains of v1, domain1 (2-196), domain 2 (230-410), domain 3 (471-902), domain 4 (951-1113) and domain5 (1116-1486), respectively, determined according to the secondary structure map. All domains were amplified by PCR from pTU1 and inserted by SLIC into the scaffold of pTU1 (between T7 promoter sequence and *Xba*I restriction site). Plasmid pTU123 was created by mutating the terminal loop of the H11 to poly A in pTU1 with quick change mutagenesis.

pCMS-d2-MEG3 was a kind gift of Yunli Zhou (Zhou et al., 2007). All pCMS-d2-MEG3 based vectors were used for *in vivo* assays by flow cytometry. Plasmid pTU8 (pCMS-d2-MEG3v1) was created by amplifying MEG3 and adding *Sac*I restriction site at 5′ and *Not*I restriction site on 3′ by PCR from pTU1 and inserting it in pCMS-d2-MEG3 with quick ligation between *Sac*I and *Not*I. Two complementary oligonucleotides SNf and SNr containing a *Sac*I restriction site, 13 nt sequence (5′-GGTTCACTAAACG-3′) and *Not*I restriction site were ordered from Eurofins (5′- CCGTTTAGTGAACCGC-3′, 5′- GGCCGCGGTTCACTAAACGGAGCT-3′). Plasmid pTU9 (pCMS-d2-empty) was created by annealing SNf and SNr by incubating 2 min at 95°C and letting it cool down to RT gently and inserting the resulting fragment in pCMS-d2-MEG3 with quick ligation between *Sac*I and *Not*I. MEG3 variants 1 and 9 were cloned in pcDNA3 vector between KpnI and *Not*I restriction sites. Different structural mutants were cloned in pCMS-d2-MEG3v1 (for flow cytometry assay) and pcDNA3 (for luciferase assay) by quick change or SLIC mutagenesis. The presence of the target gene in all plasmids was confirmed by enzyme digestion or colony PCR and agarose gel electrophoresis. Sequence of all vectors was validated by DNA sequencing (Eurofins). *E. coli* Mach1 competent cells were used for cloning. Plasmids were extracted with mini and maxi preps (QIAGEN) from a single colony.

In vitro transcription and purification

MEG3 was expressed and purified under non-denaturing conditions, as previously described (Chillón et al., 2015) with minor modifications. Briefly, plasmids pTU1-pTU7 and pTU123 were linearized overnight with restriction enzyme *Xba*I (NEB). The linearized vectors were transcribed *in vitro* with T7 polymerase in 100 mM $MgCl_2$, 400 mM TrisHCl pH 8.0, 20 mM spermidine, 100 mM DTT. Following transcription, template DNA and proteins were removed with Turbo DNase (Thermo Scientific) and proteinase K (Promega), respectively. Divalent ions were chelated with EDTA in the presence of physiological concentrations of monovalent ions for accurate subsequent titration of magnesium concentrations in folding experiments. Samples were then rebuffered in 0.1 M KCl, 8 mM K-MOPS pH 6.5, 0.1 mM Na-EDTA using Amicon Ultra-0.5 centrifugal concentrators (molecular weight cut-off of 100 kDa) finally subjected to a polishing size-exclusion chromatography (SEC) step using Tricorn columns (GE Healthcare) self-packed with Sephacryl S500 resin and run in 0.1 M KCl, 8 mM K-MOPS pH 6.5, 0.1 mM Na-EDTA if not otherwise specified.

Native gel electrophoresis

1% agarose gels were run in 1x Tris-Borate (TB) buffer (89 mM Tris base, 89 mM boric acid) supplemented with the indicated concentrations of Mg^{2+}. Gels in TB with no Mg^{2+} and with 2 mM Mg^{2+} were run for 45 min at 110 V, and gels in TB with 5 and 10 mM Mg^{2+} were run for 120 min at 80 V. Samples were mixed in a 5:1 ratio with 6x RNA native gel dye (0.5x TB buffer, 40% sucrose, 0.5% w/V orange G) before gel loading. Gels were stained with 1x SYBR Safe gel stain in 1x TB buffer for 1 h at room temperature before exposure (Invitrogen).

Analytical ultracentrifugation (AUC)

Analytical ultracentrifugation (AUC) sedimentation velocity experiments were performed as described (Chillón et al., 2015). Purified MEG3 was supplemented with varying concentrations of $MgCl_2$ ranging from 0.01 mM to 100 mM. Samples were analyzed using Beckman XL-A/XL-I centrifuge with AN-50 Ti rotor (Beckman Coulter). All experiments were performed at 20°C at 25,000 rpm overnight. Data were analyzed with Sedfit using continuous c(s) distribution model (Schuck, 2000).

Dynamic light scattering (DLS)

Dynamic light scattering (DLS) was performed as previously described (Patel et al., 2017) using purified MEG3 samples in a concentration range from 0.5 μM to 5.5 μM and a Zetasizer Nano S spectrometer (Malvern).

Size-exclusion chromatography coupled to multi-angle laser light scattering (SEC-MALLS)

Size-exclusion chromatography coupled to multi-angle laser light scattering (SEC-MALLS) was performed as described (Folta-Stogniew, 2006; Patel et al., 2017). Purified MEG3 was diluted to concentrations of 0.32-5.0 μM and injected on SEC-MALLS using Tricorn columns (GE Healthcare) self-packed with Sephacryl S500 resin and run in 0.1 M KCl, 8 mM K-MOPS pH 6.5, 0.1 mM Na-EDTA.

Size-exclusion chromatography coupled to small-angle X-ray scattering (SEC-SAXS)

Size exclusion chromatography coupled to small angle X-ray scattering (SEC-SAXS) was performed as described (Chen and Pollack, 2016; Jacques and Trewhella, 2010). Purified MEG3 was filtered using centrifugal filter units with 0.22 μm pore size (Merck Millipore). Different aliquots of pure MEG3 were diluted to concentrations of 0.32-5.0 μM and injected on Tricorn columns (GE Healthcare) self-packed with Sephacryl S500 resin and run in 0.1 M KCl, 8 mM K-MOPS pH 6.5, 0.1 mM Na-EDTA. SAXS data were collected during elution at the BioSAXS beamline BM29 at ESRF, Grenoble and analyzed in ISpyB (Delagenière et al., 2011) and using ATSAS modules PRIMUS and DAMMIF (Petoukhov et al., 2012).

In vitro secondary structure probing (*in vitro* SHAPE)

Selective 2′-Hydroxyl acylation Analyzed by Primer Extension (SHAPE) (Wilkinson et al., 2006) was performed on the peak fraction of MEG3 eluted from SEC and supplemented with 17.5 mM $MgCl_2$. MEG3 was chemically probed using 1-methyl-7-nitroisatoic anhydride (1M7), N-methylisatoic anhydride (NMIA), 1-methyl-6-nitroisatoic anhydride (1M6) and dimethyl sulfate (DMS) for each reagent in triplicate (Rice et al., 2014; Somarowthu et al., 2015).

Modifications were then mapped onto the MEG3 sequence by reverse transcription. 8 primers positioned every 200 bp of MEG3, were designed and coupled with fluorescent dyes 5-FAM and JOE (Tebu-bio). The primer extension reaction was performed using the Omniscript reverse transcriptase (QIAGEN). DMSO and EtOH were used as non-adduct forming controls. Samples were then submitted for fragment length analysis with capillary electrophoresis (Eurofins). QuShape (Karabiber et al., 2013) was used to determine the chemical probing reactivity profiles. Formation of adducts was quantified by comparison between the 1M7-, 1M6-, NMIA- and the DMSO-treated samples and rate of methylation was quantified by comparison between the DMS- and EtOH-treated samples. Average values of individual DMS reactivity values from 3 replicas were self-normalized as described (Chillón et al., 2015). Average values of individual 1M7 reactivity values from 3 experiments were normalized with "simple2boxplot.py" python script (Rice et al., 2014) and average values of individual 1M6 and NMIA reactivity values from 3 experiments were normalized with "boxplot2simple.py" python script (Rice et al., 2014). Such normalization processes also remove outliers, i.e., data points for nucleotides with exceptionally high reactivity values (Rice et al., 2014). Typical reads from consecutive primers overlapped by about ~20 nucleotides. In these overlapping regions, we averaged reactivity values from the two contributing primers in each replica before averaging the corresponding values of independent replicas. Fluctuations in those overlapping regions are similar to fluctuations across experimental biological replicas. Normalized 1M6 reactivity values were subtracted from the NMIA reactivity values with "differenceByWindow.py" python script (Rice et al., 2014). Normalized 1M7 reactivity values were classified in 3 groups as follow: 0-0.40 not reactive (most likely base-paired), 0.40-0.85 moderately reactive and > 0.85 very reactive (most likely single stranded). The software SuperFold with default settings (Siegfried et al., 2014) was used to obtain the secondary structure maps. The software RNAStructure (Mathews, 2004; Reuter and Mathews, 2010) was additionally employed using 1M7 reactivity values of the entire v1 to produce the structural ensemble of the MEG3 core (D2-D3) (Figure S4). Java applet VARNA (Darty et al., 2009) was used to visualize and draw the resulting secondary structures.

In vivo secondary structure probing (*in vivo* SHAPE)

In vivo probing was performed in duplicate on endogenous MEG3 from WI38 or transfected v1 in HCT116 cells. Live cells were collected with cell scraper, pelleted, washed with PBS and supplemented with 900 μL of fresh growth media and with 100 μL of 100 mM or 250 mM 1M7 in DMSO (10x final concentration) as indicated. Negative control samples were treated with DMSO only. Cells were then incubated for 5 minutes at 37°C. Media was removed and the cells were washed once with PBS before isolation of total RNA with RNeasy Mini kit (QIAGEN), according to manufacturer's instructions. DNA was additionally digested with Turbo DNase I (Thermo Scientific) for 1 h at 37°C. Total RNA extract was then cleaned using the Zymogen RNA clean and concentrator kit (Zymo Research), according to manufacturer's instructions. The integrity of extracted RNA was checked with RNA 6000 Nano chips (Agilent) on Agilent 2100 Bioanalyzer. RNA was reverse transcribed to cDNA with random nonamers (NEB), using SuperScript II RT (Invitrogen) in MaP buffer (125 mM Tris-HCl pH 8.0, 187.5 mM KCl, 25 mM DTT, 1.25 mM dNTP, 15 mM $MnCl_2$) that introduces mutations at the sites where 1M7 forms adducts with RNA. As a control a parallel reaction was performed without reverse transcriptase. PCR products were cleaned with PCR clean up kit QIAquick (QIAGEN), according to manufacturer's instructions. Residual RNA was digested with RNase A (Sigma). cDNA was amplified with 4 sets of primers (5′-CGGAGAGCAGAGAGGG-3′ & 5′-GGGTGATGACAGAGTCAGTC-3′; 5′-CCTGACCTTTGCTATGCTC-3′ & 5′- CTGATGCAAGGAGAGCC-3′; 5′-CAGGATCTGGCATAGAGGAG-3′ & 5′-GAATAGGTGCAGGGTGTC-3′; 5′-CCTCTCGTCTCCTTCCTG-3′ & 5′-CAGGAAACACATTTATTGAGAGC-3′) with Q5 hot start

DNA Polymerase (NEB), according to manufacturer's instructions. PCR reactions were cleaned with DNA clean&concentrate^{TM}-5 kit (Zymogen), according to manufacturer's instructions. Concentration of all fragments was measured with Qubit3 fluorimeter (Invitrogen). Size and purity of DNA fragments were checked by high sensitivity DNA chips (Agilent) on Agilent 2100 Bioanalyzer, according to manufacturer's instructions. All fragments belonging to same samples were mixed to 0.2 ng/μl. Libraries were tagmented and amplified by Nextera XT DNA library prep kit (Illumina), according to manufacturer's instructions. Libraries were cleaned with AMPure XP beads (Beckman Coulter), according to manufacturer's instructions. Library concentration was checked with Qubit3 fluorimeter (Invitrogen) and size distribution by high sensitivity DNA chips (Agilent) on Agilent 2100 Bioanalyzer. Libraries were sent for sequencing to the EMBL GeneCore Facility (EMBL Heidelberg). Data were processed with ShapeMapper2 (Busan and Weeks, 2018). 1M7 reactivity values from *in vivo* probing were normalized and scaled following the processing pipeline previously used for lncRNA XIST (Smola et al., 2016). Raw sequencing data are available at BioProject: PRJNA552583.

Ex vivo secondary structure probing (*ex vivo* SHAPE)

Ex vivo probing was performed in duplicate on endogenous MEG3 from WI38 or transfected v1 in HCT116 cells. RNA was extracted using a gentle procedure to avoid denaturation and preserve native secondary structure, following previously established protocol (Smola et al., 2015a). Briefly, live cells were collected with cell scraper, pelleted, washed with PBS and resuspended in 2.5 mL lysis buffer (40 mM Tris-HCl pH 7.9, 25 mM NaCl, 6 mM $MgCl_2$, 1 mM $CaCl_2$, 256 mM sucrose, 0.5% Triton X-100, 1 U/ml murine RNase inhibitor, 100 U/ml turbo DNase) and shaken on ice for 5 minutes. Cells were then pelleted at 4°C for 2 minutes at 1000 *g* and resuspended in 300 μl of resuspension buffer (40 mM Tris-HCl pH 7.9, 200 mM NaCl, 1.5% SDS, and 500 μg/ml of proteinase K) and shaken at room temperature for 45 minutes. RNA was then extracted twice with a mixture of phenol:chloroform:isoamyl alcohol (25:24:1) pre-equilibrated with 1x folding buffer (100 mM HEPES-Na, pH 8.0, 100 mM NaCl, 10 mM $MgCl_2$). Finally, RNA was extracted with chloroform and exchanged into 1.1 × folding buffer using a desalting column (PD-miditrap G25, GE Life Sciences). RNA extracts were incubated at 37°C for 20 minutes. Approximately 10 μg of RNA was then added to a one-ninth volume of 100 mM 1M7 in DMSO (10 mM final concentration) and incubated at 37°C for 5 minutes. Negative control samples were treated with DMSO only. Modified RNA was cleaned using the Zymogen RNA clean and concentrator kit (Zymo Research), according to manufacturer's instructions. The integrity of extracted RNA was checked with RNA 6000 Nano chips (Agilent) on Agilent 2100 Bioanalyzer. RNA was reverse transcribed to cDNA with random nonamers (NEB), and amplified as described for *in vivo* SHAPE probing above. Data were processed with ShapeMapper2 (Busan and Weeks, 2018). 1M7 reactivity values from *ex vivo* probing were normalized and scaled following the processing pipeline previously used for lncRNA XIST (Smola et al., 2016). Such values were then compared to corresponding 1M7 reactivity values from *in vivo* probing using deltaSHAPE (Smola et al., 2015a, 2015b). Raw sequencing data are available at BioProject: PRJNA552583.

Hydroxyl radical footprinting (HRF)

HRF was performed in triplicates for each condition, following a protocol described previously for group II intron (Swisher et al., 2001), with minor modifications. MEG3 was purified as for SHAPE (see above), but in 10 mM potassium cacodylate pH 7.0, 0.1 mM EDTA, 150 mM KCl to prevent quenching of radicals. 10 pmol of purified RNA were then supplemented with DEPC-treated water or 17.5 mM $MgCl_2$ in the purification buffer and folded at 37°C for 45 min. Folded RNA was subjected to Fenton reaction by treatment with iron-EDTA solution (4 mM iron (II) sulfate hexahydrate: 4.4 mM Na-EDTA pH 8) to a final concentration of 0.08:0.088 mM, sodium ascorbate to a final concentration of 1 mM and H_2O_2 to a final 0.6% Vol. All solutions used in the Fenton reaction step were prepared freshly directly prior to use. The RNA was kept at 37°C at all times to maintain homogeneous folding. The reagents were deposited in equal volumes on the walls of the Eppendorf tubes containing the folded RNA and mixed with the sample simultaneously by brief vortexing. Control samples were treated with an equal total volume of DEPC-treated water. Samples were incubated at 37°C for 15 s and the reaction was stopped by the addition of quenching solution (100 mM thiourea, 200 mM EDTA, pH 8.0) and samples were transferred on ice. RNA was isolated by isopropanol precipitation. Treated RNA was resuspended in 50 μL RNA storage buffer (10 mM K-MOPS pH 6.5, 0.1 mM Na-EDTA pH 8.0) and analyzed by fragment extension and fragment length analysis as for SHAPE. Reactivity values of v1 (compact state) from 3 independent experiments were normalized to the corresponding water-treated control sample using the "simple2boxplot.py" python script (Rice et al., 2014). Such normalization processes also remove outliers, i.e., data points for nucleotides with exceptionally high reactivity values (Rice et al., 2014). Normalized HRF reactivity values were then classified into 4 groups, for color coding of Figure 5B: 0-0.29 not reactive (most solvent-protected), 0.29-0.58 poorly reactive (moderately solvent-protected), 0.58-0.86 moderately reactive (moderately solvent-exposed), and > 0.86 very reactive (most solvent-exposed). Reactivity values of H11LpA were scaled to the reactivity values of v1 (compact state) using the normalization procedure of QuSHAPE, as described (Karabiber et al., 2013).

Atomic force microscopy (AFM)

MEG3 and group II intron (Marcia and Pyle, 2012) were purified as described and the fraction of MEG3 and group II intron with the highest concentration eluted from SEC after purification was diluted in filtration buffer (0.1 M KCl, 8 mM K-MOPS pH 6.5, 0.1 mM Na-EDTA), filtration buffer with magnesium (0.1 M KCl, 8 mM K-MOPS pH 6.5, 0.1 mM Na-EDTA, 10mM $MgCl_2$), or water at the desired concentrations. Poly(A) RNA (GE Healthcare) used as negative control was dissolved in the same buffers at a concentration of 0.3 μg/ml. To obtain denatured samples, the corresponding RNAs were precipitated with isopropanol overnight at −20°C and

resuspended in deionized formamide and diluted with ethanol to reach same final concentration as samples diluted in buffer. A 1 μl, 2.5 μl or 5 μl drop of RNA was deposited on freshly cleaved mica, incubated for 3 min, washed with 2 mL of water with 200 μl drop steps to remove excessive salt crystals, and finally dried with nitrogen gas. Denatured samples were deposited on mica, incubated 3 min and dried with nitrogen gas. Imaging was performed on a Multimode 8, Nanoscope V (Bruker) equipped with NanoScope software (Bruker, Santa Barbara, CA). Imaging was done with peak force tapping (PFT) imaging mode at ~1Hz rate, with 512 or 1024 pixel sampling and other PFT parameters were initially manually adjusted and then automatically controlled with ScanAsyst mode in air. Cantilever ScanAsyst-air (Bruker) with a nominal 2 nm tip radius, 70 kHz frequency and 0.4 N/m spring constant was used. Images were processed with Gwyddion (Necas and Klapetek, 2012), and if needed stripe noise was removed using DeStripe (Chen and Pellequer, 2011). Power Spectral Densities (PSDs) of the AFM topographic signal were collected in square regions of 250 nm side around each particle of interest (Higuchi, 1988). PSD plots were computed separately for all particles acquired at 1024x1024 pixel^2 with a pixel size of 0.98 nm/pixel (reported in Figure 6) and for all particles acquired at 512x512 pixel^2 with a pixel size of 1.96 nm/pixel (nearly identical to the 1 nm/pixel PSDs). The PSDs were collected along the fast scanning axis of the microscope to avoid potential artifacts due to line-to-line offset. For comparing v1 and H11LpA (Figure 6), the PSDs was collected along the y axis, due to a slight resonance of the tip along the fast scanning axis (x) of the mutant dataset. PSDs of v1 along x and y are nearly identical. PSDs were calculated using the SPM data analysis software Gwyddion (Necas and Klapetek, 2012), and the PSDs for all the particles observed under each given experimental condition were averaged using the software Igor Pro (WaveMetrics, USA). The resulting averaged PSDs were plotted against the spatial (angular) frequency and the associated spatial length scale (Calò et al., 2009). Linear fits to the so-called auto-affine region, i.e., where the PSD frequency dependence is of the form $PSD(f) = a_0 f^{-\gamma}$, are displayed as dashed lines. Fits were performed using a weighted least square algorithm within an arbitrarily selected x-range using the software Igor Pro. In total we acquired 10 images (75 particles) for v1 in the denatured state, 20 images (109 particles) for v1 in the intermediate state, 15 images (108 particles) for v1 in the compact state, and 21 images (106 particles) for H11LpA in K^+ and Mg^{2+}.

Quantitative real-time PCR (qRT-PCR)

cDNA was generated from 5 μg total RNA by reverse transcription (RT) using random hexamers (Thermo) and SuperScript II reverse transcriptase (Invitrogen). qRT-PCR was performed on a Mx3005P qPCR system (Agilent) and data were analyzed using the Pfaffl method (Pfaffl, 2001). The program was comprised of 40 amplification cycles using an annealing temperature of 62°C for 30 s and an elongation time of 30 s at 72°C, followed by the generation of a melting curve. Primers were designed with Clone Manager Professional Suite (Sci Ed Central) and examined for possible secondary structures with OligoAnalizer 3.1 (Integrated DNA Technologies). Amplified target regions and corresponding primers are provided in Table S3, respectively. Beta-actin mRNA was used as reference to normalize for total cellular RNA. Neomycin mRNA was used as a reference to normalize for transfection efficiency. Statistical analyses were performed using the Prism 6 package (GraphPad Software).

Western blot

HCT116 cells were transfected in 6-well plates with 1 μg of pcDNA3-MEG3 v1 plasmid or equimolar amounts of the indicated plasmids using 5 μL of Lipofectamine 2000 (Invitrogen). After 48 hours, total cell lysates were prepared from trypsinized cells, pelleted and resuspended in modified buffer A (150 mM KCl, 25 mM Tris-HCl pH 7.4, 1.5 mM $MgCl_2$, 1 mM DTT, 0.5% Igepal, 1 mM PMSF, cOmplete protease inhibitor, 9 μg/ml leupeptin, 9 μg/ml pepstatin, 100 U/ml RNaseOUT). The lysate was then sonicated using a Bioruptor® System (Diagenode) using the following program: 10 cycles (30 s on, 30 s off) at position H, 5 cycles (30 s on, 30 s off) at position M. Finally, the cell debris were eliminated by centrifugation at maximum speed for 10 min and the supernatant was directly used for the experiment. 20 μg or 40 μg of total cell lysate were loaded onto a 4%–12% NuPAGE® Bis-Tris Gels (Invitrogen) with MOPS SDS Running Buffer for 55 min at 200V (actin, p53) or onto a 12% NuPAGE® Bis-Tris Gels (Invitrogen) with MES SDS Running Buffer for 75 min at 200V (BAX, p21). The proteins were transferred to a nitrocellulose membrane using an iBlot 2 Dry Blotting System (Invitrogen), following manufacturer's recommendations. The blots were blocked using UltraCruz® Blocking Reagent (Santa Cruz Biotechnology) for 1 h at room temperature with shaking. The primary and secondary antibodies (Table S4) were diluted in UltraCruz® Blocking Reagent (Santa Cruz Biotechnology), incubated with the membrane for 1 h at room temperature with shaking and washed with TBST buffer 1X (10 mM Tris-Cl pH 8, 150 mM NaCl, 0.05% Tween-20). The blots were visualized under a ChemiDoc MP Imaging System (Bio-Rad) using the appropriate filters.

Luciferase assay

HCT116 cells were seeded at 83000 cells/well in a cell-culture treated 12-well plate (Costar) and transfected after 24 h with 115.96 fmol of pcDNA3 vector containing the indicated MEG3 constructs, 50 ng of p53-Luc [kind gift from Yunli Zhou (Zhou et al., 2007)] and 5 ng of pRL Renilla Luciferase Control Reporter Vector (Promega) using Lipofectamine 2000 (Life Technologies), according to manufacturer's instructions. Transfected cells were incubated for 48 h. Cells were lysed with 1x passive lysis buffer provided in the Dual-Luciferase® Reporter (DLR) Assay System (Promega). Production of the Firefly luciferase was measured by adding Luciferase Assay Reagent II (Promega) and measuring luminescence with microplate reader CLARIOstar (BMG Labtech). This reaction was then quenched and production of Renilla luciferase was measured by adding Stop & Glo® Reagent (Promega) to normalize the Firefly readout values for transfection efficiency. RNA expression was confirmed for all constructs by qRT-PCR.

Cell cycle and apoptosis assays by flow cytometry

HCT116 cells were seeded at 200,000 cells/well in 6-well cell culture plates (Costar) and transfected with 1 μg of pCMS-d2EGFP-MEG3v1 or equimolar amounts of indicated plasmids with 5 μL Lipofectamine 2000 (Invitrogen). For cell cycle analysis, cells were incubated with EdU and the incorporation was detected using the Click-iT Plus EdU Flow Cytometry Assay Kit (Thermo Fisher Scientific) following manufacturer's instructions with some modifications. Briefly, at each time point, cells were labeled with 10 μM EdU for 1 h, washed with PBS and trypsinized. The trypsinized cells were washed with PBS and incubated with LIVE/DEAD Fixable Dead Cell Stain (Invitrogen) for 15 min to evaluate the viability of the transfected cells. Cells were then fixed with a 4% paraformaldehyde solution for 15 min and permeabilized with Click-iT saponin-based permeabilization and wash reagent. After all time points were collected, samples were subjected to the Click-iT reaction following manufacturer's instructions and resuspended in Click-iT® saponin-based permeabilization and wash reagent containing 1 μL FxCycle Violet Stain (Invitrogen). For the apoptosis assay, 100,000 cells were incubated with 5 μl of Brilliant Violet 421 annexin V (Biolegend) and 10 μl of 0.5 mg/ml of propidium iodide (Biolegend). Compensation controls were prepared from samples stained with one dye at a time. Data were acquired on a BD LSR II Flow Cytometer (Becton Dickinson) and on a MACSQuant® VYB instrument (Miltenyi Biotec) and analyzed using the FCS Express 6 package (*De Novo* Software).

RNA immunoprecipitation (RIP)

RIP was performed following the protocol established by Keene et al., with some modifications (Keene et al., 2006). Briefly, 2×10^7 HCT116 cells were lysed in lysis buffer (10 mM HEPES pH 7.4, 100 mM KCl, 5 mM $MgCl_2$, 0.5% NP40, 1 mM DTT plus RNase and proteinase inhibitors) for 3 h at −80°C and centrifuged at 12,000 × *g* for 30 min at 4°C. The supernatants were collected and 1% of each sample was set aside as input while the remaining was incubated for 4 h at 4°C with protein G magnetic beads (Thermo Fisher Scientific) coated either with 4 μg of an anti-p53 antibody (Santa Cruz, DO1) or with 4 μg of mouse IgG (Santa Cruz). The beads were then washed four times with NT2 buffer (50 mM Tris–HCl pH 7.5, 150 mM NaCl, 1 mM $MgCl_2$, 0.05% NP-40, 0.5% urea) and RNA was isolated from Input and IP samples using TRIzol (Thermo Fisher Scientific). Extracted RNAs were treated with DNase for 30 min at 37°C prior to being converted to cDNA using the RevertAid First Strand cDNA Synthesis Kit (Thermo Fisher Scientific), following manufacturer's instructions. The qPCRBIO SyGreen Mix (PCR Biosystems) was used as master mix for qRT-PCR, adding 200 nM forward and reverse primers. The reactions were performed on a CFX384 thermal cycler (Biorad) in technical duplicates for each target and for a total of four biological replicas. Raw data were processed with Biorad CFX Manager software to obtain the Ct values. Results are expressed as percent of input. GAPDH and RNR1 were used as controls for p53 non-specific RNA binding.

Pull-down assays

In vitro transcribed biotinylated v1 and H11LpA were produced using the biotin RNA labeling mix (Roche) with 2.5 μg of linearized plasmids in a final volume of 50 μL and purified under non-denaturing conditions (Chillón et al., 2015) into equilibration buffer (150 mM KCl, 10 mM Tris-HCl pH 7.4, 1.5 mM $MgCl_2$). The integrity of the RNA was assessed in a 2100 Bioanalyzer System (Agilent Technologies). The HCT116 cells were grown in 10-cm plates without antibiotics and treated with (+/−)-nutlin-3 (Cayman Chemical) at 10 μM final concentration for 16 h. The nuclei were extracted by incubating the cells in a solution containing 3:1:1 of water, PBS and nuclear isolation buffer (1.28 M sucrose, 40 mM Tris-HCl pH7.5, 20 mM MgCl2 and 4% Triton X-100) (Marín-Béjar and Huarte, 2015), respectively, for 20 minutes at 4°C with constant mixing. The nuclei were spun at 300x *g* for 15 minutes at 4°C and the pellet dissolved in modified buffer A (150 mM KCl, 25 mM Tris-HCl pH 7.4, 1.5 mM $MgCl_2$, 1 mM DTT, 0.5% Igepal, 1 mM PMSF, cOmplete protease inhibitor, 9 μg/ml leupeptin, 9 μg/ml pepstatin, 100 U/ml RNaseOUT). The nuclei suspension was sonicated using a Bioruptor® System (Diagenode) using the following program: 15 cycles (30 s on, 30 s off) at position H, 5 cycles (30 s on, 30 s off) at position M. Finally, the cell debris were eliminated by centrifugation at maximum speed for 10 minutes and the supernatant was directly used for the experiment. The nuclear lysate was pre-cleared by incubating it with 0.25 mg/mg protein of yeast tRNA and 1 mg /mg protein of M-280 beads (Invitrogen), previously cleaned following manufacturer's instructions, for 1 h at 4°C under rotation (Zhou et al., 2017). The beads were coated with BSA and tRNAs to prevent unspecific interactions by incubating them in binding buffer (0.2 mg/ml BSA ultrapure and 50 μg/ml yeast tRNA in modified buffer A) for 2 h at 4°C under rotation. The pull-down was performed by mixing 0.5 mg of pre-cleared nuclear lysate with 10 μg of biotinylated RNA (20 pmol) for 2 h at 4°C with constant mixing, followed by addition of 450 μg of washed and coated M-280 beads, for 30 minutes at room temperature. The beads were washed five times with 0.5 mL of modified buffer A and eluted in 25 μL of Laemmli sample buffer before loading the samples onto a 4%–12% NuPAGE® Bis-Tris Gels (Invitrogen) with MOPS SDS Running Buffer. The gel was blotted as described in the western blot section above.

Sequence and structural alignments

Sequences corresponding to human MEG3 exons (nucleotides 230-902) were identified in other mammals with BLAT (Kent, 2002) and aligned in Clustal Omega (Li et al., 2015). We defined mammalian orders according to (Tarver et al., 2016). For secondary structure-based alignments, Clustal Omega was used to align 19 mammal sequences corresponding to human MEG3 E3 and selected to cover at least 2-3 species for each order of mammals and limiting overrepresentation of any order, especially primates. A covariance model was then built, calibrated, and used to expand such seed alignment to a total of 41 sequences in Infernal (Nawrocki and Eddy, 2013), based on the secondary structure for D2-D3 of human v1. R2R (Weinberg and Breaker, 2011) was used to graphically depict the resulting alignment files produced by Infernal. Additionally, we used RScape (Rivas et al., 2017) to assess the statistical

significance of observed covariations. Default settings of RScape do not reveal significance covariation, but a windowing approach using RAFS covariation metrics (Tavares et al., 2019) determines the following base pairs as significantly covariant (E values < 0.05): 241-292 and 242-291 (H8); 308-339, 309-338 and 310-337 (H10); 359-380 and 361-378 (H11); 499-516 (H17); 540-551 (H19); 573-686 (H21); 601-613 and 602-612 (H22); and 808-823, 810-821, and 812-819 (H28) (numbers from human v1, see Figure 2). RScape does not detect significant covariance in the proposed H11-H27 pseudoknot. Statistical assessment of the significance of covariation in lncRNAs is controversial and must be interpreted with caution (Rivas et al., 2017; Somarowthu et al., 2015; Tavares et al., 2019) particularly when the number of aligned sequences is small, as in the case of MEG3 (41 sequences).

QUANTIFICATION AND STATISTICAL ANALYSIS

All statistical analyses were performed in Prism v.6.05 (GraphPad Software Inc) using one-way ANOVA or unpaired parametric t tests, as indicated in the respective Figure legends. Figure legends also report the respective values of independent experiments (n), definition of the center, and dispersion measures.

DATA AND CODE AVAILABILITY

The accession number for the RNA sequencing data for *in vivo* and *ex vivo* SHAPE probing reported in this paper is BioProject: PRJNA552583. Original data for Figure 1A, Figures 4E, 4F, and 4H, and Figures 6 and S7 are available in Mendeley (Mendeley Data https://doi.org/10.17632/xcc3x848rv.1). All other data are available from the corresponding authors on request.

Molecular Cell, Volume *75*

Supplemental Information

Conserved Pseudoknots in lncRNA MEG3 Are Essential for Stimulation of the p53 Pathway

Tina Uroda, Eleni Anastasakou, Annalisa Rossi, Jean-Marie Teulon, Jean-Luc Pellequer, Paolo Annibale, Ombeline Pessey, Alberto Inga, Isabel Chillón, and Marco Marcia

SUPPLEMENTAL INFORMATION FILE FOR:

Conserved pseudoknots in lncRNA MEG3 are essential for stimulation of the p53 pathway

Tina Uroda[1], Eleni Anastasakou[1], Annalisa Rossi[2], Jean-Marie Teulon[3], Jean-Luc Pellequer[3], Paolo Annibale[4], Ombeline Pessey[1], Alberto Inga[2], Isabel Chillón[1,*], Marco Marcia[1,*]

[*]To whom correspondence should be addressed. E-mail: ichillon@embl.fr; mmarcia@embl.fr

LEAD AUTHOR: Marco Marcia, mmarcia@embl.fr

The supplemental information file contains:

- Fig. S1 (Related to Fig. 1)
- Fig. S2 (Related to Fig. 1)
- Fig. S3 (Related to Fig. 1)
- Fig. S4 (Related to Fig. 1)
- Fig. S5 (Related to Fig. 3, 4 and 5)
- Fig. S6 (Related to Fig. 5)
- Fig. S7 (Related to Fig. 6)
- Table S1 (Related to Fig. 1, 5 and 6)
- Table S2 (Related to Fig. 2)
- Table S3 (Related to Fig. 4)
- Table S4 (Related to Fig. 4)
- Data File 1 (Related to Fig. 2)

SUPPLEMENTAL FIGURES

Fig. S1: *In vitro* SHAPE structure maps of v1, v3, and v9 (Related to Fig. 1). **A)** *In vitro* secondary structure of v1 color-coded based on the 1M7 and DMS reactivity values of individual nucleotides. Black boxes indicate exon junctions. Helices (H) and multi-way junctions (J) are numbered consecutively from the 5' to the 3'-end. **B-C)** *In vitro* secondary structure representations of v3 and v9, respectively, colored as in panel A (1M7 reactivity values only). Helices and junctions that are different with respect to v1 are assigned a number followed by the variant name (i.e. H1v3).

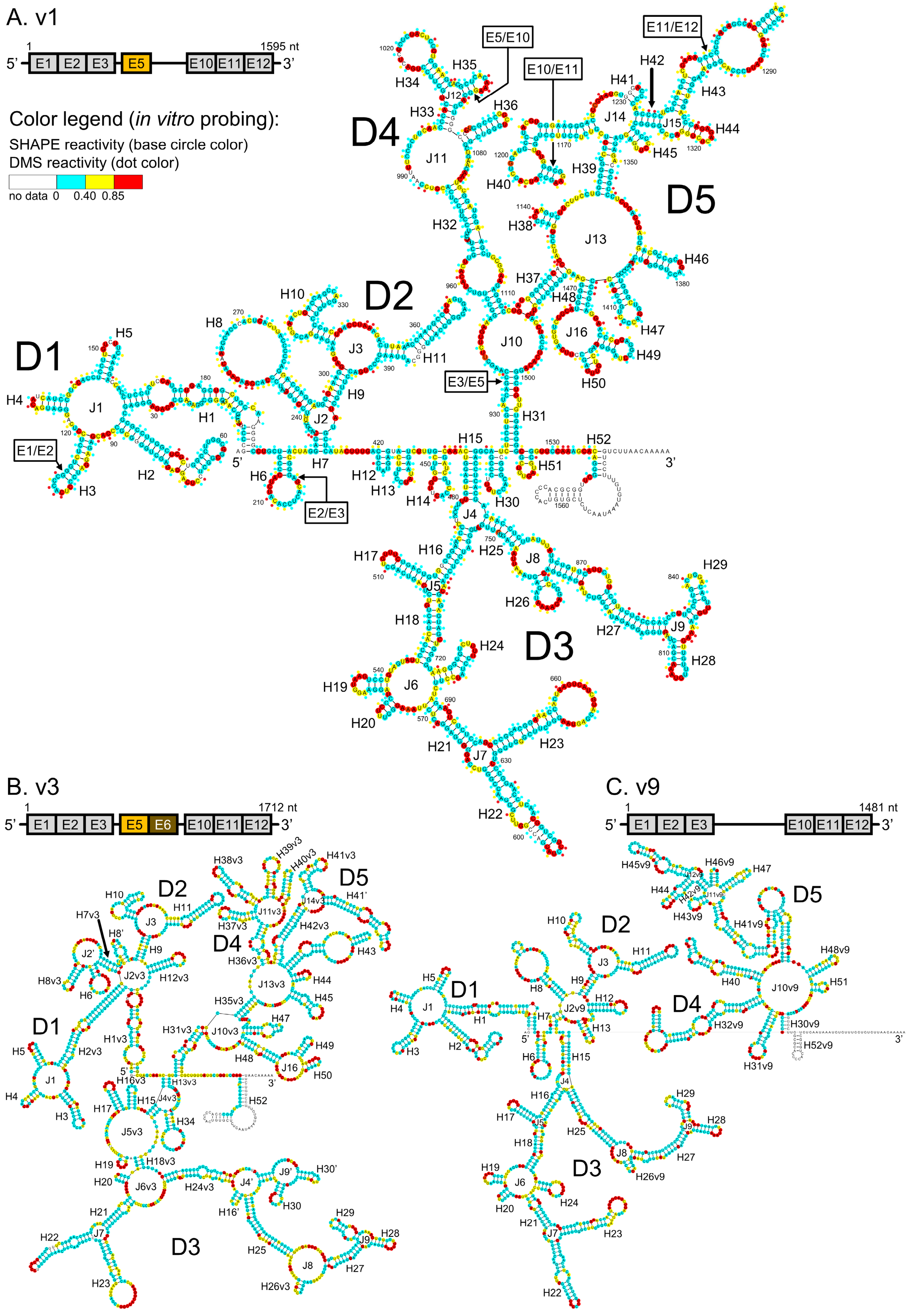
A. v1
1
1595 nt
5'
E1 E2 E3 E5 E10 E11 E12
3'
Color legend (in vitro probing):
SHAPE reactivity (base circle color)
DMS reactivity (dot color)
no data 0 0.40 0.85
D1
D2
D3
D4
D5
E1/E2
E2/E3
E3/E5
E5/E10
E10/E11
E11/E12
B. v3
1
1712 nt
5'
E1 E2 E3 E5 E6 E10 E11 E12
3'
C. v9
1
1481 nt
5'
E1 E2 E3 E10 E11 E12
3'

Fig. S2: *In vitro* SHAPE probing of v1, v3, and v9 (Related to Fig. 1). **A-C)** *In vitro* SHAPE structures of v1, v3, and v9. From top to bottom, for each variant: exon and domain architecture; 1M7 and Shannon entropy reactivity profile (we note that Shannon entropy calculations in Superfold use 50-nt-sliding windowed medians, not single nucleotide resolution); base-pairing probabilities; and minimum free-energy structures. *In vitro* reactivity values of individual nucleotide of all three variants and corresponding SEM from n=3 experiments are reported in **Figure S3**. Spearman's correlation coefficient (CC_S) between replicas are indicated on the right. **D)** CC_S between splicing variants. **E)** CC_S between replicas of H11LpA. **F)** Average and median Shannon entropy values (S_{ave} and S_{median}, respectively) and median SHAPE reactivity (R_{median}) for each variant.

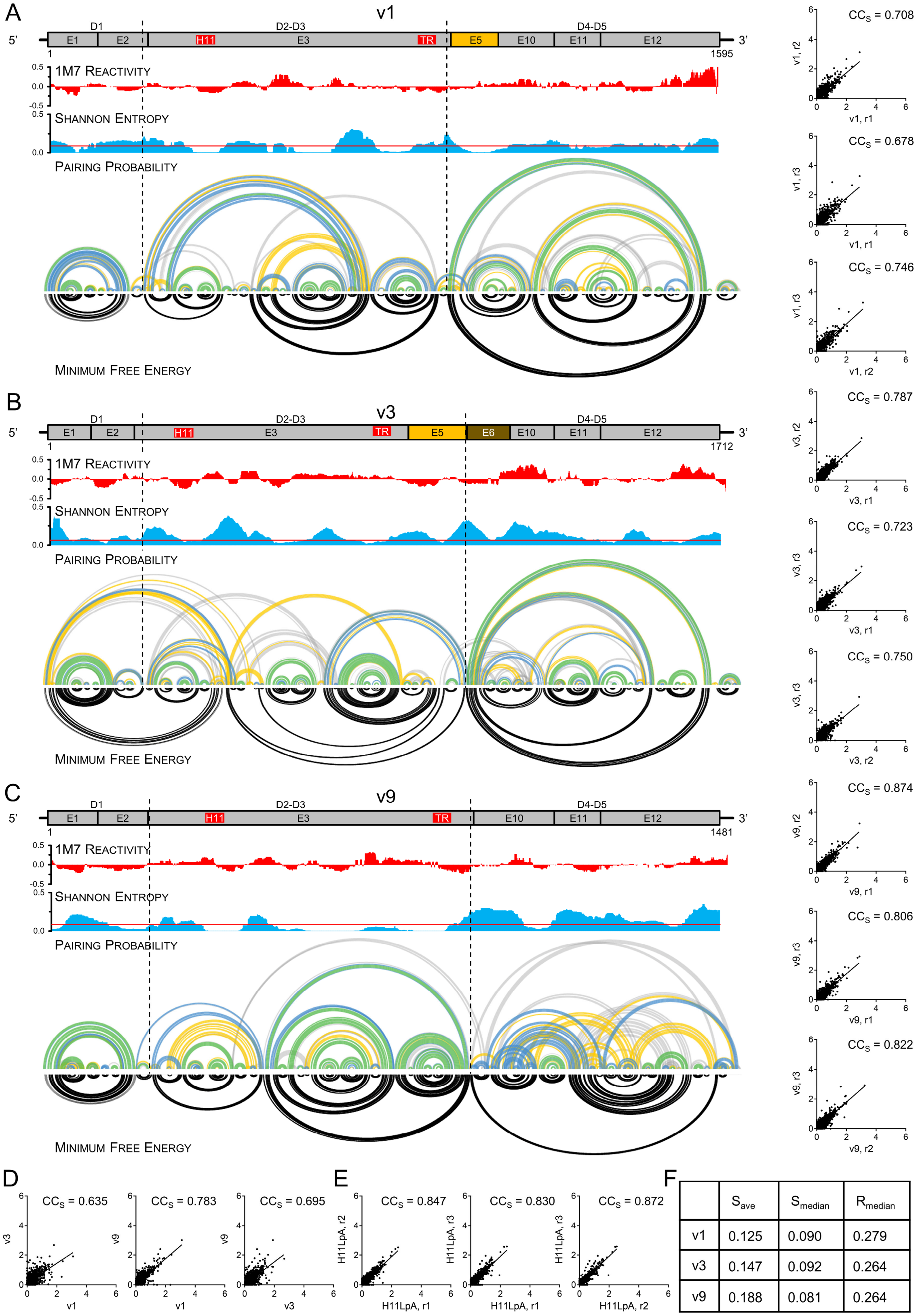

	S_{ave}	S_{median}	R_{median}
v1	0.125	0.090	0.279
v3	0.147	0.092	0.264
v9	0.188	0.081	0.264

Fig S3: *In vitro* SHAPE probing of v1, v3, and v9 (Related to Fig. 1). The graph reports 1M7 reactivity values of individual nucleotides. Error bars indicate SEM from n = 3 experiments. The scale of the y-axis is set to match the color scale of **Figure S1**. Nucleotides with very high reactivity values (> 1.2) are excluded for clarity of visualization. The x-axis reports nucleotide numbers for the longest variant, v3. Black boxes indicate exon junctions, gray labels and arrows indicate domains, and red boxes indicate the position of H11 and TR1-6.

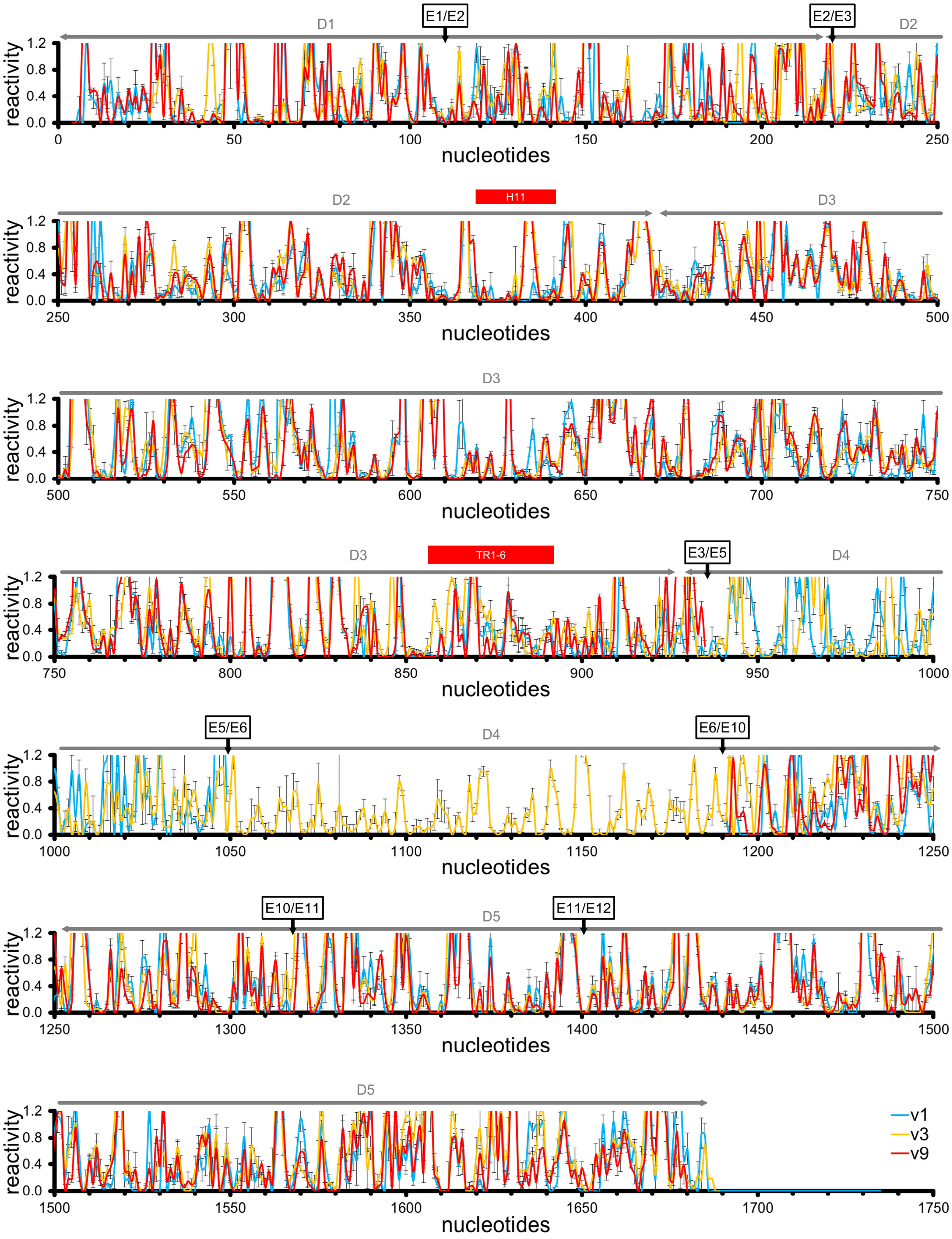
D1
E1/E2
E2/E3
D2
H11
D3
TR1-6
E3/E5
D4
E5/E6
E6/E10
E10/E11
E11/E12
D5
reactivity
nucleotides
v1
v3
v9

Fig S4: Structure ensemble of the MEG3 core (Related to Fig. 1). Ensemble of 9 secondary structure maps of the D2-D3 core produced by RNAStructure using only 1M7 reactivity values of v1.

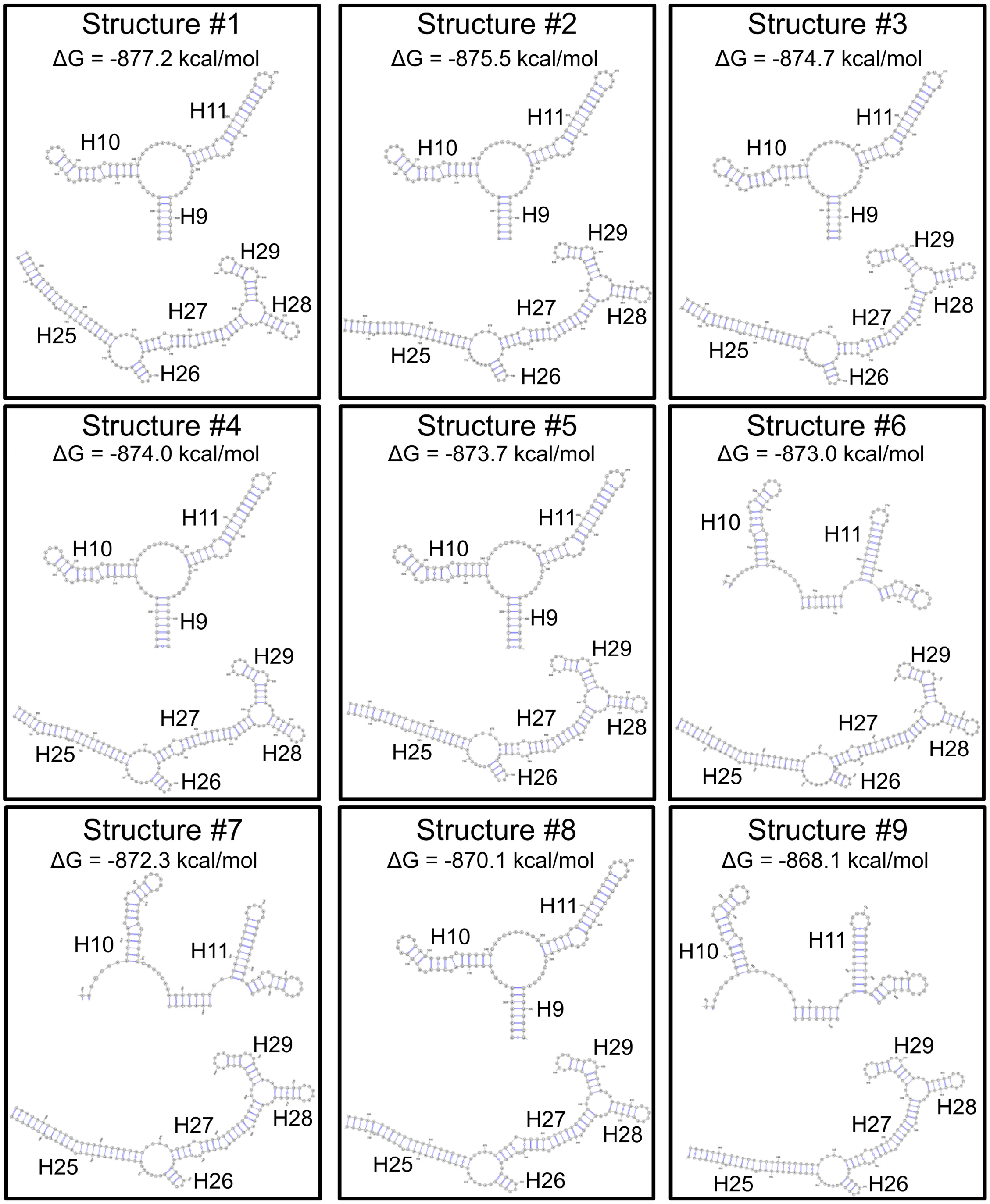

Fig. S5: Effects of MEG3 on cell cycle and transactivation of p53 target genes (Related to Fig. 3, 4 and 5). **A-I)** Distribution of GFP^{-} cell populations for the flow cytometry assays described in **Figure 3**. **J-M)** Optimization of the luciferase assay (all data points are normalized to the signal of corresponding control vectors, set to a y-axis value of 1). DNA titration (J) and time course performed with v1 (K). At 60 h cells are not healthy anymore. Stimulation of the p53 pathway by v1 transcribed from pCI and pcDNA3 vectors (L). Time course in which Firefly luciferase values have been normalized with Renilla luciferase or beta-galactosidase signals as transfection controls (M). **N)** Quantification by qRT-PCR the abundance of endogenous MEG3 relative to actin mRNA (ACTB) in HCT116 and WI38. **O)** Quantification by qRT-PCR of the abundance of all mutants used for luciferase assays. All constructs were transfected in HCT116 cells and expressed under the same conditions used for functional assays. Abundance of mRNA of each MEG3 construct was calculated relative to actin (loading control) and neomycin (transfection control) mRNAs. Values are expressed as fold changes with respect to the abundance of v1. Construct D5 is indicated in parentheses because it is > 10000 fold less expressed than v1. Error bars indicate SEM from n = 3 (panels A-N) or n = 2 experiments (panel O).

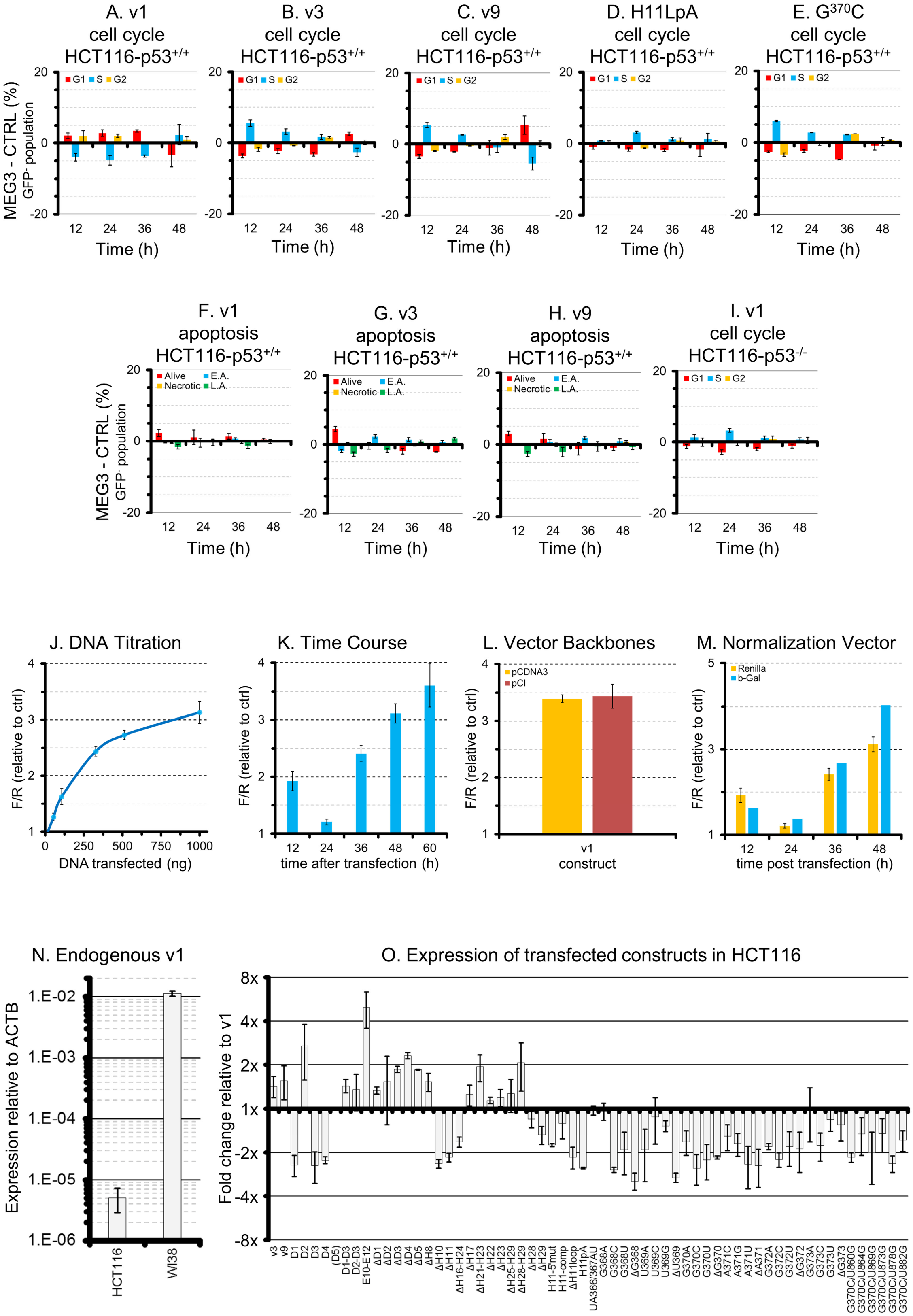

A. v1
cell cycle
HCT116-p53+/+
B. v3
cell cycle
HCT116-p53+/+
C. v9
cell cycle
HCT116-p53+/+
D. H11LpA
cell cycle
HCT116-p53+/+
E. G370C
cell cycle
HCT116-p53+/+
G1 S G2
MEG3 - CTRL (%)
GFP- population
20
0
-20
12 24 36 48
Time (h)
F. v1
apoptosis
HCT116-p53+/+
G. v3
apoptosis
HCT116-p53+/+
H. v9
apoptosis
HCT116-p53+/+
I. v1
cell cycle
HCT116-p53-/-
Alive
E.A.
Necrotic
L.A.
J. DNA Titration
F/R (relative to ctrl)
DNA transfected (ng)
0 250 500 750 1000
K. Time Course
time after transfection (h)
12 24 36 48 60
L. Vector Backbones
pCDNA3
pCI
v1
construct
M. Normalization Vector
Renilla
b-Gal
time post transfection (h)
N. Endogenous v1
Expression relative to ACTB
1.E-02
1.E-03
1.E-04
1.E-05
1.E-06
HCT116
WI38
O. Expression of transfected constructs in HCT116
Fold change relative to v1
8x
4x
2x
1x
-2x
-4x
-8x
v3 v9 D1 D2 D3 D4 (D5) D1-D3 D2-D3 E10-E12 ΔD1 ΔD2 ΔD3 ΔD4 ΔD5 ΔH8 ΔH10 ΔH11 ΔH16-H24 ΔH17 ΔH21-H23 ΔH22 ΔH23 ΔH25-H29 ΔH28-H29 ΔH28 ΔH29 H11-5'mut H11-comp ΔH11loop H11pA UA366/367AU G368A G368C G368U ΔG368 U369A U369C U369G ΔU369 G370A G370C G370U ΔG370 A371C A371G A371U ΔA371 G372A G372C G372U ΔG372 G373A G373C G373U ΔG373 G370C/U860G G370C/U864G G370C/U869G G370C/U873G G370C/U878G G370C/U882G

Fig. S6: HRF probing of v1 and H11LpA (Related to Fig. 5). **A)** HRF reactivity values of individual nucleotides of v1 and H11LpA. Error bars indicate SEM from n = 3 experiments. The scale of the y-axis is set to match the color scale of **Figure 5C**. Nucleotides with very high reactivity values (> 1.2) are excluded for clarity of visualization. Black boxes indicate exon junctions. **B)** $CC_{Spearman}$ between HRF replicas of v1 and H11LpA (r1-r3). **C)** Comparison of number of secondary structure motifs (top) and solvent-protected nucleotides (bottom) in v1 *vs* other indicated structured RNAs. The number of secondary structure motifs of each RNA has been normalized to the size (number of nucleotides) of MEG3 v1.

A. HRF, raw reactivity values

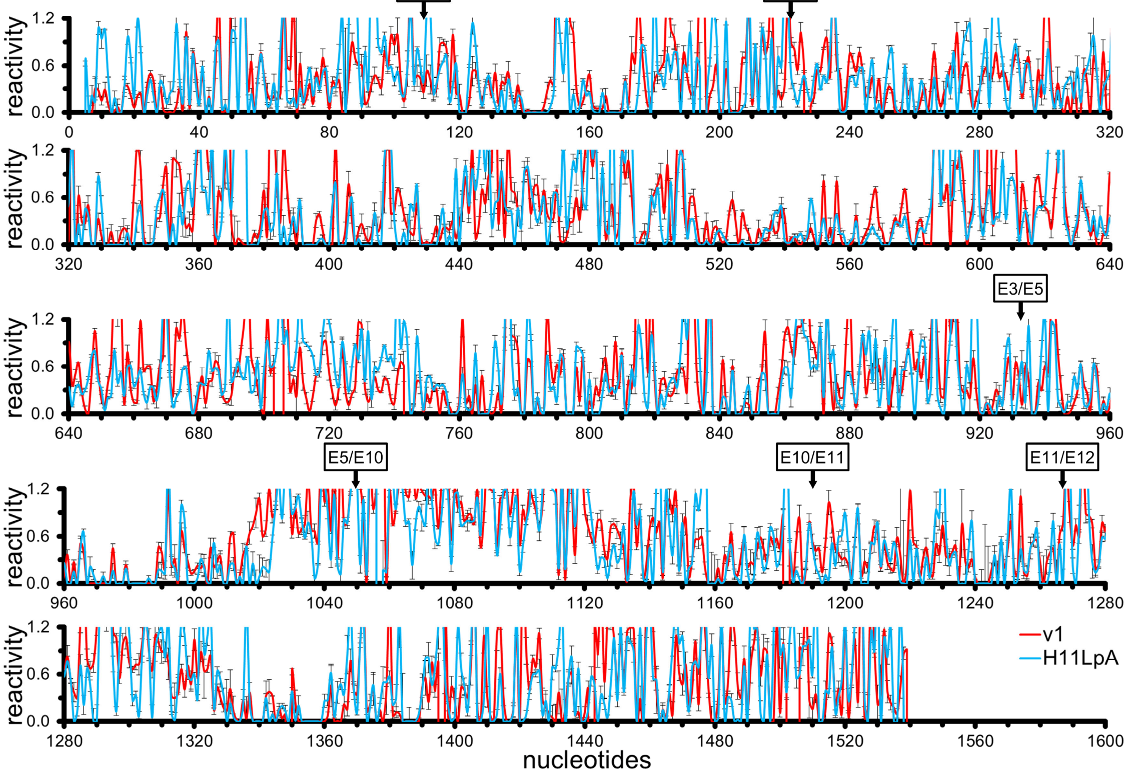

B. HRF, correlation plots

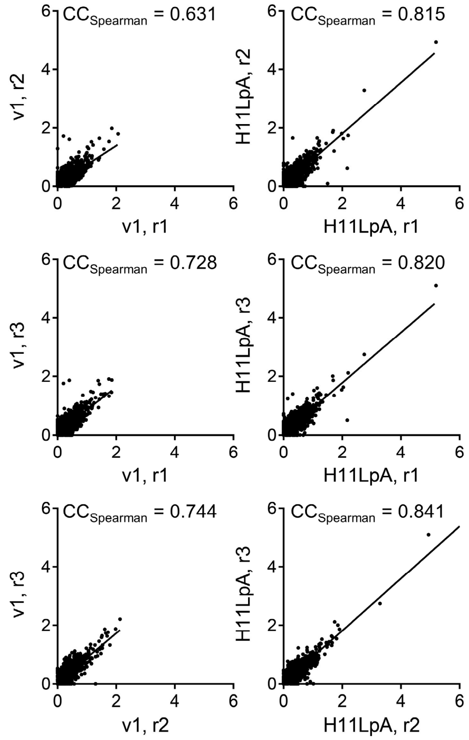

C. Comparison with other structured RNAs

	v1	HOTAIR	SRA	BRAVEHEART	COOLAIR	RepA
	this work	Somarowthu et al, 2015	Novikova et al, 2012	Xue et al, 2016	Hawkes et al, 2016	Liu et al, 2017
Size (nt)	1595	2148	873	590	658	1630
Stem loops	51	28[1]	31[1]	32[1]	29[1]	38[1]
Multiway junctions	16	13[1]	11[1]	3[1]	7[1]	11[1]
Internal loops	40	51[1]	53[1]	24[1]	39[1]	34[1]

[1]: proportional to the size of MEG3

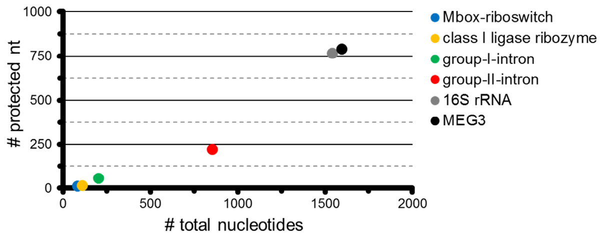

Fig S7: AFM analysis of v1 and H11LpA (Related to Fig. 6). **A)** Atomic force microscopy images of v1 and H11LpA. Top left: large view field of a sample of v1 in the compact (K^+ and Mg^{2+}) state, representative of the homogeneity of the sample on the mica support. Top right and bottom: Collection of 100 single particles of v1 in the compact (K^+ and Mg^{2+}, top right) and intermediate (K^+ only, bottom left) states, and of H11LpA in K^+ and Mg^{2+} (bottom right). The xy scale bar is indicated by a white line at the bottom of each panel. The z color scale bar for all AFM panels is indicated on the top right. **B)** SEC-SAXS of v1 in intermediate folding state (K^+ only), used for calculation of R_g and D_{max} (**Table S1**). Left: Scattering curve. Middle: Guinier plot. Right: P(r) plot. **C)** AUC titration of v1 at different magnesium concentrations, used for calculation of R_h, $C_{Mg1/2}$ and n_{Hill} (**Table S1**).

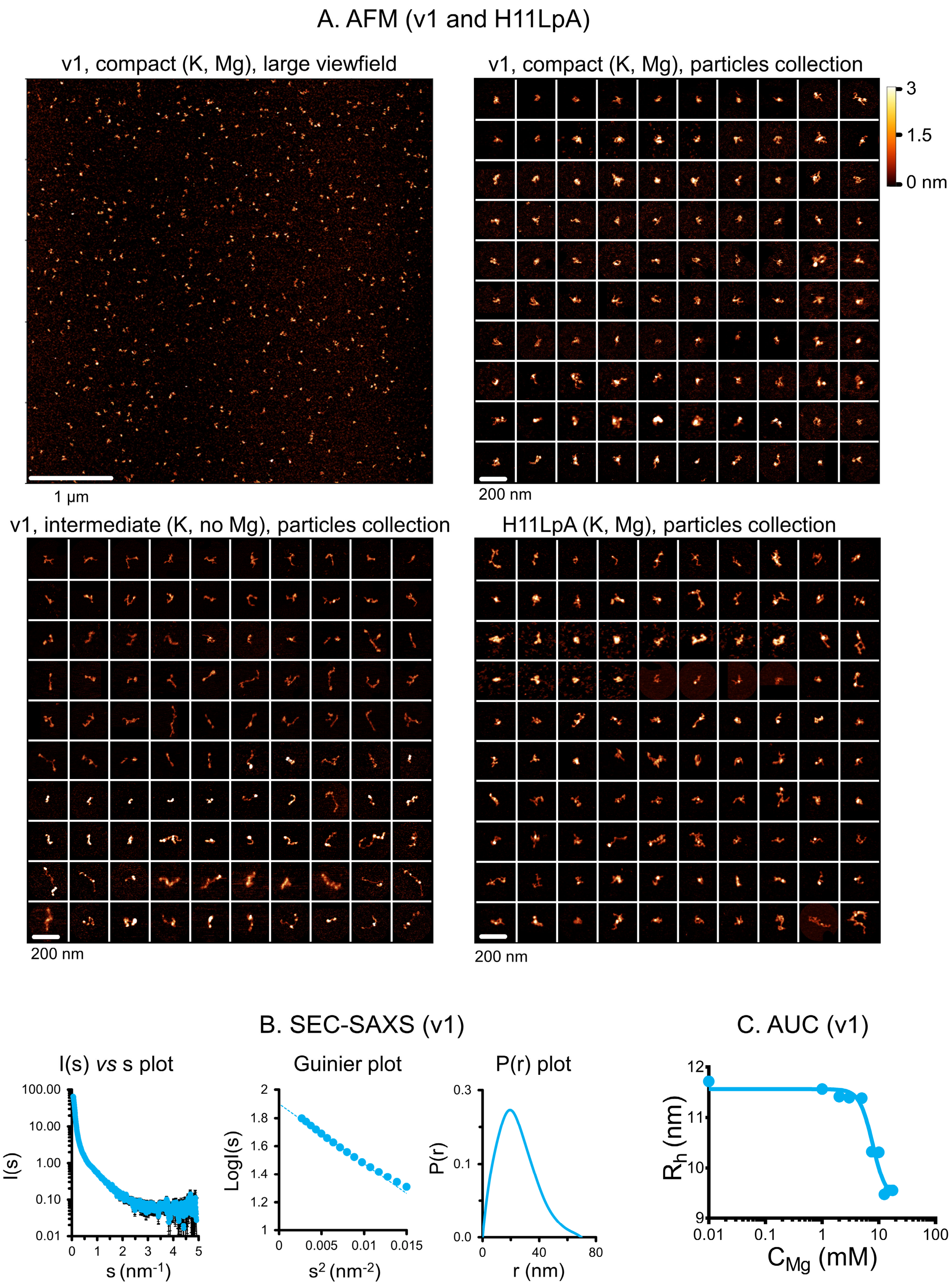
A. AFM (v1 and H11LpA)
v1, compact (K, Mg), large viewfield
v1, compact (K, Mg), particles collection
3
1.5
0 nm
1 μm
200 nm
v1, intermediate (K, no Mg), particles collection
H11LpA (K, Mg), particles collection
200 nm
200 nm
B. SEC-SAXS (v1)
I(s) vs s plot
Guinier plot
P(r) plot
I(s)
s (nm-1)
LogI(s)
s2 (nm-2)
P(r)
r (nm)
C. AUC (v1)
Rh (nm)
CMg (mM)

Supplemental Tables

Table S1: Hydrodynamic parameters of v1 (Related to Fig. 1, 5 and 6). R_g indicates the radius of gyration, R_h the hydrodynamic radius, D_{max} the maximum particle size obtained by SAXS, $C_{Mg1/2}$ the magnesium concentration at which v1 reaches half compaction, n_{Hill} the Hill coefficient of the fit to the AUC compaction curve, and D_{av} the average particle size obtained by AFM from PSD analyses.

Parameter	Technique	MEG3v1 state	Value
R_g	SEC-SAXS	intermediate (K^+ only)	18 nm
D_{max}	SEC-SAXS	intermediate (K^+ only)	70 nm
R_h	DLS	intermediate (K^+ only)	14 nm ± 7 nm
R_h	AUC	intermediate (K^+ only)	12 nm ± 0.1 nm
R_h	AUC	compact (10 mM Mg^{2+})	10 nm ± 0.1 nm
R_h	AUC	compact (50 mM Mg^{2+})	8.8 nm ± 0.3 nm
$C_{Mg1/2}$	AUC	Mg^{2+} titration (0-50 mM)	6.9 ± 0.35 mM
n_{Hill}	AUC	Mg^{2+} titration (0-50 mM)	3.9 ± 0.6
R_g / R_h		intermediate (K^+ only)	1.3 – 1.5
D_{av}	AFM	intermediate (K^+ only)	~85 nm
D_{av}	AFM	compact (10 mM Mg^{2+})	~65 nm

Table S2: Evolutionary conservation of the MEG3 exons (Related to Fig. 2, provided as a supplementary Excel file).

Table S3: Sequences of primers used in qRT-PCR (Related to Fig. 4).

Target	Primer name	Sequence (5'→3')
MEG3 D1 (Ex2)	13F_112_D1Ex2	GTCTCTCCTCAGGGATGAC
	14R_191_D1Ex2	TTGGCAGCAGCTCAGCA
MEG3 D2 (Ex3)	15F_230_D2Ex3	GAGCACGGTTTCCTGGAT
	Meg3RT22	CTGGCTGGTCAGTTCCGGTC
MEG3 D3 (Ex3)	01F_MEG3_Ex3	TCGATGAGAGCAACCTCCTA
	02R_MEG3_Ex3	TGCTGATCACCTCCTCTATG
MEG3 D4 (Ex5)	08F_951_Ex5	GGCCTGTCTACACTTGCTG
	05R_1029_D4	GGAGTAGAGCGAGTCAGGAA
MEG3 D5 (Ex10)	09F_1077_Ex10	GGCTGAAGAACTGCGGAT
	10R_1179_Ex10	AACCAGGAAGGAGACGAGAG
Neomycin	pcDNA3-F-Neo	TGGATTGCACGCAGGTTCT
	pcDNA3-R1-Neo	GGACAGGTCGGTCTTGACA
Actin	ACTB_Fwd	TTCCAGCAGATGTGGATCAG
	ACTB_Rev	GGTGTAACGCAACTAAGTCA
TP53 (p53)	03-F-cds-p53	CCTCAGCATCTTATCCGAGTG
	04-R-cds-p53	ACAGTCAGAGCCAACCTCA
BAX	BAX_Fwd	CACCAGCTCTGAGCAGATC
	BAX_Rev	GCTGCCACTCGGAAAAAG
CDKN1A (p21)	19-F-exon-p21	TGCCCAAGCTCTACCTTC
	20-R-exon-p21	GACAGTGACAGGTCCACAT
GADD45A	01F_GADD45	CCGACAATGTGACCTTCTG
	02R_GADD45	CGCACTATGTCGATGTCGTTC
GDF15	03F_GDF15	GCTACGAGGACCTGCTAAC
	04R_GDF15	CTTCTGGCGTGAGTATCCG
NOXA	NOXA_Fwd2	GGAAGTCGAGTGTGCTACTCAA
	NOXA_Rev2	CAGGTTCCTGAGCAGAAGAGTT

Table S4: Antibodies used for Western Blot analysis (Related to Fig. 4).

Antibody Specificity	Species and Isotype	Amount used	Catalog Number	Source
BAX (D2D)	Mouse IgG1	1:200	sc-20067	Santa Cruz
p21 (187)	Mouse IgG1	1:277	sc-817	Santa Cruz
p53 (DO-1)	Mouse IgG2a	1:200	sc-126	Santa Cruz
actin	Rabbit IgG	1:1000	ab1801	Abcam
mouse IgG1 Alexa Fluor 647	Goat IgG	1:1000	A-21240	Thermo
mouse IgG (H+L) Alexa Fluor 647	Goat IgG	1:1000	A-32728	Thermo
rabbit IgG Alexa Fluor 488	Goat IgG	1:1000	A-32731	Thermo

SUPPLEMENTAL FILES

Data File 1: Sequence alignments of v1 (Related to Fig. 2). The first alignment in the file, named "E3-clustal_19sequences.sto" reports the Clustal omega alignment of 19 seed sequences of E3 (~D2-D3); the second alignment in the file, named "E3-Infernal_41sequences.sto" reports the Infernal alignment of 41 sequences of E3 (~D2-D3); the third alignment in the file, named "E1E2-Clustal_33sequences.sto" reports the Clustal omega alignment of 33 sequences of E1-E2 (~D1); the fourth alignment in the file, named "E12-Clustal_40sequences.sto" reports the Clustal omega alignment of 40 sequences of E12.
